# Non-Equilibrium Quantum Brain Dynamics: Water Coupled with Phonons and Photons

**DOI:** 10.3390/e26110981

**Published:** 2024-11-15

**Authors:** Akihiro Nishiyama, Shigenori Tanaka, Jack Adam Tuszynski

**Affiliations:** 1 Graduate School of System Informatics, Kobe University, 1-1 Rokkodai, Nada-ku, Kobe 657-8501, Japan; 2 Dipartimento di Ingegneria Meccanica e Aerospaziale, Politecnico di Torino, Corso Duca degli Abruzzi 24, 10129 Turin, Italy; 3 Department of Physics, University of Alberta, 11335 Saskatchewan Dr NW, Edmonton, AB T6G 2M9, Canada; 4 Department of Data Science and Engineering, The Silesian University of Technology, 44-100 Gliwice, Poland

**Keywords:** Quantum Brain Dynamics, Water, Phonon, Acoustic Super-radiance, Kadanoff–Baym Equation, Entropy

## Abstract

We investigate Quantum Electrodynamics (QED) of water coupled with sound and light, namely Quantum Brain Dynamics (QBD) of water, phonons and photons. We provide phonon degrees of freedom as additional quanta in the framework of QBD in this paper. We begin with the Lagrangian density QED with non-relativistic charged bosons, photons and phonons, and derive time-evolution equations of coherent fields and Kadanoff–Baym (KB) equations for incoherent particles. We next show an acoustic super-radiance solution in our model. We also introduce a kinetic entropy current in KB equations in 1st order approximation in the gradient expansion and show the H-theorem for self-energy in Hartree–Fock approximation. We finally derive conserved number density of charged bosons and conserved energy density in spatially homogeneous system.

## 1. Introduction

The physical mechanism of memory involving our subjective experience in a brain is still an open question. According to generally accepted understanding in neuroscience [1], learning and memory are both linked to synaptic plasticity, which are represented at a neuronal level by long-term potentiation (LTP). However, there is a logical gap in this type of explanation because while synaptic membrane components are transient, memories of events and experience can last many years, even a lifetime. Therefore, a mechanism behind memory-related synaptic activity must be physically converted into a more enduring form, e.g., at a molecular level within post-synaptic dendritic spines, shafts and cell bodies. A specific memory code was proposed in a computational paper [2] to involve phosphorylation of neuronal microtubules (MTs) using the CaMKII enzyme. This is inspired by a mechanism present in communication technology, whereby a code converts information from one form of representation to another. The CaMKII-MT interaction was hypothesized to provide such a biomolecular code for memory in brain neurons and a conclusion was drawn that the information capacity of neuronal MTs is enormous and can easily explain the number of bits of information that could encode all sensory input data over a human lifetime. However, there could be an even more fundamental physical mechanism of information encoding in the human brain that could surpass that proposed by Craddock et al. and can act at the quantum level [3], which would also be of fundamental importance to our understanding of higher cognitive functions such as qualia [4]. The phrase ‘higher cognitive functions’ is meant to represent an evolutionary development not specific human activities. Qualia are universal but subjective instances of conscious experience such as the perception of redness of an apple or bitterness of the taste of an espresso. It is generally argued that qualia are perceptions of the human mind whose physics-based explanation so far has eluded scientists and hence has been termed the hard problem of consciousness by Chalrmers [5]. Currently, the most generally accepted view of memory encoding is by long-term potentiation of synaptic connections being strengthened when activated by a signal being transmitted between neurons [6]. The hypothesis formulated by Craddock et al delves deeper into this issue by proposing a specific encoding of information in terms of phosphorylation patterns on neuronal microtubule surfaces implemented by the enzyme CaMKII which has been experimentally validated as an active motor protein involved during the process of memory formation [7]. Here, as first suggested by Umezawa et al. [8], we explore the possibility of an even more fundamental approach to memory formation in terms of quantum fields generated by the brain. To the best of our knowledge, there is no other specific physics-based theory of memory encoding which would explore a fundamental mechanism at a quantum level with a possible exception of OrchOR [9] which links it to quantum wave function collapse due to gravitational self-interactions involving tubulin units of microtubules. Our model can be viewed as somewhat related to this perspective since we consider microtubules as the fundamental substrates of the quantum fields but we also include phonons and photons as well as the water environment, all of which makes it a more realistic setting for this physical model. It is important to note in this connection that several properties of memory in a brain different from computer memory are observed [10], namely sequential patterns, auto-associative recalling, storage in a hierarchy, memorized patterns in an invariant form. Memory is diffused in a whole brain, as a result memory is robust against lesions in a brain [11,12]. To explain these features of memory, Pribram proposed the holographic brain theory as a candidate of theory of memory and perception [13,14]. Holography, a technique to record 3-dimensional information on medium invented by Gabor [15], can describe various properties of memory in a brain as listed above. Jibu and Yasue, who collaborated with Pribram, studied Quantum Brain Dynamics (QBD), that is Quantum Field Theory (QFT) of the brain involving water electric dipole fields and photon fields [16], known as Jibu–Yasue approach distinguished from Penrose–Hameroff approach [17].

Quantum Field Theory provides a fundamental approach to describe the nature [18]. It is applied to a variety of phenomena in elementally particle physics, nuclear physics, cosmology, condensed matter physics and furthermore biology. QFT is distinguished from Quantum Mechanics since QFT describes both macroscopic matter in classical mechanics and microscopic degrees of freedom in Quantum Mechanics. QFT approach to memory in a brain is originated with the monumental work by Ricciardi and Umezawa in 1967 [19] by adopting the concept of spontaneous breakdown of symmetry (SBS). Memory is represented by vacua emerging in SBS, or macroscopic ordered patterns described in the framework of QFT. The theory is further developed by Stuart et al. [8,20], namely non-local memory storage, stability of vacua for long-term memory, metastable states for short-term memory, and memory recalling mechanism due to excitation of Nambu–Goldstone quanta emerging in SBS, which are described in Takahashi model. They proposed quantum degrees of freedom, corticons and exhange bosons, to describe quantum phenomena in a brain. What concrete degrees of freedom for corticons and exchange bosons are was not given in this stage. In 1968, the Bose–Einstein condensation in biological systems involving coherence with long-range correlations is proposed by Fröhlich, referred as Fröhlich condensation [21,22]. In 1976, Davydov and Kislukha proposed a soliton solution propagating along the alpha-helix structures of protein chains and DNA, called the Davydov soliton [23]. The Fröhlich condensation and the Davydov soliton solution emerge as static and dynamical properties, respectively, in a non-linear Schrödinger equation with an equivalent quantum Hamiltonian [24]. Around the same time, Pribram proposed the holographic brain theory [13,14]. Del Giudice et al. proposed to apply QFT to biological systems in 1980s [25,26,27,28,29]. In 1990s, Jibu and Yasue, collaborators of Pribram, proposed quantum concrete degrees of freedom in a brain, namely water rotational dipole fields and photon fields [16,30,31,32,33,34,35,36]. When water dipoles are aligned in the same direction, the spontaneous breakdown of rotational symmetry occurs and new vacua in SBS representing memory storage in a brain emerges. Excitation of incoherent photons on the vacua represents memory recalling in this theory. Vitiello showed that a huge memory capacity is achieved in the vacua by regarding the brain as an open system and adopting two-mode squeezed coherent states for Nambu–Goldstone bosons [37]. Vitiello also showed that squeezed coherent states are isomorphic to fractals in the nature [38,39]. For example, trajectories on logarithmic spirals are written by both damped and amplified oscillators with the closed system Lagrangian which is transformed to the Hermite Hamiltonian. In imposing the quantization on positions and momenta, and rewriting creation and annihilation operators of particles, the quantum states are described by two-mode squeezed coherent states in time-evolution. Quantum states composed of Nambu–Goldstone bosons in an open system evolve among squeezed coherent states corresponding to fractal. Holographic approach is also proposed by adopting coherent waves of super-radiance [33,40]. Combining QBD and holography, we can describe holographic memory storage in QBD involving properties of diversity, non-locality, stability, sequential patterns, auto-associative recalling, storage in a hierarchy, and memorized patterns in an invariant form [40].

Whether or not our brain can be described by the language of holography will be investigated by external stimuli to brain. Beauchamp et al. have shown a recent experiment using invasive stimulation to change our visual subjective experience [41]. We can also propose non-invasive approach. Non-invasive stimulation to our brain has been proposed for several decades [42], starting with transcranial magnetic stimulation (TMS) by Barker [43]. There are several non-invasive stimulation methods, such as transcranial electric stimulation with direct current [44] and alternating current [45], photonic approach involving near-infrared photons [46,47], and ultrasound approach [48,49,50] applied to treat neuropsychiatric diseases. The ultrasound-mediated drug delivery and biomarker release are also proposed as the ultrasound therapy [51]. The ultrasound therapy is adopted to destruct tumors and generate anti-tumor immune responses. When we adopt ultrasound approach, we need QFT of phonons [52,53,54,55,56] which should be extended for non-invasive neural stimulation of water-phonon-photon systems. We will further adopt reservoir computing or morphological computation [57,58,59] as a control theory of holograms via ultrasound by developing QFT in a hierarchy representing multiple layers, such as scalp, skull, dura, celebrospinal fluid covering our neocortex.

We aim to provide a theoretical formulation of water coupled with phonons and photons in QBD. This paper provides the extension of our previous approaches to QBD involving phonons, quanta of sound. We can trace a full dynamics of coherent sound fields and incoherent phonons in a brain, where sound fields decay to incoherent phonons in non-equilibrium QFT approach, for example. First we introduce the Lagrangian density of charged bosons as water degrees of freedom, phonons and photons. We refer to Keppler’s approach to describe glutamate by charged Bose fields [60]. We describe water degrees of freedom by charged Bose fields. We also refer to [61] to represent phonon fields. Time-evolution equations of coherent fields and quantum fluctuations are derived in this framework. Next we derive an acoustic super-radiance solution in our model by assuming coherence inside a microtubule [27]. Green’s functions for quantum fluctuations obey the Kadanoff–Baym equations [62,63,64] for charged bosons, phonons and photons. We also introduce a kinetic entropy current for incoherent charged bosons (water), phonons and photons in 1st order approximation in the gradient expansion, and provide a proof of H-theorem for Hartree–Fock approximation of interaction. Finally we provide time-evolution equations in spatially homogeneous systems and show concrete forms of conserved charge and energy. Our theory will be extended to the control theory of holograms in QBD using external ultrasound waves. Using reservoir computing or morphological computation theory, we can develop a non-invasive method to manipulate our subjective experiences and check whether or not our brain adopts the language of holography.

This paper is organized as follows. In Section 2, we provide background and motivation of application of QFT to biology, especially a brain. In Section 3, we introduce a Lagrangian density of QBD with phonons and show 2-Particle-Irreducible effective action. In Section 4, we show an acoustic super-radiance solution in this model. In Section 5, we show a summary for a kinetic entropy current in 1st order approximation in the gradient expansion and the H-theorem for self-energy in Hartree–Fock approximation. In Section 6, we write time-evolution equations in spatially homogeneous system and show conserved charge and energy density. In Section 7, we discuss our results. In Section 8, concluding remarks and perspectives are provided. The natural unit with the light speed, the Planck constant *ℏ* and Boltzmann constant set to be 1 is adopted. The metric tensor is $\eta_{\mu \nu }=\mathrm{diag}\left(1,-1,-1,-1\right)$ with space-time subscript μ,ν=0,1,2,3 and spatial subscript i,j,k=1,2,3.

## 2. Quantum Field Theory to Brain

In this section, we provide background and motivation to apply Quantum Field Theory (QFT) to a brain.

In conventional neuroscience, the synaptic plasticity between neurons describes memory in a brain. However, the synaptic plasticity is transient, and synaptic connections change in a few days [65,66]. In addition, this type of memory might not be robust against lesions in a brain since memory will be lost when one of the synaptic connections is destructed. If we adopt the synaptic plasticity, we cannot explain memory in a single cell organism [67]. We then need cytoskeletons inside cells to explain the mechanism of memory and information processing in a single cell organism. One of the candidates in cytoskeletons corresponds to microtubules. Phosphorylation in microtubules in [2] can be described as bits, which can be enhance the memory capacity in a brain compared with that of the synaptic plasticity.

However, the phosphorylation in microtubules appears in local events. We require diffused non-local features of memory in a brain. Non-locality of memory is suggested by Lashley [12] using lesions of brains of rats. Even if parts of the brain are damaged, rats can perform tasks. We require the processes to extend microscopic events to macroscopic features of the size of the brain. We might need tryptophan mega-networks for macroscopic events [68]. Or, we can also consider water molecules coupled with photons and phonons covering the whole brain, in which local memories in phosphorylation of microtubules are converted to optical information, such as holography. We then need coherent light (and sound) to achieve interference patterns in holography. Quantum Brain Dynamics (QBD) adopts water degrees of freedom and photons, and suggests that the new vacua emerging in spontaneous symmetry breaking where water dipoles are all aligned in the same direction. As diamond crystals and magnets emerging in breakdown of symmetry are stable at a room temperature, aligned water dipoles might be stable. The Exclusion Zone (EZ) water around hydrophilic surfaces is discovered in experiments [69,70,71]. The EZ water corresponds to the coherent water as suggested by Del Giudice et al. [72]. The size of domains composed of the EZ water is the order of 50μm. This value corresponds to the inverse of energy difference 1/I=2/2I−0/2I=4 meV (*I*: the moment of inertia of a water molecule) between excited states of rotational motion with angular momentum squared =2 and the ground state with angular momentum zero, which suggests the significance of the molecular orientations of water. The EZ water with the size 50μm might emerge around hydrophilic surfaces in and around neurons.

When we adopt the new vacua for aligned dipoles, we can propose holographic approach in the framework of QBD [40]. Holography adopts recording of interference patterns of two incident coherent lights imposed with different angles on holograms. The holograms need not to be patterns composed of curves for interference of waves, such as sine curves. We can also use binary patterns with small and large transmittance, called binary holograms [73,74,75]. Holographic memory storage with two patterns of aligned dipoles in coherent domains and water dipoles in the random orientations can be used as binary holograms. Both light or sound holography are possible with changing transmittance of light or sound for water system. These binary holograms with stable vacua involving aligned dipoles and states of random dipoles will be more stable than interference patterns with sine curves. Holographic memories are robust against damages of parts, which suggests that the whole image will be reconstructed by undamaged parts in holograms. Memory retrieval is achieved by imposing photons (or phonons for sound holography) on the holograms with the same angle as coherent lights (or sounds) in recording. Finite number of excitations of photons cannot break the vacua of aligned dipoles [18]. Human memories are modified by recalling and thinking. To rewrite the holograms involving the vacua of aligned dipoles, we require to impose coherent light or sound fields involving condensation of an infinite number of photons or phonons on holograms. Or we need to impose coherent lights or sounds in the different angles on holograms where multiple memories are recorded in the same recording media composed by water molecules. Changing the angles of incident photons or coherent light, our memory retrieval will be modified.

Tegmark suggested that coherence cannot be maintained in physiological conditions [76]. However, his expected value for coherent time is extremely short $∼10^{-20}\;s$. The problem of his analysis is first to adopt the mass of a water molecule as ∼18×940MeV. To investigate the macroscopic vacua of aligned water dipoles in QBD, we need to adopt the inverse of the moment of inertia ∼4meV, or the mass of polaritons emerging in water fields and photon fields. Furthermore, time scales for decoherence are divided by the number of surrounding Na ions $10^{6}$, which is a strange procedure. He lacks the idea that the brain is an open system, with continuous energy supply to physiological system. In the Fröhlich model, the physical system connected with an energy supply and a heat bath is considered as significant component [24]. The Fröhlich condensates might emerge in microtubules [77,78]. In the open system, the balance of decoherence and error corrections for quantum coherence can be used to maintain coherence of the physical system with continuous flow of external energy. His estimations lack these types of analysis, as a result the his estimated value of the coherence times are unrealistically small. His criticism of the quantum coherence in microtubules has been strongly rebutted by Hagan et al. [79]. Moreover, in a recent experimental paper [80], evidence was presented for the presence of long-lived (5 ns) collective quantum excitations in microtubules.

In the analysis for quantum systems, we can adopt Schrödinger equations in quantum mechanics for the atomic and molecular system. We then need to describe absorption and emission of photons and phonons. Cooperative behaviors of many-body systems for atoms and molecules can be described by QFT involving Gross–Pitaevskii equation extended from the one-body Schrödinger equation. The QFT approach includes both microscopic quantum mechanical degrees of freedom and macroscopic cooperative behaviors of quanta. We consider effective charges for molecules involving their polarization and dipole moment [60], where we can also adopt vanishing charge. Our approach includes any effective charges where we can change several charge parameters for quantum states of water molecules, such as rotations, stretching, vibrations, and so on. These water molecules are coupled with photons and phonons, or cooperatively coherent light and sound. We especially describe aspects of sound with quanta, phonons in this paper. This paper is the extension of our QBD approach with including phonons. Our approach adopts both coherent photon and phonon fields and their quantum fluctuations which are required for a full understanding the Fröhlich condensate [81]. Furthermore non-equilibrium collective behaviors for phonon condensates described in [82] are also included in our approach.

## 3. Lagrangian Density and 2-Particle-Irreducible Effective Action

In this section, we provide a Lagrangian density for charged bosons, phonons and photons, show 2-Particle-Irreducible Effective Action and derive time-evolution equations. We adopt the background field method [83,84,85,86]. Using the Lagrangian, we can derive the Gross–Pitaevskii equation as extension of Schrödinger equation to describe collective properties of water molecules. The equation describes various quantum states of water molecules, including conformational states related with strongly bonded or less bonded states observed in ranges of wavelength from 1300nm to 1600nm as shown in [87], and rotational degrees of freedom in wavelength 310μm, for example.

The Lagrangian density is given by,

(1) $$\begin{array}{ccc} L & = & -\frac{1}{4}F^{\mu \nu }\left[A+a\right]F_{\mu \nu }\left[A+a\right]-\frac{1}{2\xi }{\left(\partial^{\mu }a_{\mu }\right)}^{2}\\ & & +\psi^{*}\left(i\frac{\partial }{\partial x^{0}}+e\left(A^{0}+a^{0}\right)+\frac{{\left(\nabla_{i}-ie\left(A_{i}+a_{i}\right)\right)}^{2}}{2m}\right)\psi \\ & & +\frac{1}{2}\left[{\left(\frac{\partial Q_{a}^{i}}{\partial x^{0}}\right)}^{2}-v_{2,ij}\left(\frac{\partial Q_{a}^{i}}{\partial x^{k}}\right)\left(\frac{\partial Q_{a}^{j}}{\partial x^{k}}\right)\right]+\frac{1}{2}\left[{\left(\frac{\partial Q_{o}^{i}}{\partial x^{0}}\right)}^{2}-\Omega_{2,ij}Q_{o}^{i}Q_{o}^{j}\right]\\ & & -g_{aL}\psi^{*}\psi \nabla_{i}Q_{aL}^{i}+ig_{aT}\left(\psi^{*}\left(\partial_{i}\psi \right)-\left(\partial_{i}\psi^{*}\right)\psi -2ie\psi^{*}\psi \left(A_{i}+a_{i}\right)\right)Q_{aT}^{i}\\ & & -g_{oL}\psi^{*}\psi \nabla_{i}Q_{oL}^{i}+ig_{oT}\left(\psi^{*}\left(\partial_{i}\psi \right)-\left(\partial_{i}\psi^{*}\right)\psi -2ie\psi^{*}\psi \left(A_{i}+a_{i}\right)\right)Q_{oT}^{i}, \end{array}$$

with electromagnetic tensor $F_{\mu \nu }\left[A\right]=\partial_{\mu }A_{\nu }-\partial_{\nu }A_{\mu }$ with background photon fields $A_{\mu }$ and quantum fluctuations $a_{\mu }$, complex charged Bose fields $\psi^{*}$ and ψ, acoustic phonon fields $Q_{a}^{i}=-Q_{a,i}$, optical phonon fields $Q_{o}^{i}=-Q_{o,i}$, gauge fixing parameter ξ, elementary charge *e*, sound velocity matrix $v_{2,ij}$ including eigenvalues of velocity squared for transverse and longitudinal modes for acoustic phonons ($v_{T}^{2}$ and $v_{L}^{2}$), frequency matrix $\Omega_{2,ij}$ including eigenvalues of frequency squared for transverse and longitudinal modes for optical phonons ($\Omega_{T}^{2}$ and $\Omega_{L}^{2}$), coupling constants between charged bosons and acoustic longitudinal phonons $g_{aL}$ and transverse phonons $g_{aT}$, and coupling constants between charged bosons and optical longitudinal phonons $g_{oL}$ and transverse phonons $g_{oT}$. Longitudinal phonons are coupled with the gradient of density of charged bosons $\nabla_{i}\left(\psi^{*}\psi \right)$, while transverse phonons coupled with the flow of charged bosons $\left(\psi^{*}\left(\partial_{i}\psi \right)-\left(\partial_{i}\psi^{*}\right)\psi -2ie\psi^{*}\psi \left(A_{i}+a_{i}\right)\right)$. Our Lagrangian is based on Quantum Electrodynamics for non-relativistic charged bosons. We can also charge *e* to any effective values. We include phonon degrees of freedom in QBD theory in this paper to investigate coherent sound emitted from microtubules as super-radiance as shown in the next section and provide non-equilibrium theory for incoherent phonons. Both acoustic and optical phonons are introduced as quantum fields and coupled with density of water molecules and flow of water molecules.

The above Lagrangian is invariant under the type I gauge transformation given by,

(2) $$\begin{array}{c} \psi \left(x\right)\to e^{i\alpha (x)}\psi \left(x\right),\;\;\psi^{*}\left(x\right)\to e^{-i\alpha (x)}\psi^{*}\left(x\right),\;\;A_{\mu }\left(x\right)\to A_{\mu }\left(x\right)+\frac{1}{e}\partial_{\mu }\alpha \left(x\right),\;\;a_{\mu }\left(x\right)\to a_{\mu }\left(x\right). \end{array}$$

We shall set gauge fixing $a^{0}=0$ and ξ=1. Closed-time path formalism is adopted to describe non-equilibrium quantum dynamics [88,89]. Starting with the Lagrangian density in Equation (1), we can write 2-Particle-Irreducible (2PI) Effective Action [90,91,92] as,

(3) $$\begin{array}{ccc} \Gamma_{2\mathrm{PI}}\left[A,{\bar{a}}_{i},\bar{\psi },{\bar{\psi }}^{*},{\bar{Q}}_{a},{\bar{Q}}_{o},\Delta ,D\right] & = & \int_{C}d^{4}xL\left[A,{\bar{a}}_{i},\bar{\psi },{\bar{\psi }}^{*},{\bar{Q}}_{a},{\bar{Q}}_{o}\right]\\ & & +\frac{i}{2}\mathrm{Tr}\ln D^{-1}+\frac{1}{2}\mathrm{Tr}\left(iD_{0}^{-1}D\right)+i\mathrm{Tr}\ln\Delta^{-1}+\mathrm{Tr}\left(i\Delta_{0}^{-1}\Delta \right)\\ & & +\frac{1}{2}\Gamma_{2}\left[A_{i}+{\bar{a}}_{i},\bar{\psi },{\bar{\psi }}^{*},{\bar{Q}}_{a},{\bar{Q}}_{o},\Delta ,D\right], \end{array}$$

where C represents the closed-time path contour in path ‘1’ from −∞ to ∞ and path ‘2’ from ∞ to −∞. The bar represents expectation values $\bar{\psi }=\langle \psi \rangle =\mathrm{Tr}\left(R\psi \right)$ for an arbitrary density matrix R. When we set $R∼e^{-H/T}$ with Hamiltonian H and temperature T=310K, we can include contributions of finite-temperature medium. The D(x,y) represents matrix of Green’s functions for photons and phonons given by,

(4) $$\begin{array}{ccc} D(x,y) & = & \left(\begin{array}{ccc} D_{ij}\left(x,y\right) & D_{\gamma a,ij}\left(x,y\right) & D_{\gamma o,ij}\left(x,y\right)\\ D_{a\gamma ,ij}\left(x,y\right) & D_{aa,ij}\left(x,y\right) & D_{ao,ij}\left(x,y\right)\\ D_{o\gamma ,ij}\left(x,y\right) & D_{oa,ij}\left(x,y\right) & D_{oo,ij}\left(x,y\right) \end{array}\right), \end{array}$$

with definitions $D_{ij}\left(x,y\right)=\langle T_{C}\delta a_{i}\left(x\right)\delta a_{j}\left(y\right)\rangle$ with time-ordered product $T_{C}$ and $\delta a_{i}=a_{i}-{\bar{a}}_{i}$, $D_{a\gamma ,ij}\left(x,y\right)=\langle T_{C}\delta Q_{a,i}\left(x\right)\delta a_{j}\left(y\right)\rangle$ with $\delta Q_{a,i}=Q_{a,i}-{\bar{Q}}_{a,i}$, $D_{aa,ij}\left(x,y\right)=\langle T_{C}\delta Q_{a,i}\left(x\right)\delta Q_{a,j}\left(y\right)\rangle$, $D_{oo,ij}\left(x,y\right)=\langle T_{C}\delta Q_{o,i}\left(x\right)\delta Q_{o,j}\left(y\right)\rangle$ with $\delta Q_{o,i}=Q_{o,i}-{\bar{Q}}_{o,i}$ and so on. In matrix notation of closed-time path, $D_{a\gamma ,ij}\left(x,y\right)$ represents,

(5) $$\begin{array}{ccc} D_{a\gamma ,ij}\left(x,y\right) & = & \left(\begin{array}{cc} D_{a\gamma ,ij}^{11}\left(x,y\right) & D_{a\gamma ,ij}^{12}\left(x,y\right)\\ D_{a\gamma ,ij}^{21}\left(x,y\right) & D_{a\gamma ,ij}^{22}\left(x,y\right) \end{array}\right)=\left(\begin{array}{cc} \langle T\delta Q_{a,i}\left(x\right)\delta a_{j}\left(y\right)\rangle & \langle \delta a_{j}\left(y\right)\delta Q_{a,i}\left(x\right)\rangle \\ \langle \delta Q_{a,i}\left(x\right)\delta a_{j}\left(y\right)\rangle & \langle \tilde{T}\delta Q_{a,i}\left(x\right)\delta a_{j}\left(y\right)\rangle \end{array}\right), \end{array}$$

with time-ordered product T and anti-time-ordered product $\tilde{T}$. The Δ(x,y) represents,

(6) $$\begin{array}{c} \Delta \left(x,y\right)=\langle T_{C}\delta \psi \left(x\right)\delta \psi^{*}\left(y\right)\rangle , \end{array}$$

with $\delta \psi^{\left(*\right)}=\psi^{\left(*\right)}-{\bar{\psi }}^{\left(*\right)}$. The $iD_{0}^{-1}\left(x,y\right)$ represents the matrix,

(7) $$\begin{array}{ccc} iD_{0}^{-1}\left(x,y\right) & = & \left(\begin{array}{ccc} -\left(\partial_{x}^{2}+\frac{e^{2}{\bar{\psi }}^{*}\left(x\right)\bar{\psi }\left(x\right)}{m}\right)\delta_{ij} & -2eg_{aT}{\bar{\psi }}^{*}\bar{\psi }\left(\delta_{ij}-\frac{\partial_{i}\partial_{j}}{\partial_{k}^{2}}\right) & -2eg_{oT}{\bar{\psi }}^{*}\bar{\psi }\left(\delta_{ij}-\frac{\partial_{i}\partial_{j}}{\partial_{k}^{2}}\right)\\ -2eg_{aT}{\bar{\psi }}^{*}\bar{\psi }\left(\delta_{ij}-\frac{\partial_{i}\partial_{j}}{\partial_{k}^{2}}\right) & -\left(\partial_{0}^{2}\delta_{ij}-v_{2,ij}\partial_{k}^{2}\right) & 0\\ -2eg_{oT}{\bar{\psi }}^{*}\bar{\psi }\left(\delta_{ij}-\frac{\partial_{i}\partial_{j}}{\partial_{k}^{2}}\right) & 0 & -\left(\partial_{0}^{2}\delta_{ij}+\Omega_{2,ij}\right) \end{array}\right)\\ & & \times \delta_{C}\left(x-y\right), \end{array}$$

$i\Delta_{0}^{-1}\left(x,y\right)=\frac{\delta^{2}\int_{w}L\left(w\right)}{\delta \psi^{*}\left(x\right)\delta \psi \left(y\right)}$ represents,

(8) $$\begin{array}{ccc} i\Delta_{0}^{-1}\left(x,y\right) & = & (i\frac{\partial }{\partial x^{0}}+eA^{0}+\frac{{\left(\nabla_{i}-ie\left(A_{i}+{\bar{a}}_{i}\right)\right)}^{2}}{2m}\\ & & -g_{aL}\left(\nabla_{x,i}{\bar{Q}}_{aL}^{i}\left(x\right)\right)+ig_{aT}\left({\bar{Q}}_{aT}^{i}\left(x\right)+{\bar{Q}}_{aT}^{i}\left(y\right)\right)\left(\partial_{x,i}-ie\left(A_{i}\left(x\right)+{\bar{a}}_{i}\left(x\right)\right)\right)\\ & & -g_{oL}\left(\nabla_{x,i}{\bar{Q}}_{oL}^{i}\left(x\right)\right)+ig_{oT}\left({\bar{Q}}_{oT}^{i}\left(x\right)+{\bar{Q}}_{oT}^{i}\left(y\right)\right)\left(\partial_{x,i}-ie\left(A_{i}\left(x\right)+{\bar{a}}_{i}\left(x\right)\right)\right))\delta_{C}\left(x-y\right), \end{array}$$

and $\frac{i\Gamma_{2}}{2}$ represents all the 2-Particle-Irreducible loop diagrams corresponding to Φ-derivable loop expansion technique in [64].

**Figure 1:**
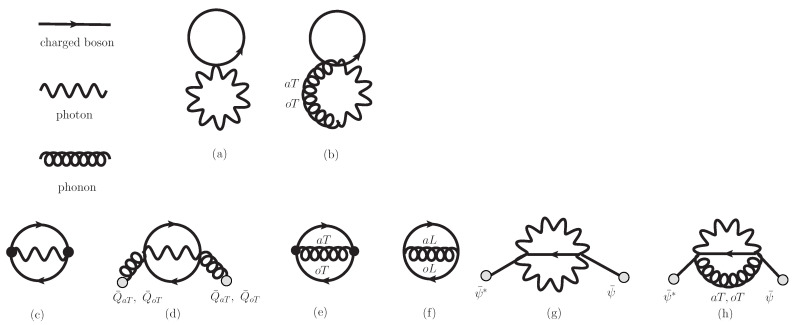
2-Particle-Irreducible loop diagrams in Hartree–Fock approximation labeled by (**a**–**h**). The diagrams (**a**,**b**) represent local terms, while diagrams from (**c**) to (**h**) represent non-local terms. Solid lines, wavy lines and curly lines represent propagation of charged bosons, photons, and phonons, respectively. The dark circles in vertices involve covariant derivatives such as $\partial_{i}-ieA_{i}$. The light circles in external lines represent background coherent fields ${\bar{Q}}_{aT}$, ${\bar{Q}}_{oT}$, ${\bar{Q}}_{aL}$, ${\bar{Q}}_{oL}$, $\bar{\psi }$, and ${\bar{\psi }}^{*}$. The aT, oT, aL, and oL represent acoustic transverse, optical transverse, acoustic longitudinal, and optical longitudinal phonons, respectively.

We adopt Hartree–Fock approximation for the $\frac{i\Gamma_{2}}{2}$. The 2-loop diagrams in $\frac{i\Gamma_{2}}{2}$ is depicted in Figure 1. We find local terms in Figure 1a inducing mass shift of charged bosons and photons and terms in Figure 1b inducing the coupling for phonon-photon exchange. Non-local terms in Figure 1c–g are depicted to represent interaction among charged bosons, photons and phonons. Differentiating $\frac{i\Gamma_{2}}{2}$ by Green’s functions, we can derive self-energy.

The time-evolution equations are given by differentiating $\Gamma_{2\mathrm{PI}}$ in Equation (3) by fields ${\bar{a}}_{i}\left(x\right)$, ${\bar{\psi }}^{\left(*\right)}\left(x\right)$, Green’s functions D(x,y) and Δ(x,y). They are given in Appendices Appendix A, Appendix C and Appendix E.

## 4. Acoustic Super-Radiance

**Figure 2:**
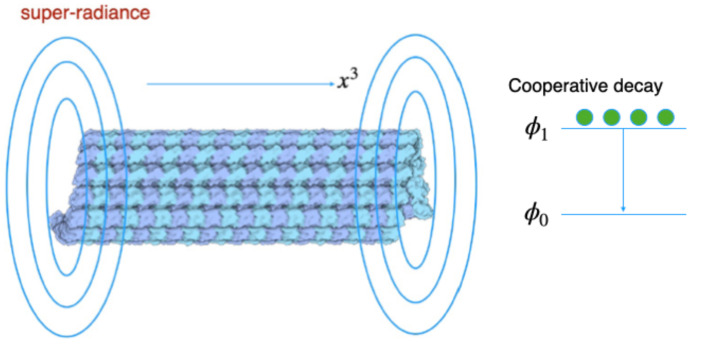
Acoustic super-radiance emitted in a radial direction via a microtubule.

In this section, we show a solution of acoustic super-radiance, cooperative coherent spontaneous sound emission from water, shown in Figure 2. We adopt a microtubule, cytoskeleton involving a cylindrical structure, as a coherent sound source. We neglect contributions of quantum fluctuations in this section. The size of coherent domains can estimated as 15nm [27]. This size corresponds to the inner diameter of microtubules. Hence microtubules are regarded as devices achieving coherent domains. We can derive acoustic super-radiance from coherent domains in microtubules emitted in a radial direction in Figure 2. It might be observed around microtubules with continuous external energy supply from mitochondria [93].

We use Equations (A2) and (A6) in Appendix A by assuming e=0, $g_{aL}=0$, $g_{oT}=0$, and $g_{oL}=0$ only for interaction between water and transverse acoustic phonons. The relation (A2) is rewritten by,

(9) $$\begin{array}{c} \left(i\frac{\partial }{\partial x^{0}}+\frac{\nabla_{i}^{2}}{2m}-2ig_{aT}{\bar{Q}}_{aT,i}\nabla_{i}+\tilde{U}\left(x\right)\right)\bar{\psi }\left(x\right)=0, \end{array}$$

where we added potential energy term $\tilde{U}\left(x\right)$ representing water molecular states. The relation (A6) is rewritten by,

(10) $$\begin{array}{ccc} \left(\partial_{0}^{2}-v_{T}^{2}\partial_{j}^{2}\right)Q_{aT,i}+ig_{aT}\left({\bar{\psi }}^{*}\nabla_{i}\bar{\psi }-\left(\nabla_{i}{\bar{\psi }}^{*}\right)\bar{\psi }\right) & = & 0. \end{array}$$

We adopt two-energy level approximation for water molecular states, namely the ground state and the 1st excited state. Water absorbs the various ranges of wavelength of photons [94], which correspond to various water molecular states including symmetric and anti-symmetric stretching modes with ranges of wavelength from 1300nm to 1600nm, and rotational motions with the order of a few meV with the wavelength ∼300μm, for example. We can set $\tilde{U}\left(x\right)$ in Equation (9) representing energy of rotational motion of water molecules or mean field energy for molecular stretching, and so on, given by approximations of interaction with photons and phonons. Since water absorbs both light and sound [87], water is also regarded as coherent light and sound sources. The Equation (9) covers those water molecular states, while the dynamics of coherent sound fields is given by Equation (10).

Using the calculations in Appendix B, we arrive at the amplitude of coherent phonon fields Q given by,

(11) $$\begin{array}{c} Q=\frac{g\bar{\Omega }N}{4\pi }{\left[\cosh\left(\frac{x^{0}-\tau_{0}}{\tau_{R}}\right)\right]}^{-1}, \end{array}$$

where the $\bar{\Omega }$ represents the energy difference between the ground state and 1st excited state, the *N* represents the number of water molecules, the *g* is given by $g\equiv mg_{aT}J_{01}$ with Equation (A28) related with transition dipole moment of water molecules, and $\tau_{R}$ represents,

(12) $$\begin{array}{c} \tau_{R}=\frac{2\pi }{g^{2}\bar{\Omega }N}\propto \frac{1}{N}, \end{array}$$

and $\tau_{0}=-\tau_{R}\ln\tan\frac{\theta_{0}}{2}$. Due to *N* water molecules cooperatively decaying in 1/N time scale, the coherent sound field has intensity of the order of $N^{2}$ instantly, representing acoustic super-radiance. Substituting the mode $\bar{\Omega }$ corresponding to water molecular states, *N* representing the number of water molecules in the states, and *g* for transition of water molecules in Equations (11) and (12) by their concrete values, we can derive the amplitude of coherent sound fields and the time scale of the acoustic super-radiance for each water molecular state.

## 5. Kinetic Entropy Current and the H-Theorem

In this section, we summarize the introduction of a kinetic entropy current and the H-theorem for 1-loop self-energy in Hartree–Fock approximation. We refer to the preceding works in [95,96,97,98,99,100,101]. The detailed calculations are given in Appendix D.

We finally arrive at,

(13) $$\begin{array}{c} \partial_{\mu }s^{\mu }=\left(\mathrm{PP}\right)+\left(\mathrm{Dia}(c)\right)+\left(\mathrm{Dia}(d)\right)+\left(\mathrm{Dia}(e)\right)+\left(\mathrm{Dia}(f)\right)+\left(\mathrm{Dia}(g)\right)+\left(\mathrm{Dia}(h)\right)\geq 0, \end{array}$$

where (PP) represents the photon-phonon exchange via water molecules. The total entropy current $s^{\mu }$ is given by Equation (A216). The H-theorem is proved in the Hartree–Fock approximation for Kadanoff–Baym equations in 1st order in the gradient expansions.

Entropy production stops in the condition,

(14) $$\begin{array}{ccc} f(p) & = & \frac{1}{e^{\frac{p^{0}-\mu_{c}}{T}}-1}, \end{array}$$

with temperature *T* and chemical potential $\mu_{c}=e\left(A^{0}-\frac{\partial^{0}\beta }{e}\right)$ for distribution function of charged bosons f(p), and,

(15) $$\begin{array}{c} f_{T}\left(k\right)=f_{L}\left(k\right)=f_{aa,T}\left(k\right)=f_{aa,L}\left(k\right)=f_{oo,T}\left(k\right)=f_{oo,L}\left(k\right)=\frac{1}{e^{\frac{k^{0}}{T}}-1}, \end{array}$$

with distribution functions for transverse photons $f_{T}$, longitudinal photons $f_{L}$, transverse acoustic phonons $f_{aa,T}$, longitudinal acoustic phonons $f_{aa,L}$, transverse optical phonons $f_{oo,T}$ and longitudinal optical phonons $f_{oo,L}$. The system composed by charged bosons, photons and phonons evolve in time with entropy producing processes.

## 6. Time-Evolution Equations in Spatially Homogeneous Systems

In this section we show time-evolution equations in spatially homogeneous system and show concrete forms of conserved charge and energy density.

First we shall rewrite Kadanoff–Baym Equations (A65), (A68), (A97), (A102), (A105), (A109), (A112), (A116) and (A126), in Appendix C. We use statistical functions $F\left(X,p\right)=\frac{G^{12}\left(X,p\right)+G^{21}\left(X,p\right)}{2}$, $F_{L}\left(X,k\right)=\frac{D_{L}^{12}\left(X,k\right)+D_{L}^{21}\left(X,k\right)}{2}$, $F_{T}\left(X,k\right)=\frac{D_{T}^{12}+D_{T}^{21}}{2}$, $F_{aa,L}\left(X,k\right)=\frac{D_{aa,L}^{12}+D_{aa,L}^{21}}{2}$, $F_{aa,T}\left(X,k\right)=\frac{D_{aa,T}^{12}+D_{aa,T}^{21}}{2}$, $F_{oo,L}\left(X,k\right)=\frac{D_{oo,L}^{12}+D_{oo,L}^{21}}{2}$, $F_{oo,T}\left(X,k\right)=\frac{D_{oo,T}^{12}+D_{oo,T}^{21}}{2}$, $d_{aa,F,T}=\frac{d_{aa,T}^{12}+d_{aa,T}^{21}}{2}$ and $d_{oo,F,T}=\frac{d_{oo,T}^{12}+d_{oo,T}^{21}}{2}$, spectral functions $\rho =i(G^{21}-G^{12})$, $\rho_{L}=i\left(D_{L}^{21}-D_{L}^{12}\right)$, $\rho_{T}=i\left(D_{T}^{21}-D_{T}^{12}\right)$, $\rho_{aa,L}=i\left(D_{aa,L}^{21}-D_{aa,L}^{12}\right)$, $\rho_{aa,T}=i\left(D_{aa,T}^{21}-D_{aa,T}^{12}\right)$, $\rho_{oo,L}=i\left(D_{oo,L}^{21}-D_{oo,L}^{12}\right)$, $\rho_{oo,L}=i\left(D_{oo,L}^{21}-D_{oo,L}^{12}\right)$, $d_{aa,\rho ,T}=i\left(d_{aa,T}^{21}-d_{aa,T}^{12}\right)$, and $d_{oo,\rho ,T}=i\left(d_{oo,T}^{21}-d_{oo,T}^{12}\right)$. Spectral functions represent which states particles are occupied, while statistical functions represent particle number density or how many particles are in the states. We also use statistical parts of self-energy $\Sigma_{F}=\frac{\Sigma_{\mathrm{nonl}}^{12}+\Sigma_{\mathrm{nonl}}^{21}}{2}$, $\Pi_{F,L}=\frac{\Pi_{\mathrm{nonl},L}^{12}+\Pi_{\mathrm{nonl},L}^{21}}{2}$, $\Pi_{F,T}=\frac{\Pi_{\mathrm{nonl},T}^{12}+\Pi_{\mathrm{nonl},T}^{21}}{2}$, $\Pi_{aa,F,L}=\frac{\Pi_{\mathrm{nonl},aa,L}^{12}+\Pi_{\mathrm{nonl},aa,L}^{21}}{2}$, $\Pi_{aa,F,T}=\frac{\Pi_{\mathrm{nonl},aa,T}^{12}+\Pi_{\mathrm{nonl},aa,T}^{21}}{2}$, $\Pi_{oo,F,L}=\frac{\Pi_{\mathrm{nonl},oo,L}^{12}+\Pi_{\mathrm{nonl},oo,L}^{21}}{2}$, $\Pi_{oo,F,T}=\frac{\Pi_{\mathrm{nonl},oo,T}^{12}+\Pi_{\mathrm{nonl},oo,T}^{21}}{2}$, $U_{aa,F,T}=\frac{U_{aa,T}^{12}+U_{aa,T}^{21}}{2}$, $U_{oo,F,T}=\frac{U_{oo,T}^{12}+U_{oo,T}^{21}}{2}$, $V_{aa,F,T}=\frac{V_{aa,T}^{12}+V_{aa,T}^{21}}{2}$, and $V_{oo,F,T}=\frac{V_{oo,T}^{12}+V_{oo,T}^{21}}{2}$, and spectral parts $\Sigma_{\rho }=i\left(\Sigma_{\mathrm{nonl}}^{21}-\Sigma_{\mathrm{nonl}}^{12}\right)$, $\Pi_{\rho ,L}=i\left(\Pi_{\mathrm{nonl},L}^{21}-\Pi_{\mathrm{nonl},L}^{12}\right)$, $\Pi_{\rho ,T}=i\left(\Pi_{\mathrm{nonl},T}^{21}-\Pi_{\mathrm{nonl},T}^{12}\right)$, $\Pi_{aa,\rho ,L}=i\left(\Pi_{\mathrm{nonl},aa,L}^{21}-\Pi_{\mathrm{nonl},aa,L}^{12}\right)$, $\Pi_{aa,\rho ,T}=i\left(\Pi_{\mathrm{nonl},aa,T}^{21}-\Pi_{\mathrm{nonl},aa,T}^{12}\right)$, $\Pi_{oo,\rho ,L}=i\left(\Pi_{\mathrm{nonl},oo,L}^{21}-\Pi_{\mathrm{nonl},oo,L}^{12}\right)$, $\Pi_{oo,\rho ,T}=i\left(\Pi_{\mathrm{nonl},oo,T}^{21}-\Pi_{\mathrm{nonl},oo,T}^{12}\right)$, $U_{aa,\rho ,T}=i\left(U_{aa}^{21}-U_{aa}^{12}\right)$, $U_{oo,\rho ,T}=i\left(U_{oo}^{21}-U_{oo}^{12}\right)$, $V_{aa,\rho ,T}=i\left(V_{aa}^{21}-V_{aa}^{12}\right)$, and $V_{oo,\rho ,T}=i\left(V_{oo}^{21}-V_{oo}^{12}\right)$.

The Kadanoff–Baym equations for charged bosons for real *F* and $\Sigma_{F}$ and pure imaginary ρ and $\Sigma_{\rho }$ are,

(16)      iG0−1(p)−Σloc−ReΣR,F+ReGR,ΣF=1iFΣρ−ρΣF,

(17) iG0−1(p)−Σloc−ReΣR,ρ+ReGR,Σρ=0,

with,

(18) $$\begin{array}{ccc} iG_{0}^{-1}\left(p\right) & = & p^{0}-\frac{p^{2}}{2m}-g_{aL}\left(\partial_{X,i}{\bar{Q}}_{aL}^{i}\right)-g_{oL}\left(\partial_{X,i}{\bar{Q}}_{oL}^{i}\right)+2g_{aT}p_{i}{\bar{Q}}_{aT}^{i}+2g_{oT}p_{i}{\bar{Q}}_{oT}^{i}. \end{array}$$

Here the subscript ‘*R*’ represents retarded parts for Green’s functions and self-energy. For example, its real part is given by ReGR=i2G11−G22. The local self-energy for charged bosons is given in Equation (A228) in Appendix E. The statistical and spectral parts in self-energy are given in Equations (A231) and (A232) in Appendix E.

Next, Kadanoff–Baym equations for photons and phonons are derived from (A65), (A68), (A97), (A102), (A105), (A109), (A112) and (A116). For statistical and spectral parts, we can derive,

(19)        iD0−1(k)−Πloc−ReΠR,L,FLP+ReDR,L,ΠF,LP=1iFLΠρ,L−ρLΠF,L,

(20) iD0−1(k)−Πloc−ReΠR,L,ρLP+ReDR,L,Πρ,LP=0,

with $iD_{0}^{-1}\left(k\right)=k^{2}-\frac{e^{2}{\left|\bar{\psi }\right|}^{2}}{m}$, and,

(21) iD0−1(k)−Πloc−ReΠR,T+ReUaa,R,T+ReUoo,R,T,FTP+ReDR,T,ΠF,T−Uaa,F,T−Uoo,F,TP=1iFTΠρ,T−ρTΠF,T−1iFTUaa,ρ,T−ρTUaa,F,T−1iFTUoo,ρ,T−ρTUoo,F,T,

(22) iD0−1(k)−Πloc−ReΠR,T+ReUaa,R,T+ReUoo,R,T,ρTP+ReDR,T,Πρ,T−Uaa,ρ,T−Uoo,ρ,TP=0.

For acoustic phonons, we can derive,

(23) iD0,aa,L−1−ReΠaa,R,L,Faa,LP+ReDaa,R,L,Πaa,F,LP=1iFaa,LΠaa,ρ,L−ρaa,LΠaa,F,L,

(24) iD0,aa,L−1−ReΠaa,R,L,ρaa,LP+ReDaa,R,L,Πaa,ρ,LP=0,

with $iD_{0,aa,L}^{-1}\left(k\right)={\left(k^{0}\right)}^{2}-v_{L}^{2}k^{2}$,

(25) iD0,aa,T−1−ReΠaa,R,T,Faa,TP+ReDaa,R,T,Πaa,F,TP+ReVaa,R,T,daa,F,TP−Redaa,R,T,Vaa,F,TP=1iFaa,TΠaa,ρ,T−ρaa,TΠaa,F,T−1idaa,F,TVaa,ρ,T−daa,ρ,TVaa,F,T,

(26)       iD0,aa,T−1−ReΠaa,R,T,ρaa,TP+ReDaa,R,T,Πaa,ρ,TP       +ReVaa,R,T,daa,ρ,TP−Redaa,R,T,Vaa,ρ,TP=0,

with $iD_{0,aa,T}^{-1}\left(k\right)={\left(k^{0}\right)}^{2}-v_{T}^{2}k^{2}$. For optical phonons, we can write Kadanoff–Baym equations by replacing the subscript ‘aa’ to ‘oo’ in Equations (23)–(26) with $iD_{0,oo,L}^{-1}\left(k\right)={\left(k^{0}\right)}^{2}-\Omega_{L}^{2}$ and $iD_{0,oo,T}^{-1}\left(k\right)={\left(k^{0}\right)}^{2}-\Omega_{T}^{2}$. We also use the auxiliary equations,

(27) iD0,aa,T−1−ReΠaa,R,T,daa,F,TP+Redaa,R,T,Πaa,F,TP=1idaa,F,TΠaa,ρ,T−daa,ρ,TΠaa,F,T,

(28)           iD0,aa,T−1−ReΠaa,R,T,daa,ρ,TP+Redaa,R,T,Πaa,ρ,TP=0,

with equations where subscripts ‘aa’ are replaced by ‘oo’.

Next we write time-evolution equation for coherent charged boson fields. It is written by,

(29) ∂0|ψ¯|2=−∂0∫p∂ReΣR(g)+(h)(p)∂p0F(p)+ReGR(p)∂ΣF(g)+(h)(p)∂p0−∫pF(p)Σρ(g)+(h)(p)i−ρ(p)iΣF(g)+(h)(p),

which provides time-evolution of the density $|\bar{\psi }{|}^{2}$. Here we have used Equation (A4) and Appendix F. We also encounter the constraint relation (A290) in Appendix F. We also use Equations (A295) for coherent photon fields, (A297)–(A300) for coherent phonon fields.

Finally, using the above relations, we can derive the total charge conservation,

(30) $$\begin{array}{ccc} \partial_{0}\left[-e\left(|\bar{\psi }{\left(X\right)|}^{2}+\int_{p}F\left(X,p\right)\right)\right] & = & 0, \end{array}$$

where we can derive the right-hand side in Equation (29) and time-derivative of $\int_{p}F\left(X,p\right)$ with integration of Equation (16) cancel. We can also derive the total energy conservation,

(31) $$\begin{array}{c} \partial_{0}E_{\mathrm{tot}}=0, \end{array}$$

with total energy density $E_{\mathrm{tot}}$ given in Appendix G.

## 7. Discussion

In this paper, we have investigated Quantum Electrodynamics (QED) for water coupled with phonons and photons corresponding to Quantum Brain Dynamics involving water, sound and light, and provided theoretical formulation in our model. Beginning with Lagrangian density of charged bosons representing water molecular states, phonons and photons, we have derived time-evolution equations for coherent photon, charged boson and phonon fields, and Kadanoff–Baym equations for incoherent photons, charged bosons and phonons. We have derived an acoustic super-radiance solution in our model. We have introduced a kinetic entropy current in 1st order approximation in the gradient expansion and shown the H-theorem for self-energy in Hartree–Fock approximation. Finally we have given time-evolution equations in spatially homogeneous system and shown concrete forms of conserved total charge and energy density.

We have adopted the similar Lagrangian density to that by Nguyen et al. [61] who introduced interaction Hamiltonian between transverse phonon fields and flow of charged bosons. They introduced the interaction Hamiltonian $H_{I}$ like,

(32) $$\begin{array}{c} H_{I}∼i\int d^{3}x\left((\psi^{*}\partial_{i}\psi -\left(\partial_{i}\psi^{*}\right)\psi \right)Q_{aT,i}, \end{array}$$

and derived collective behaviors of charged particles, namely plasmons. However, there is a serious problem in Nguyen’s model. Since $\partial_{i}\psi$ is not a covariant derivative like $(\partial_{i}-ieA_{i})\psi$, the Hamiltonian is not gauge-invariant. Then we find that charge conservation is no longer satisfied in their model. Hence for our model in this paper, we adopt gauge-invariant interaction Lagrangian involving covariant derivatives. Imposing gauge-invariance in Lagrangian, we encounter phonon–photon interaction via charged bosons in term $\psi^{*}\psi A_{i}Q_{aT,i}$ in Equation (1). Due to this term, the Kadanoff–Baym equations for photons and phonons involve off-diagonal elements of Green’s functions like $D_{a\gamma }\left(x,y\right)$ and so on. Photon–phonon energy exchange via water degrees of freedom (charged bosons) is described by off-diagonal elements in our model. Entropy production by the energy exchange is represented in Equation (A218). We encounter sound–light interaction via water in our theory.

Acoustic super-radiance solution is derived in our model. We adopt an interaction Lagrangian term between transverse acoustic phonons and charged bosons $g_{aT}(\psi^{*}\left(\partial_{i}\psi \right)-$$\left(\partial_{i}\psi^{*}\right)\psi -2ie\psi^{*}\psi \left(A_{i}+a_{i}\right))Q_{aT}^{i}$ in Equation (1). This term resembles photon–charged boson interaction term in Quantum Electrodynamics (QED) with non-relativistic charged bosons. In QED theory, we can derive the super-radiance solution, cooperative spontaneous coherent light emission, for photon–charged boson degrees of freedom [102,103,104]. In a similar way to photon–charged boson system, we can derive acoustic super-radiance solution in phonon–charged boson system, namely we encounter phonon–water cooperative properties. Even in vanishing charge e=0, cooperative spontaneous decay of water molecular states from 1st excited state to the ground state occurs, and then resultant coherent sound emission emerges. Coherent sound might induce interference patterns of sound waves, resulting in pressure gradient of water systems. Interference patterns might be adopted for sound holographic information processing. Even in the tentative interference patterns in sound holography, information processing might be possible in water system in a brain. Sound holography might appear in pressure gradient in water systems, while optical holography emerges in density distribution of ionic bio-plasma or ionized water since density affects transmittance of coherent light. Hence sound holography might correlate with optical holography via water degrees of freedom. Since our model involves both photon and phonon degrees of freedom, we can investigate both sound and optical holography for information processing in a brain.

The 2nd law in thermodynamics is investigated in this paper. The kinetic entropy current for Kadanoff–Baym equations for incoherent photons, charged bosons, and phonons is introduced and the H-theorem in Hartree–Fock approximation is shown. Entropy production occurs in photon–phonon energy exchange in Equation (A218). Equation (A218) means that entropy production due to this term ceases when photon distribution $f_{T}$ is equal to phonon distributions $f_{aa,T}$ and $f_{oo,T}$, not necessarily Bose–Einstein distributions. Even in out of equilibrium states, phonon distribution tends to approach photon distribution. This means that both distributions might correlate with each other in out-of-equilibrium states. In the presence of only Equation (A218), the system never approaches thermal equilibrium state. To achieve Bose–Einstein distribution, we need contributions in Equations (A219)–(A224). Equation (A219) (Diagram (c) in Figure 1) represents photon–charged boson interaction, where charged bosons come in, photons are absorbed and charged bosons go out, and its inverse processes take place. Similarly, Equation (A220) represents photon–charged boson interaction induced by coherent sound waves, and Equations (A221) and (A222) represent phonon–charged boson interaction. Equation (A223) represents photon–charged boson interaction induced by coherent charged Bose fields. Coherent charged Bose fields collapse to incoherent charged bosons. Equation (A224) represents photon–phonon–charged boson interaction induced by coherent charged Bose fields. Even in the presence of Equations (A223) and (A224), total charge for coherent charged Bose fields and incoherent charged bosons is conserved. All the contributions for non-local terms in $\frac{i\Gamma_{2}}{2}$ in Figure 1 have been made to induce entropy production. Entropy production stops only when distributions are the Bose–Einstein distributions.

Charge and energy conservation law can be shown in spatially homogeneous system. In Section 6, we have given time-evolution equations for coherent charged Bose fields, photon fields and sound fields, and Kadanoff–Baym equations for incoherent charged bosons, photons, and phonons. Self-energy is derived by differentiating $\frac{i\Gamma_{2}}{2}$ by Green’s functions in Figure 1. Diagrams (c), (d), (e), and (f) never change the charge density for coherent charged Bose fields $-e\partial_{0}{\left|\bar{\psi }\right|}^{2}=0$ and that of incoherent charged bosons $-e\partial_{0}\int_{p}F=0$. Diagrams (g) and (h) change $-e|\bar{\psi }{|}^{2}$ or $-e\int_{p}F$. Although coherent charged Bose fields collapse to incoherent charged bosons, total charge can be shown to be conserved by similar analysis to that in Appendix in [101]. Similarly we can derive conserved total energy density in a similar way to derivation in [101,105,106].

**Figure 3:**
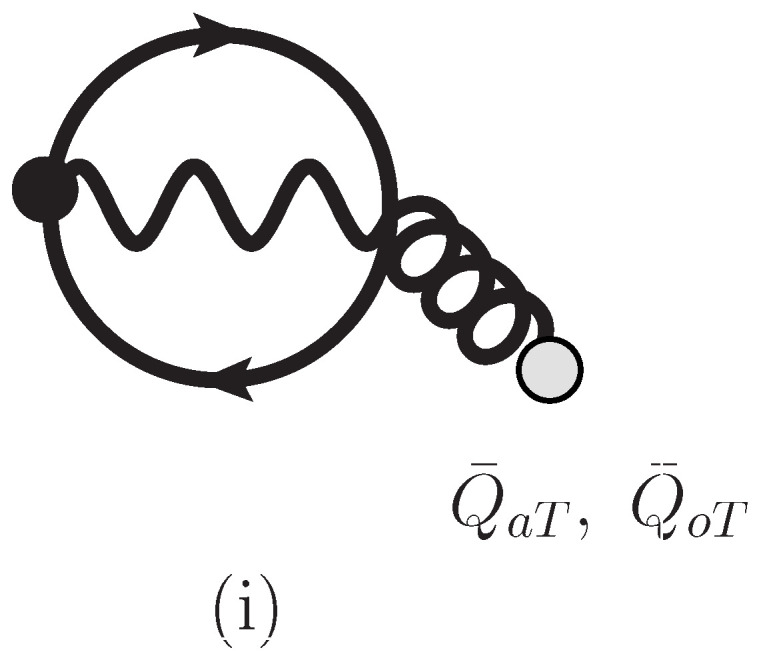
Additional 2-Particle-Irreducible loop diagram labeled by (i) in Hartree–Fock approximation. The dark circles in vertices involve covariant derivatives. The light circles in external lines represent background coherent fields ${\bar{Q}}_{aT}$, ${\bar{Q}}_{oT}$.

For Hartree–Fock approximation, we will encounter an additional $\frac{i\Gamma_{2}^{\left(i\right)}}{2}$ as shown in Figure 3. It is expressed by equation,

(33) $$\begin{array}{c} \frac{i\Gamma_{2}^{\left(i\right)}}{2}=-\frac{eg_{aT}}{m}\int_{C,z,w}{\bar{Q}}_{aT,j}\left(w\right)\left[2ie\left((\partial_{z,i}-ieA_{i}\left(z\right))\Delta \left(z,w\right)\right)\Delta \left(w,z\right)D_{T}^{ij}\left(z,w\right)\right]+\left(a\to o\right). \end{array}$$

In the presence of diagram (i) shown in this figure, we just modify self-energy for $\Sigma^{12}\left(p\right)$ and $\Pi^{12}\left(k\right)$ by,

(34) $$\begin{array}{ccc} \Sigma^{\left(c)+(d)+(i\right),12}\left(p\right) & = & -\frac{e^{2}}{4m^{2}}\int_{k}G^{12}\left(p-k\right)\\ & & \times \left[4\left({\left(p+2\tilde{Q}\right)}^{2}-\frac{{\left((p+2\tilde{Q})\cdot k\right)}^{2}}{k^{2}}\right)D_{T}^{12}\left(k\right)+\frac{{\left(k^{2}-2p\cdot k\right)}^{2}}{k^{2}}D_{L}^{12}\left(k\right)\right], \end{array}$$

(35) $$\begin{array}{ccc} \Pi_{T}^{\left(c)+(d)+(i\right),12}\left(k\right) & = & -\frac{e^{2}}{2m^{2}}\int_{p}\left({\left(p+2\tilde{Q}\right)}^{2}-\frac{{\left((p+2\tilde{Q})\cdot k\right)}^{2}}{k^{2}}\right)G^{12}\left(p+k\right)G^{21}\left(p\right), \end{array}$$

and,

(36) 12δΓ2δQ¯aTi=−2e2gaTm∫pReΞR,ij(p)F(p)+ΞF,ij(p)ReGR(p)pj+2Q˜j,

(37) 12δΓ2δQ¯oTi=−2e2goTm∫pReΞR,ij(p)F(p)+ΞF,ij(p)ReGR(p)pj+2Q˜j,

with $\tilde{Q}=m\left(g_{aT}{\bar{Q}}_{aT}+g_{oT}{\bar{Q}}_{oT}\right)$. We also set,

(38) 12δΓ2δAi=−e∫p∂ReΣR(c)+(d)+(e)+(i)(p)∂piF(p)+∂ΣF(c)+(d)+(e)+(i)(p)∂piReGR(p),

in Equation (A296). Even in the presence of diagram (i), we can prove the H-theorem since positive coefficient $\left({\left(p+2\tilde{Q}\right)}^{2}-{\left((p+2\tilde{Q})\cdot k\right)}^{2}/k^{2}\right)\geq 0$ is given, and show charge and energy conservation by using the relation (A296) for the sum of diagrams (c), (d), (e) and (i).

Application to control theory can be proposed in our model. To investigate whether our brain adopts the language of holography, we manipulate holograms composed of water media in a brain and check how our subjective experiences will change. To manipulate holograms non-invasively, we can adopt external electromagnetic fields, photons, ultrasound, and so on [42]. Adopting our model involving phonons, we obtain the control theory of non-invasive neural stimulation by external sound waves. Sound waves were found to induce changes of water conformational states in experimental study [87]. The sound perturbations with frequency 432Hz and 440Hz affect populations of ice-like (strongly bonded) water and vapor-like (less bonded) water shown in near-infrared spectroscopy with bandwidth related with symmetric and anti-symmetric stretching of water molecules. Hence we might be able to manipulate the density distributions of water molecules, namely transmittance of light or sound required in holographic memory storage. We then adopt reservoir computing or morphological computation approach [57,58,59] to control holograms, using sound. For example, to manipulate holograms of visual cortex involving water molecular distributions induced by pressure gradient, we calculate input external sound waves in scalp and impose the sound waves on scalp. Sound waves in target neocortex change pressure gradient or water molecular distributions, namely holograms of water media. Our model provides the control theory by developing QED theory with phonons in a hierarchy representing multiple layers such as scalp, skull, dura, celebrospinal fluid, and neocortex, as shown in [107].

## 8. Concluding Remarks and Perspectives

We have provided a theoretical formulation of the application of QED to the description of memory formation in the human brain where we extended previous quantum field theory models to account for the active role of water molecular states coupled with both photons and phonons in order to provide a more realistic representation for the mechanism involved. It is well-documented that the organization of water molecules in biological systems is much more orderly than in a fluid state and that this organized state of water, called Exclusion Zone (EZ) water [70] interacts in a distinct way with photons and phonons. Almost all of the water molecules in a living cells are present as EZ water [71].

Beginning with Lagrangian density, we have derived time-evolution equations for coherent fields and the Kadanoff–Baym equations for incoherent particles. Next, we have derived an acoustic super-radiance solution in cytoskeletons involving cylindrical structures in a brain like microtubules. We have shown the Kadanoff–Baym equations for incoherent charged bosons, photons, and phonons. We have also introduced a kinetic entropy current for Kadanoff–Baym equations and shown the H-theorem for self-energy in Hartree–Fock approximation. Finally, we have shown time-evolution equations in spatially homogeneous system and provided concrete forms of conserved charge and energy density. Our approach will be applied to systems composed of water coupled with sound and light, and specifically applied to a control theory of memory and consciousness physically represented by holograms of water system by external sound waves in non-invasive ultrasound neural stimulation.

## Data Availability

The data presented in this study are available on request from the corresponding author.

## Appendix A. Time-Evolution Equations

We show time-evolution equations in this appendix.

Time-evolution equations are derived by differentiating 2PI effective action in Equation (3) by expectation values of fields and Green’s functions.

For $\frac{\delta \Gamma_{2\mathrm{PI}}}{\delta {\bar{a}}^{i}}{|}_{{\bar{a}}^{i}=0}=\frac{\delta \Gamma_{2PI}}{\delta A^{i}}{|}_{{\bar{a}}^{i}=0}=0$, we can derive the relation,

(A1) $$\begin{array}{ccc} \partial^{\nu }F_{\nu i}\left[A\right] & = & -\frac{ie}{2m}[{\bar{\psi }}^{*}\left(\nabla_{i}-ieA_{i}\right)\bar{\psi }-\left(\left(\nabla_{i}+ieA_{i}\right){\bar{\psi }}^{*}\right)\bar{\psi }\\ & & +\left(\nabla_{x_{1},i}-ieA_{i}\right)\Delta^{11}\left(x_{1},x\right){|}_{x_{1}=x}-\left(\nabla_{x_{2},i}+ieA_{i}\right)\Delta^{11}\left(x,x_{2}\right){|}_{x_{2}=x}]\\ & & -\left(2eg_{aT}{\bar{Q}}_{aT,i}+2eg_{oT}{\bar{Q}}_{oT,i}\right)\left({\bar{\psi }}^{*}\bar{\psi }+\Delta^{11}\left(x,x\right)\right)-\frac{1}{2}\frac{\delta \Gamma_{2}}{\delta A^{i}}. \end{array}$$

The above equation corresponds to one of the Maxwell equations. The right-hand side represents 3-dimensional charge current including both contributions of coherent fields and those of quantum fluctuations given by Green’s functions. The term $\frac{1}{2}\frac{\delta \Gamma_{2}}{\delta A^{i}}$ includes contributions from loop expansion of 2PI diagrams required to achieve charge and energy conservation.

For $\frac{\delta \Gamma_{2\mathrm{PI}}}{\delta {\bar{\psi }}^{*}}{|}_{{\bar{a}}^{i}=0}=0$, we can derive the Gross–Pitaevskii equation,

(A2) $$\begin{array}{ccc} {}[i\frac{\partial }{\partial x^{0}}+eA_{0}+\frac{{\left(\nabla_{i}-ieA_{i}\right)}^{2}}{2m}-\frac{e^{2}D_{ii}^{11}\left(x,x\right)}{2m}-g_{aL}\left(\nabla_{i}{\bar{Q}}_{aL}^{i}\right)-g_{oL}\left(\nabla_{i}{\bar{Q}}_{oL}^{i}\right) & & \\ +2ig_{aT}{\bar{Q}}_{aT}^{i}\left(\nabla_{i}-ieA_{i}\right)+2ig_{oT}{\bar{Q}}_{oT}^{i}\left(\nabla_{i}-ieA_{i}\right) & & \\ -eg_{aT}\left(D_{\gamma aT,ii}^{11}\left(x,x\right)+D_{a\gamma T,ii}^{11}\left(x,x\right)\right)-eg_{oT}\left(D_{\gamma oT,ii}^{11}\left(x,x\right)+D_{o\gamma T,ii}^{11}\left(x,x\right)\right)]\bar{\psi }\left(x\right)+\frac{1}{2}\frac{\delta \Gamma_{2}}{\delta {\bar{\psi }}^{*}} & = & 0. \end{array}$$

This equation for charged bosons includes coupling with both photons and phonons. Their contributions are given by coherent fields $A_{\mu }$, ${\bar{Q}}_{aL}^{i}$, ${\bar{Q}}_{oL}^{i}$, ${\bar{Q}}_{aT}^{i}$ and ${\bar{Q}}_{oT}^{i}$ and their quantum fluctuations represented by Green’s functions $D_{ii}^{11}\left(x,x\right)$, $D_{\gamma aT,ii}^{11}\left(x,x\right)$, $D_{a\gamma T,ii}^{11}\left(x,x\right)$, $D_{\gamma oT,ii}^{11}\left(x,x\right)$, and $D_{o\gamma T,ii}^{11}$ for transverse parts represented by ‘*T*’ for photons labelled by γ acoustic phonons labelled by *a* and optical phonons labelled by *o*. The term $\frac{1}{2}\frac{\delta \Gamma_{2}}{\delta {\bar{\psi }}^{*}}$ includes contributions for quantum loops. For $\frac{\delta \Gamma_{2\mathrm{PI}}}{\delta \bar{\psi }}{|}_{{\bar{a}}^{i}=0}=0$, we can derive the relation,

(A3) $$\begin{array}{ccc} {}[-i\frac{\partial }{\partial x^{0}}+eA_{0}+\frac{{\left(\nabla_{i}+ieA_{i}\right)}^{2}}{2m}-\frac{e^{2}D_{ii}^{11}\left(x,x\right)}{2m}-g_{aL}\left(\nabla_{i}{\bar{Q}}_{aL}^{i}\right)-g_{oL}\left(\nabla_{i}{\bar{Q}}_{oL}^{i}\right) & & \\ -2ig_{aT}{\bar{Q}}_{aT}^{i}\left(\nabla_{i}+ieA_{i}\right)-2ig_{oT}{\bar{Q}}_{oT}^{i}\left(\nabla_{i}+ieA_{i}\right) & & \\ -eg_{aT}\left(D_{\gamma aT,ii}^{11}\left(x,x\right)+D_{a\gamma T,ii}^{11}\left(x,x\right)\right)-eg_{oT}\left(D_{\gamma oT,ii}^{11}\left(x,x\right)+D_{o\gamma T,ii}^{11}\left(x,x\right)\right)]{\bar{\psi }}^{*}\left(x\right)+\frac{1}{2}\frac{\delta \Gamma_{2}}{\delta \bar{\psi }} & = & 0. \end{array}$$

This equation is just the complex conjugate of Equation (A2).

Using Equations (A2) and (A3), we can derive the relation,

(A4) $$\begin{array}{ccc} i\frac{\partial }{\partial x^{0}}\left({\bar{\psi }}^{*}\bar{\psi }\right)+\frac{1}{2m}\nabla_{i}\left[{\bar{\psi }}^{*}\left(\nabla_{i}-ieA_{i}\right)\bar{\psi }-\left(\left(\nabla_{i}+ieA_{i}\right){\bar{\psi }}^{*}\right)\bar{\psi }\right] & & \\ +2ig_{aT}\nabla_{i}\left({\bar{Q}}_{aT}^{i}{\bar{\psi }}^{*}\bar{\psi }\right)+2ig_{oT}\nabla_{i}\left({\bar{Q}}_{oT}^{i}{\bar{\psi }}^{*}\bar{\psi }\right)+\frac{1}{2}\frac{\delta \Gamma_{2}}{\delta {\bar{\psi }}^{*}}{\bar{\psi }}^{*}-\frac{1}{2}\frac{\delta \Gamma_{2}}{\delta \bar{\psi }}\bar{\psi } & = & 0, \end{array}$$

where we have used $\nabla_{i}{\bar{Q}}_{aT}^{i}=0$ and $\nabla_{i}{\bar{Q}}_{oT}^{i}=0$. The above relation is used to prove the charge conservation law. The ${\bar{\psi }}^{*}\bar{\psi }$ represents the density of charged bosons. The terms with $\nabla_{i}$ represent the divergence of 3-dimensional current. The term $+\frac{1}{2}\frac{\delta \Gamma_{2}}{\delta {\bar{\psi }}^{*}}{\bar{\psi }}^{*}-\frac{1}{2}\frac{\delta \Gamma_{2}}{\delta \bar{\psi }}\bar{\psi }$ are written by Green’s functions $i\partial_{0}\Delta^{11}\left(x,x\right)$, so that total density ${\bar{\psi }}^{*}\bar{\psi }+\Delta^{11}\left(x,x\right)$ is conserved in time-evolution in spatially homogeneous system as shown in Appendix E.

For $\frac{\delta \Gamma_{2\mathrm{PI}}}{\delta {\bar{Q}}_{aL}^{i}}=0$ and $\frac{\delta \Gamma_{2PI}}{\delta {\bar{Q}}_{aT}^{i}}=0$, we can derive,

(A5) $$\begin{array}{ccc} \left(\partial_{0}^{2}-v_{L}^{2}\partial_{j}^{2}\right){\bar{Q}}_{aL,i}+g_{aL}\nabla_{i}\left({\bar{\psi }}^{*}\bar{\psi }+\Delta^{11}\left(x,x\right)\right)+\frac{1}{2}\frac{\delta \Gamma_{2}}{\delta {\bar{Q}}_{aL}^{i}} & = & 0, \end{array}$$

and,

(A6) $$\begin{array}{ccc} \left(\partial_{0}^{2}-v_{T}^{2}\partial_{j}^{2}\right){\bar{Q}}_{aT,i}+ig_{aT}\left({\bar{\psi }}^{*}\left(\nabla_{i}\bar{\psi }\right)-\left(\nabla_{i}{\bar{\psi }}^{*}\right)\bar{\psi }-2ieA_{i}{\bar{\psi }}^{*}\bar{\psi }\right) & & \\ +ig_{aT}\left[\left(\partial_{x_{1},i}-ieA_{i}\right)\Delta^{11}\left(x_{1},x\right){|}_{x_{1}=x}-\left(\partial_{x_{2},i}+ieA_{i}\right)\Delta^{11}\left(x,x_{2}\right){|}_{x_{2}=x}\right]+\frac{1}{2}\frac{\delta \Gamma_{2}}{\delta {\bar{Q}}_{aT}^{i}} & = & 0. \end{array}$$

We find that the longitudinal parts are coupled with the gradient of the density of charged bosons ${\bar{\psi }}^{*}\bar{\psi }+\Delta^{11}\left(x,x\right)$. In case the gradient is nonzero, coherent phonon fields ${\bar{Q}}_{aL,i}$ emerge. We also find that the transverse parts are coupled with the flow or current of charged bosons. If the current is nonzero, coherent phonon fields ${\bar{Q}}_{aT,i}$ will appear.

For $\frac{\delta \Gamma_{2\mathrm{PI}}}{\delta {\bar{Q}}_{oL}^{i}}=0$ and $\frac{\delta \Gamma_{2PI}}{\delta {\bar{Q}}_{oT}^{i}}=0$, we can derive,

(A7) $$\begin{array}{ccc} \left(\partial_{0}^{2}+\Omega_{L}^{2}\right)Q_{oL,i}+g_{oL}\nabla_{i}\left({\bar{\psi }}^{*}\bar{\psi }+\Delta^{11}\left(x,x\right)\right)+\frac{1}{2}\frac{\delta \Gamma_{2}}{\delta {\bar{Q}}_{oL}^{i}} & = & 0, \end{array}$$

and,

(A8) $$\begin{array}{ccc} \left(\partial_{0}^{2}+\Omega_{T}^{2}\right)Q_{oT,i}+ig_{oT}\left({\bar{\psi }}^{*}\left(\nabla_{i}\bar{\psi }\right)-\left(\nabla_{i}{\bar{\psi }}^{*}\right)\bar{\psi }-2ieA_{i}{\bar{\psi }}^{*}\bar{\psi }\right) & & \\ +ig_{oT}\left[\left(\partial_{x_{1},i}-ieA_{i}\right)\Delta^{11}\left(x_{1},x\right){|}_{x_{1}=x}-\left(\partial_{x_{2},i}+ieA_{i}\right)\Delta^{11}\left(x,x_{2}\right){|}_{x_{2}=x}\right]+\frac{1}{2}\frac{\delta \Gamma_{2}}{\delta {\bar{Q}}_{oT}^{i}} & = & 0. \end{array}$$

Comparing Equations (A5) and (A7), we find that the main difference is the part $\left(\partial_{0}^{2}-v_{L}^{2}\partial_{j}^{2}\right)$ and $\left(\partial_{0}^{2}+\Omega_{L}^{2}\right)$ in the 1st terms. This is originated from the difference of the dispersion relations for acoustic and optical phonons. Similarly we also find the difference in 1st terms in Equations (A6) and (A8).

Finally we can derive the Kadanoff–Baym (KB) equations [62,63,64,92] for incoherent charged bosons, phonons and photons. For $\frac{\delta \Gamma 2\mathrm{PI}}{\delta \Delta }{|}_{{\bar{a}}^{i}=0}=0$, we can derive,

(A9) $$\begin{array}{ccc} -i\Delta^{-1}+i\Delta_{0}^{-1}-i\tilde{\Sigma } & = & 0, \end{array}$$

with $i\Delta_{0}^{-1}$ given in Equation (8), Δ given in Equation (6), and $\tilde{\Sigma }=\frac{i}{2}\frac{\delta \Gamma_{2}}{\delta \Delta }$. For $\frac{\delta \Gamma 2\mathrm{PI}}{\delta D}{|}_{{\bar{a}}^{i}=0}=0$, we can derive,

(A10) $$\begin{array}{ccc} -iD^{-1}+iD_{0}^{-1}-i\Pi & = & 0, \end{array}$$

with $iD_{0}^{-1}$ given in Equation (7), D given in Equation (4), and $\Pi =i\frac{\delta \Gamma_{2}}{\delta D}$. The KB equations can trace time-evolution of quantum fluctuations with Green’s functions representing statistical and spectral properties of incoherent charged bosons, phonons and photons. We shall investigate KB equations in detail in Appendices Appendix C and Appendix E.

## Appendix B. Derivation of a Solution of Acoustic Super-Radiance

We shall derive a solution of acoustic super-radiance.

We begin with Equation (A2) rewritten by,

(A11) $$\begin{array}{c} \left(i\frac{\partial }{\partial x^{0}}+\frac{\nabla_{i}^{2}}{2m}-2ig_{aT}{\bar{Q}}_{aT,i}\nabla_{i}+\tilde{U}\left(x\right)\right)\bar{\psi }\left(x\right)=0, \end{array}$$

with potential energy $\tilde{U}\left(x\right)$. We also use Equation (A6) given by,

(A12) $$\begin{array}{ccc} \left(\partial_{0}^{2}-v_{T}^{2}\partial_{j}^{2}\right)Q_{aT,i}+ig_{aT}\left({\bar{\psi }}^{*}\nabla_{i}\bar{\psi }-\left(\nabla_{i}{\bar{\psi }}^{*}\right)\bar{\psi }\right) & = & 0. \end{array}$$

Next we shall expand $\bar{\psi }\left(x\right)$ as,

(A13) $$\begin{array}{c} \bar{\psi }\left(x\right)=\sum_{n}b_{n}\left(x^{0}\right)\phi_{n}\left(x\right), \end{array}$$

where $\phi_{n}\left(x\right)$’s are normalized eigenstates satisfying the relation,

(A14) $$\begin{array}{c} \left(-\frac{\nabla_{i}^{2}}{2m}+\tilde{U}\left(x\right)\right){\bar{\phi }}_{n}\left(x\right)=E_{n}\phi_{n}\left(x\right), \end{array}$$

with eigenvalues $E_{n}$. The Equation (A11) is written by,

(A15) $$\begin{array}{ccc} i\frac{\partial }{\partial x^{0}}\left(\sum_{n}b_{n}\left(x^{0}\right)\phi_{n}\left(x\right)\right) & = & \left(-\frac{\nabla_{i}^{2}}{2m}+\tilde{U}\right)\left(\sum_{n}b_{n}\left(x^{0}\right)\phi_{n}\left(x\right)\right)+2ig_{aT}{\bar{Q}}_{aT,i}\sum_{n}b_{n}\left(x^{0}\right)\nabla_{i}\phi_{n}\left(x\right). \end{array}$$

We shall use,

(A16) $$\begin{array}{c} b_{n}\left(x^{0}\right)=e^{-iE_{n}x^{0}}\chi_{n}\left(x^{0}\right). \end{array}$$

The normalization condition is written as $\int_{x}|\bar{\psi }{|}^{2}=\sum_{n}|b_{n}\left(x^{0}\right){|}^{2}=\sum_{n}{\left|\chi_{n}\left(x^{0}\right)\right|}^{2}=N$ with the number of water molecules *N*. We shall consider the case $|\nabla_{3}\phi_{n}\left|\gg \right|\nabla_{1}\phi_{n}\left|,\right|\nabla_{2}\phi_{n}|$ and $Q_{aT,1},Q_{aT,2}≃0$. The $x^{3}$ axis represents the cylindrical axis of microtubules. We show coherent sound emission with circular waves in $x^{1}$-$x^{2}$ plane in this section.

Here we use two-energy level approximation for energy eigenvalues $E_{0}$ for the ground state and $E_{1}$ for 1st excited state. We expand ${\bar{Q}}_{aT,3}$ as,

(A17) $$\begin{array}{c} {\bar{Q}}_{aT,3}=\int_{k^{1},k^{2}}\frac{1}{2}\left(q_{3,k}e^{-i(\omega_{k}x^{0}-k\cdot x)}+q_{3,k}^{*}e^{i(\omega_{k}x^{0}-k\cdot x)}\right), \end{array}$$

with $\int_{k^{1},k^{2}}=\int \frac{dk^{1}dk^{2}}{{\left(2\pi \right)}^{2}}$ and $\omega_{k}=\left|k\right|v_{T}$. Substituting ${\bar{Q}}_{aT,3}$ in Equation (A15) by Equation (A17), multiplying $\phi_{0}^{*}\left(x\right)$, integrating by $\int_{x}=\int d^{3}x$ and using Equation (A16), we can derive time-evolution equation for $\chi_{0}\left(x^{0}\right)$ as,

(A18) $$\begin{array}{ccc} i\frac{\partial \chi_{0}}{\partial x^{0}} & = & 2g_{aT}\int_{x}\int_{k^{1},k^{2}}\frac{1}{2}\left(q_{3,k}e^{-i(\omega_{k}x^{0}-k\cdot x)}+q_{3,k}^{*}e^{i(\omega_{k}x^{0}-k\cdot x)}\right)\\ & & \times \left[\phi_{0}^{*}i\left(\nabla_{3}\phi_{1}\right)\chi_{1}e^{-i\bar{\Omega }x^{0}}+\phi_{0}^{*}i\left(\nabla_{3}\phi_{0}\right)\chi_{0}\right], \end{array}$$

with energy difference between 1st excited state and the ground state $\bar{\Omega }=E_{1}-E_{0}$. Similarly, multiplying $\phi_{1}^{*}\left(x\right)$, integrating by $\int_{x}$ and using Equation (A16), we derive time-evolution equation for $\chi_{0}\left(x^{0}\right)$ as,

(A19) $$\begin{array}{ccc} i\frac{\partial \chi_{1}}{\partial x^{0}} & = & 2g_{aT}\int_{x}\int_{k^{1},k^{2}}\frac{1}{2}\left(q_{3,k}e^{-i(\omega_{k}x^{0}-k\cdot x)}+q_{3,k}^{*}e^{i(\omega_{k}x^{0}-k\cdot x)}\right)\\ & & \times \left[\phi_{1}^{*}i\left(\nabla_{3}\phi_{0}\right)\chi_{0}e^{i\bar{\Omega }x^{0}}+\phi_{1}^{*}i\left(\nabla_{3}\phi_{1}\right)\chi_{1}\right]. \end{array}$$

We shall extract only the resonant mode ($\omega_{k}=\bar{\Omega }$) representing rotating-wave approximation in Equations (A18) and (A19). We then rewrite Equations (A18) and (A19) by,

(A20) $$\begin{array}{ccc} i\frac{\partial \chi_{0}}{\partial x^{0}} & = & g_{aT}\int_{k^{1},k^{2};\omega_{k}=\bar{\Omega }}q_{3,k}^{*}\int_{x}e^{-ik\cdot x}\left(\phi_{0}^{*}i\nabla_{3}\phi_{1}\right)\chi_{1}, \end{array}$$

(A21) $$\begin{array}{ccc} i\frac{\partial \chi_{1}}{\partial x^{0}} & = & g_{aT}\int_{k^{1},k^{2};\omega_{k}=\bar{\Omega }}q_{3,k}\int_{x}e^{ik\cdot x}\left(\phi_{1}^{*}i\nabla_{3}\phi_{0}\right)\chi_{0}. \end{array}$$

Next using the relation,

(A22) $$\begin{array}{ccc} & & \int_{x}e^{-il\cdot x}\left(\partial_{0}^{2}-v_{T}^{2}\partial_{j}^{2}\right)\int_{k^{1},k^{2}}\frac{1}{2}\left(q_{3,k}e^{-i(\omega_{k}x^{0}-k\cdot x)}+q_{3,k}^{*}e^{i(\omega_{k}x^{0}-k\cdot x)}\right)\\ & = & \int_{x}e^{-il\cdot x}\int_{k^{1},k^{2}}\left(-\omega_{k}^{2}+v_{T}^{2}\left(k_{1}^{2}+k_{2}^{2}\right)\right)\frac{1}{2}q_{3,k}e^{-i(\omega_{k}x^{0}-k\cdot x)}\\ & & +\int_{x}e^{-il\cdot x}\int_{k^{1},k^{2}}\left(-i\omega_{k}\partial_{0}q_{3,k}-iv_{T}^{2}\left(k^{1}\partial_{1}q_{3,k}+k^{2}\partial_{2}q_{3,k}\right)\right)e^{-i(\omega_{k}x^{0}-k\cdot x)}+\mathrm{other}\;\mathrm{terms}\\ & = & \left(-i\omega_{l}\partial_{0}q_{3,l}-iv_{T}^{2}\left(l^{1}\partial_{1}q_{3,l}+l^{2}\partial_{2}q_{3,l}\right)\right)L_{3}e^{-i\omega_{l}x^{0}}+\mathrm{other}\;\mathrm{terms}, \end{array}$$

where momentum l represents $l=(l^{1},l^{2},0)$, and $L_{3}$ is the size of the system in $x^{3}$ direction, we can rewrite Equation (A12) by,

(A23) $$\begin{array}{cc} & \left(-i\omega_{l}\partial_{0}q_{3,l}-iv_{T}^{2}\left(l^{1}\partial_{1}q_{3,l}+l^{2}\partial_{2}q_{3,l}\right)\right)L_{3}e^{-i\omega_{l}x^{0}}+\mathrm{other}\;\mathrm{terms}\\ = & -ig_{aT}\int_{x}e^{-il\cdot x}[e^{i\bar{\Omega }x^{0}}\chi_{1}^{*}\chi_{0}\left(\phi_{1}^{*}\nabla_{3}\phi_{0}\right)+e^{-i\bar{\Omega }x^{0}}\chi_{0}^{*}\chi_{1}\left(\phi_{0}^{*}\nabla_{3}\phi_{1}\right)\\ & -e^{-i\bar{\Omega }x^{0}}\chi_{0}^{*}\chi_{1}\left(\phi_{1}\nabla_{3}\phi_{0}^{*}\right)-e^{i\bar{\Omega }x^{0}}\chi_{0}\chi_{1}^{*}\left(\phi_{0}^{*}\nabla_{3}\phi_{1}^{*}\right)]. \end{array}$$

(Here we consider the case $|\omega_{k}q_{3,k}\left|\gg \right|\partial_{0}q_{3,k}|$.) Taking only the resonant mode, we arrive at,

(A24) $$\begin{array}{c} \left(\frac{\partial q_{3,k}}{\partial x^{0}}+\frac{v_{T}^{2}}{\omega_{k}}\left(k^{1}\frac{\partial q_{3,k}}{\partial x^{1}}+k^{2}\frac{\partial q_{3,k}}{\partial x^{2}}\right)\right)=-i\frac{2}{\omega_{k}L_{3}}\chi_{0}^{*}\chi_{1}\int_{x}e^{-ik\cdot x}\phi_{0}^{*}ig_{aT}\nabla_{3}\phi_{1}. \end{array}$$

Next we adopt dipole approximation $e^{ik\cdot x}≃1$ where wavelength of sound is larger than the size of the system like a microtubule. We then rewrite Equations (A20), (A21), and (A24) by,

(A25) $$\begin{array}{ccc} i\frac{\partial \chi_{0}}{\partial x^{0}} & = & g_{aT}\int_{k^{1},k^{2};\omega_{k}=\bar{\Omega }}q_{3,k}^{*}\int_{x}\left(\phi_{0}^{*}i\nabla_{3}\phi_{1}\right)\chi_{1}, \end{array}$$

(A26) $$\begin{array}{ccc} i\frac{\partial \chi_{1}}{\partial x^{0}} & = & g_{aT}\int_{k^{1},k^{2};\omega_{k}=\bar{\Omega }}q_{3,k}\int_{x}\left(\phi_{1}^{*}i\nabla_{3}\phi_{0}\right)\chi_{0}, \end{array}$$

and,

(A27) $$\begin{array}{c} \left(\frac{\partial q_{3,k}}{\partial x^{0}}+\frac{v_{T}^{2}}{\omega_{k}}\left(k^{1}\frac{\partial q_{3,k}}{\partial x^{1}}+k^{2}\frac{\partial q_{3,k}}{\partial x^{2}}\right)\right)=-i\frac{2g_{aT}}{\omega_{k}L_{3}}\chi_{0}^{*}\chi_{1}\int_{x}\phi_{0}^{*}i\nabla_{3}\phi_{1}. \end{array}$$

We shall define,

(A28) $$\begin{array}{ccc} J_{n_{1}n_{2}} & \equiv & \int_{x}\phi_{n_{1}}^{*}\frac{i\nabla_{3}}{m}\phi_{n_{2}}, \end{array}$$

(A29) $$\begin{array}{ccc} Q & \equiv & \int_{k^{1},k^{2};\omega_{k}=\bar{\Omega }}q_{3,k}, \end{array}$$

and use the relation,

(A30) $$\begin{array}{ccc} \int_{k^{1},k^{2};\omega_{k}=\bar{\Omega }} & = & {\bar{\Omega }}^{2}2\pi \int_{0}^{\infty }ydy\delta \left(y^{2}-\frac{1}{v_{T}^{2}}\right)\frac{1}{{\left(2\pi \right)}^{2}}\\ & = & \frac{{\bar{\Omega }}^{2}}{4\pi }, \end{array}$$

with $y=\frac{1}{\bar{\Omega }}\sqrt{{\left(k^{1}\right)}^{2}+{\left(k^{2}\right)}^{2}}$. From Equations (A25)–(A27), we then derive the relations,

(A31) $$\begin{array}{ccc} \frac{\partial \chi_{0}}{\partial x^{0}} & = & -img_{aT}Q^{*}J_{01}\chi_{1}, \end{array}$$

(A32) $$\begin{array}{ccc} \frac{\partial \chi_{1}}{\partial x^{0}} & = & -img_{aT}QJ_{10}\chi_{0}, \end{array}$$

(A33) $$\begin{array}{ccc} \;\;\frac{\partial Q}{\partial x^{0}} & = & -\frac{i\bar{\Omega }}{2\pi L_{3}}mg_{aT}J_{01}\chi_{0}^{*}\chi_{1}. \end{array}$$

Here the $J_{01}$ and $J_{10}$ are related with transition dipole moment since Equation (A28) is the matrix element of the momentum $-i\nabla_{3}∼d{\tilde{X}}_{3}/dx^{0}∼\left[X_{3},H\right]$ with position ${\tilde{X}}_{3}$ and Hamiltonian H.

Next we set Q→−iQ and assume real Q and $J_{01}=J_{10}$, we arrive at,

(A34) $$\begin{array}{ccc} \frac{\partial \chi_{0}}{\partial x^{0}} & = & gQ\chi_{1}, \end{array}$$

(A35) $$\begin{array}{ccc} \;\frac{\partial \chi_{1}}{\partial x^{0}} & = & -gQ\chi_{0}, \end{array}$$

(A36) $$\begin{array}{ccc} \;\;\frac{\partial Q}{\partial x^{0}} & = & \frac{\bar{\Omega }}{2\pi L_{3}}g\chi_{0}^{*}\chi_{1}, \end{array}$$

with $g\equiv mg_{aT}J_{01}$. We can derive density and energy conservation law as,

(A37) $$\begin{array}{ccc} \;\;\;\;\;\;\frac{\partial }{\partial x^{0}}\left(|\chi_{0}{|}^{2}+{\left|\chi_{1}\right|}^{2}\right) & = & 0, \end{array}$$

(A38) $$\begin{array}{ccc} \frac{\partial }{\partial x^{0}}\left(\frac{1}{2}Q^{2}+\frac{\bar{\Omega }}{8\pi L_{3}}\left(\right|\chi_{1}{|}^{2}-|\chi_{0}{|}^{2})\right) & = & 0. \end{array}$$

Setting real $R\equiv 2\chi_{0}^{*}\chi_{1}$ and population difference $Z\equiv |\chi_{1}{|}^{2}-{\left|\chi_{0}\right|}^{2}$, *R* and *Z* satisfy,

(A39) $$\begin{array}{ccc} \partial_{0}Z & = & -2gQR, \end{array}$$

(A40) $$\begin{array}{ccc} \partial_{0}R & = & 2gQZ, \end{array}$$

(A41) $$\begin{array}{ccc} \partial_{0}Q & = & \frac{g\bar{\Omega }}{4\pi L_{3}}R. \end{array}$$

We can then use the conserved quantitiy,

(A42) $$\begin{array}{c} N^{2}=Z^{2}+R^{2}, \end{array}$$

where *N* is the number of water molecules in particular conformational states. We shall then set $Z=N\cos\theta (x^{0})$ and $R=N\sin\theta (x^{0})$. We derive the following relations from the above equations,

(A43) $$\begin{array}{ccc} \partial_{0}\theta & = & 2gQ, \end{array}$$

(A44) $$\begin{array}{ccc} \;\;\;\;\partial_{0}Q & = & \frac{g\bar{\Omega }}{4\pi L_{3}}N\sin\theta . \end{array}$$

From Equation (A43), we can derive,

(A45) $$\begin{array}{c} \theta \left(x^{0}\right)=\theta_{0}+\int_{t_{0}}^{x^{0}}dt2gQ. \end{array}$$

Moreover we rewrite Equation (A44) as,

(A46) $$\begin{array}{c} \partial_{0}Q+L_{3}^{-1}Q=\frac{g\bar{\Omega }N}{4\pi L_{3}}\sin\theta , \end{array}$$

where $L_{3}^{-1}Q$ represents the term for emission of sound. We consider the case $L_{3}^{-1}\gg \partial_{0}$. Using Equation (A43), the above equation is rewritten by,

(A47) $$\begin{array}{c} \partial_{0}\theta =\frac{g^{2}\bar{\Omega }N}{2\pi }\sin\theta . \end{array}$$

The solution of the above equation is,

(A48) $$\begin{array}{c} \theta \left(x^{0}\right)=2\tan^{-1}\left(\exp\left(\frac{g^{2}\bar{\Omega }N}{2\pi }x^{0}\right)\tan\frac{\theta_{0}}{2}\right). \end{array}$$

Then we finally arrive at,

(A49) $$\begin{array}{c} Q=\frac{1}{2g}\partial_{0}\theta =\frac{g\bar{\Omega }N}{4\pi }{\left[\cosh\left(\frac{x^{0}-\tau_{0}}{\tau_{R}}\right)\right]}^{-1}, \end{array}$$

where $\tau_{R}$ represents,

(A50) $$\begin{array}{c} \tau_{R}=\frac{2\pi }{g^{2}\bar{\Omega }N}\propto \frac{1}{N}, \end{array}$$

and $\tau_{0}=-\tau_{R}\ln\tan\frac{\theta_{0}}{2}$. Since *N* water molecules decay in 1/N time scale cooperatively, energy of coherent sound is the order of $N^{2}$ instantly. We have derived an acoustic super-radiance solution in our model.

## Appendix C. Kadanoff–Baym Equations

In this section, we write Kadanoff–Baym equations for incoherent charged bosons, phonons and photons.

The $\frac{i\Gamma_{2}}{2}$ is given in Figure 1. Local terms are expressed by,

(A51) $$\begin{array}{c} \frac{i\Gamma_{2}^{\left(a\right)}}{2}=-i\frac{e^{2}}{2m}\int_{C}d^{4}x\Delta \left(x,x\right)D_{ii}\left(x,x\right), \end{array}$$

and,

(A52) $$\begin{array}{c} \frac{i\Gamma_{2}^{\left(b\right)}}{2}=-ie\int_{C}d^{4}x\Delta \left(x,x\right)\left[g_{aT}\left(D_{a\gamma T,ii}\left(x,x\right)+D_{\gamma aT,ii}\left(x,x\right)\right)+g_{oT}\left(D_{o\gamma T,ii}\left(x,x\right)+D_{\gamma oT,ii}\left(x,x\right)\right)\right]. \end{array}$$

The $\frac{i\Gamma_{2}^{\left(a\right)}}{2}$ provides local self-energy of mass shift of photons and charged bosons. On the other hand, $\frac{i\Gamma_{2}^{\left(b\right)}}{2}$ provides the exchange between photons and phonons with density of incoherent charged bosons Δ(x,x). Non-local terms are expressed by,

(A53) $$\begin{array}{ccc} \frac{i\Gamma_{2}^{\left(c\right)}}{2} & = & -\frac{e^{2}}{8m^{2}}\int_{C,z,w}[-4\left((\nabla_{z,i}-ieA_{i}\left(z\right))\Delta \left(z,w\right)\right)\left((\nabla_{w,j}-ieA_{j}\left(w\right))\Delta \left(w,z\right)\right)D_{ij}\left(z,w\right)\\ & & -2\left((\nabla_{z,i}-ieA_{i}\left(z\right))\Delta \left(z,w\right)\right)\Delta \left(w,z\right)\partial_{w,j}D_{ij}\left(z,w\right)\\ & & -2\Delta \left(z,w\right)\left((\nabla_{w,j}-ieA_{j}\left(w\right))\Delta \left(w,z\right)\right)\partial_{z,i}D_{ij}\left(z,w\right)\\ & & -\Delta \left(z,w\right)\Delta \left(w,z\right)\partial_{z,i}\partial_{w,j}D_{ij}\left(z,w\right)], \end{array}$$

(A54) $$\begin{array}{ccc} \frac{i\Gamma_{2}^{\left(d\right)}}{2} & = & -2e^{2}\int_{C,z,w}\left(g_{aT}{\bar{Q}}_{aT,i}\left(z\right)+g_{oT}{\bar{Q}}_{oT,i}\left(z\right)\right)\left(g_{aT}{\bar{Q}}_{aT,j}\left(w\right)+g_{oT}{\bar{Q}}_{oT,j}\left(w\right)\right)\\ & & \times \Delta \left(z,w\right)\Delta \left(w,z\right)D_{T,ij}\left(z,w\right), \end{array}$$

(A55) $$\begin{array}{ccc} \frac{i\Gamma_{2}^{\left(e\right)}}{2} & = & -\frac{g_{aT}^{2}}{2}\int_{C,z,w}[-\left((\nabla_{z,i}-ieA_{T,i}\left(z\right))\Delta \left(z,w\right)\right)\left((\nabla_{w,j}-ieA_{T,j}\left(w\right))\Delta \left(w,z\right)\right)\\ & & +\left(\left(\nabla_{z,i}-ieA_{T,i}\left(z\right)\right)\left(\nabla_{w,j}+ieA_{T,j}\left(w\right)\right)\Delta \left(z,w\right)\right)\Delta \left(w,z\right)\\ & & +\Delta \left(z,w\right)\left(\left(\nabla_{z,i}+ieA_{T,i}\left(z\right)\right)\left(\nabla_{w,j}-ieA_{T,j}\left(w\right)\right)\Delta \left(w,z\right)\right)\\ & & -\left((\nabla_{w,j}+ieA_{T,j}\left(w\right))\Delta \left(z,w\right)\right)\left((\nabla_{z,i}+ieA_{T,i}\left(z\right))\Delta \left(w,z\right)\right)]D_{aa,T,ij}\left(z,w\right)\\ & & +(a\to o), \end{array}$$

(A56) $$\begin{array}{ccc} \;\;\frac{i\Gamma_{2}^{\left(f\right)}}{2} & = & -\frac{g_{aL}^{2}}{2}\int_{C,z,w}\Delta \left(z,w\right)\Delta \left(w,z\right)\partial_{z,i}\partial_{w,j}D_{aa,L,ij}\left(z,w\right)+\left(a\to o\right), \end{array}$$

(A57) $$\begin{array}{ccc} \frac{i\Gamma_{2}^{\left(g\right)}}{2} & = & -\frac{e^{4}}{2m^{2}}\int_{C,z,w}{\bar{\psi }}^{*}\left(z\right)\bar{\psi }\left(w\right)\Delta \left(z,w\right)D_{ij}\left(z,w\right)D_{ji}\left(w,z\right), \end{array}$$

(A58) $$\begin{array}{ccc} \;\;\;\frac{i\Gamma_{2}^{\left(h\right)}}{2} & = & -4{\left(eg_{aT}\right)}^{2}\int_{C,z,w}{\bar{\psi }}^{*}\left(z\right)\bar{\psi }\left(w\right)\Delta \left(z,w\right)D_{T,ij}\left(z,w\right)D_{aa,T,ji}\left(w,z\right)\\ & & -4{\left(eg_{oT}\right)}^{2}\int_{C,z,w}{\bar{\psi }}^{*}\left(z\right)\bar{\psi }\left(w\right)\Delta \left(z,w\right)D_{T,ij}\left(z,w\right)D_{oo,T,ji}\left(w,z\right), \end{array}$$

where (a→o) represents changing subscript ‘acouctic’ *a* to ‘optical’ *o* in the previous term. The $\frac{i\Gamma_{2}^{\left(c\right)}}{2}$ represents charged bosons and photons, where incoherent charged bosons come in, absorb incoherent photons and change their momenta, and its inverse processes. $\frac{i\Gamma_{2}^{\left(d\right)}}{2}$ represents the contributions where incoherent charged bosons are coupled with incoherent photons and transverse coherent sound fields ${\bar{Q}}_{aT,i}$ and ${\bar{Q}}_{oT,i}$. The $\frac{i\Gamma_{2}^{\left(e\right)}}{2}$ represents the process between incoherent charged bosons and transverse incoherent phonons. The $\frac{i\Gamma_{2}^{\left(f\right)}}{2}$ represents the process where incoherent charged bosons absorb longitudinal incoherent phonons and change their momenta, and its inverse processes. The $\frac{i\Gamma_{2}^{\left(g\right)}}{2}$ provides the interaction where coherent charged boson fields decay to incoherent charged bosons with incoming and outgoing photons. The $\frac{i\Gamma_{2}^{\left(h\right)}}{2}$ represents the process where coherent charged boson fields decay to incoherent charged bosons with photons and phonons. These processes can be seen by cutting symmetrical nonlocal diagrams in Figure 1 with the vertical line.

Next we shall investigate the self-energy. Using $\Pi \left(x,y\right)=\frac{\delta i\Gamma_{2}}{\delta D(y,x)}$, we write,

(A59) $$\begin{array}{ccc} \Pi (x,y) & = & -i\delta_{C}\left(x-y\right)\Pi_{\mathrm{loc}}\left(x\right)+\Pi_{\mathrm{nonl}}\left(x,y\right), \end{array}$$

with,

(A60) $$\begin{array}{ccc} \Pi_{\mathrm{loc}}\left(x\right) & = & \left(\begin{array}{ccc} \frac{e^{2}\Delta^{11}\left(x,x\right)}{m}\delta_{ij} & 2eg_{aT}\Delta^{11}\left(x,x\right)P_{T,ij} & 2eg_{oT}\Delta^{11}\left(x,x\right)P_{T,ij}\\ 2eg_{aT}\Delta^{11}\left(x,x\right)P_{T,ij} & 0 & 0\\ 2eg_{oT}\Delta^{11}\left(x,x\right)P_{T,ij} & 0 & 0 \end{array}\right), \end{array}$$

with $P_{T,ij}=\left(\delta_{ij}-\partial_{i}\partial_{j}/\partial_{k}^{2}\right)$, and,

(A61) $$\begin{array}{c} \Pi_{\mathrm{nonl}}\left(x,y\right)=\mathrm{diag}\left(\Pi_{ij}\left(x,y\right),\Pi_{aa,ij}\left(x,y\right),\Pi_{oo,ij}\left(x,y\right)\right), \end{array}$$

where we investigate diagonal elements for non-local self-energy since off-diagonal elements are higher order in the coupling expansion of *e*, $g_{aT}$, and $g_{oT}$.

Next we shall investigate Equation (A10) in Appendix A or,

(A62) $$\begin{array}{c} \left(iD_{0}^{-1}-i\Pi \right)D=iI, \end{array}$$

where I is a unit matrix. Taking the 11 component (or γγ component) of the above equation with Equations (7), (A61) and (A245) is written by,

(A63) $$\begin{array}{ccc} & & \left[-\left(\partial_{x}^{2}+\frac{e^{2}\left(|\bar{\psi }{\left(x\right)|}^{2}+\Delta^{11}\left(x,x\right)\right)}{m}\right)\delta_{ij}-i\Pi_{\mathrm{nonl},ij}\right]D_{jk}-2eg_{aT}\left(|\bar{\psi }{|}^{2}+\Delta^{11}\right)P_{T,ij}D_{a\gamma ,jk}\\ & & -2eg_{oT}\left(|\bar{\psi }{|}^{2}+\Delta^{11}\right)P_{T,ij}D_{o\gamma ,jk}=i\delta_{ik}\delta_{C}\left(x-y\right). \end{array}$$

We shall take the aγ component in Equation (A62). We then derive the following relation,

(A64) $$\begin{array}{c} -2eg_{aT}\left(\right|\bar{\psi }{|}^{2}+\Delta^{11})P_{T,ij}D_{jk}+\left[-\left(\partial_{0}^{2}\delta_{ij}-v_{2,ij}\partial_{l}^{2}\right)-i\Pi_{\mathrm{nonl},aa,ij}\right]D_{a\gamma ,jk}=0. \end{array}$$

Introducing the $d_{aa,ij}$ satisfying,

(A65) $$\begin{array}{c} -\left(\partial_{0}^{2}\delta_{ik}-v_{2,jk}\partial_{l}^{2}\right)d_{aa,kj}-i\Pi_{\mathrm{nonl},aa,ik}d_{aa,kj}=i\delta_{ij}\delta_{C}, \end{array}$$

we find

(A66) $$\begin{array}{c} -2eg_{aT}\left(|\bar{\psi }{|}^{2}+\Delta^{11}\right)P_{T,ij}D_{jk}+id_{aa,ij}^{-1}D_{a\gamma ,jk}=0. \end{array}$$

Then we find,

(A67) $$\begin{array}{c} D_{a\gamma ,T,ik}=\frac{2}{i}d_{aa,T,ij}eg_{aT}\left(|\bar{\psi }{|}^{2}+\Delta^{11}\right)P_{T,jl}D_{T,lk}, \end{array}$$

with the subscript *T* for transverse component. Similarly introducing $d_{oo,ij}$ satisfying,

(A68) $$\begin{array}{c} -\left(\partial_{0}^{2}\delta_{ik}+\Omega_{2,ik}\right)d_{oo,kj}-i\Pi_{\mathrm{nonl},oo,ik}d_{oo,kj}=i\delta_{C}\delta_{ij}, \end{array}$$

we find,

(A69) $$\begin{array}{c} D_{o\gamma ,T,ik}=\frac{2}{i}d_{oo,T,ij}eg_{oT}\left(|\bar{\psi }{|}^{2}+\Delta^{11}\right)P_{T,jl}D_{T,lk}. \end{array}$$

Using Equations (A67) and (A69), Equation (A63) is rewritten by,

(A70) $$\begin{array}{ccc} \left[-\left(\partial^{2}+\frac{e^{2}\left(|\bar{\psi }{|}^{2}+\Delta \right)}{m}\right)-i\Pi_{\mathrm{nonl},L}\right]D_{L} & = & i\delta_{C}, \end{array}$$

and,

(A71) $$\begin{array}{c} -\left(\partial^{2}+\frac{e^{2}\left(|\bar{\psi }{|}^{2}+\Delta \right)}{m}-i\Pi_{\mathrm{nonl},T}\right)D_{T}+4ieg_{aT}\left(|\bar{\psi }{|}^{2}+\Delta \right)d_{aa,T}eg_{aT}\left(|\bar{\psi }{|}^{2}+\Delta \right)D_{T}\\ +4ieg_{oT}\left(|\bar{\psi }{|}^{2}+\Delta \right)d_{oo,T}eg_{oT}\left(|\bar{\psi }{|}^{2}+\Delta \right)D_{T}=i\delta_{C}, \end{array}$$

where we have used the projection $P_{T,ij}$ with subscript ‘*T*’ (transverse) and $P_{L,ij}=\delta_{ij}-P_{T,ij}$ with subscript ‘*L*’ (longitudinal) in Equation (A64).

Next we shall investigate,

(A72) $$\begin{array}{c} D\left(iD_{0}^{-1}-i\Pi \right)=iI. \end{array}$$

Taking γγ component in the above equation with Equations (7) and, we can derive

(A73) $$\begin{array}{ccc} D_{ij}\left[-\left(\partial^{2}+\frac{e^{2}\left(|\bar{\psi }{|}^{2}+\Delta \right)}{m}\right)\delta_{jk}-i\Pi_{jk}\right]-D_{\gamma a,ij}2eg_{aT}\left(|\bar{\psi }{|}^{2}+\Delta \right)P_{T,jk} & & \\ -D_{\gamma o,ij}2eg_{oT}\left(|\bar{\psi }{|}^{2}+\Delta \right)P_{T,jk} & = & i\delta_{ik}\delta_{C}. \end{array}$$

Taking γa component in Equation (A72) with Equations (7), (A61) and (A245), we find,

(A74) $$\begin{array}{c} -D_{ij}2eg_{aT}\left(|\bar{\psi }{|}^{2}+\Delta \right)P_{T,jk}+D_{\gamma a,ij}\left(-\left(\delta_{jk}\partial_{0}^{2}-v_{2,jk}\partial_{l}^{2}\right)-i\Pi_{\mathrm{nonl},aa,jk}\right)=0. \end{array}$$

Using $d_{aa,ij}$ satisfying Equation (A65), we find,

(A75) $$\begin{array}{c} D_{\gamma a,ij}=\frac{2}{i}D_{ik}eg_{aT}\left(\right|\bar{\psi }{|}^{2}+\Delta )P_{T,kl}d_{aa,lj}. \end{array}$$

Similarly taking γo component in Equation (A72) and using Equation (A68), we find,

(A76) $$\begin{array}{c} D_{\gamma o,ij}=\frac{2}{i}D_{ik}eg_{oT}\left(\right|\bar{\psi }{|}^{2}+\Delta )P_{T,kl}d_{oo,lj}. \end{array}$$

Using the projection $P_{T,ij}$ with subscript ‘*T*’ (transverse) and $P_{L,ij}=\delta_{ij}-P_{T,ij}$ with subscript ‘*L*’ (longitudinal) in Equation (A74) with Equations (A75) and (A76), we can derive,

(A77) $$\begin{array}{c} D_{L}\left[-\left(\partial^{2}+\frac{e^{2}\left(|\bar{\psi }{|}^{2}+\Delta \right)}{m}\right)-i\Pi_{L}\right]=i\delta_{C}, \end{array}$$

and,

(A78) $$\begin{array}{ccc} D_{T}\left[-\left(\partial^{2}+\frac{e^{2}\left(|\bar{\psi }{|}^{2}+\Delta \right)}{m}\right)-i\Pi_{T}\right]+4iD_{T}eg_{aT}\left(|\bar{\psi }{|}^{2}+\Delta \right)d_{aa,T}eg_{aT}\left(|\bar{\psi }{|}^{2}+\Delta \right) & & \\ +4iD_{T}eg_{oT}\left(|\bar{\psi }{|}^{2}+\Delta \right)d_{oo,T}eg_{oT}\left(|\bar{\psi }{|}^{2}+\Delta \right) & = & i\delta_{C}. \end{array}$$

Next we shall take aa component in Equation (A62). We then derive,

(A79) $$\begin{array}{ccc} -2eg_{aT}\left(|\bar{\psi }{|}^{2}+\Delta \right)P_{T,ij}D_{\gamma a,jk}+\left(-\left(\partial_{0}^{2}\delta_{ij}-v_{2,ij}\partial_{l}^{2}\right)-i\Pi_{\mathrm{nonl},aa,ij}\right)D_{aa,jk} & = & i\delta_{ik}\delta_{C}. \end{array}$$

Using Equation (A75), and taking the projection on transverse and longitudinal parts, we find,

(A80) $$\begin{array}{c} \left[-\left(\partial_{0}^{2}-v_{T}\partial_{l}^{2}\right)-i\Pi_{\mathrm{nonl},aa,T}\right]D_{aa,T}+4ieg_{aT}\left(|\bar{\psi }{|}^{2}+\Delta \right)D_{T}eg_{aT}\left(|\bar{\psi }{|}^{2}+\Delta \right)d_{aa,T}=i\delta_{C}, \end{array}$$

and,

(A81) $$\begin{array}{ccc} \left[-\left(\partial_{0}^{2}-v_{L}\partial_{l}^{2}\right)-i\Pi_{\mathrm{nonl},aa,L}\right]D_{aa,L} & = & i\delta_{C}. \end{array}$$

Similarly the oo component in Equation (A62) is written by,

(A82) $$\begin{array}{c} \left[-\left(\partial_{0}^{2}+\Omega_{T}^{2}\right)-i\Pi_{\mathrm{nonl},oo,T}\right]D_{oo,T}+4ieg_{oT}\left(|\bar{\psi }{|}^{2}+\Delta \right)D_{T}eg_{oT}\left(|\bar{\psi }{|}^{2}+\Delta \right)d_{oo,T}=i\delta_{C}, \end{array}$$

and,

(A83) $$\begin{array}{ccc} \left[-\left(\partial_{0}^{2}+\Omega_{L}^{2}\right)-i\Pi_{\mathrm{nonl},oo,L}\right]D_{oo,L} & = & i\delta_{C}. \end{array}$$

Taking the aa component in Equation (A72) with Equations (A61) and (A245), using Equation (A67), and taking the projection on transverse and longitudinal parts, we find,

(A84) $$\begin{array}{c} D_{aa,T}\left[-\left(\partial_{0}^{2}-v_{T}^{2}\partial_{l}^{2}\right)-i\Pi_{\mathrm{nonl},aa,T}\right]+4id_{aa,T}eg_{aT}\left(|\bar{\psi }{|}^{2}+\Delta \right)D_{T}eg_{aT}\left(|\bar{\psi }{|}^{2}+\Delta \right)=i\delta_{C}, \end{array}$$

and,

(A85) $$\begin{array}{ccc} D_{aa,L}\left[-\left(\partial_{0}^{2}-v_{L}^{2}\partial_{l}^{2}\right)-i\Pi_{\mathrm{nonl},aa,L}\right] & = & i\delta_{C}. \end{array}$$

Similarly taking oo component in Equation (A72) with Equations (A61) and (A245), using Equation (A69), and taking the projection on transverse and longitudinal parts, we find,

(A86) $$\begin{array}{c} D_{oo,T}\left[-\left(\partial_{0}^{2}+\Omega_{T}^{2}\right)-i\Pi_{\mathrm{nonl},oo,T}\right]+4id_{oo,T}eg_{oT}\left(|\bar{\psi }{|}^{2}+\Delta \right)D_{T}eg_{oT}\left(|\bar{\psi }{|}^{2}+\Delta \right)=i\delta_{C}, \end{array}$$

and,

(A87) $$\begin{array}{ccc} D_{oo,L}\left[-\left(\partial_{0}^{2}+\Omega_{L}^{2}\right)-i\Pi_{\mathrm{nonl},oo,L}\right] & = & i\delta_{C}, \end{array}$$

Next we shall introduce $U_{aa,ij}\left(x,y\right)$, $U_{oo,ij}\left(x,y\right)$, $V_{aa,ij}\left(x,y\right)$ and $V_{oo,ij}\left(x,y\right)$ as,

(A88) $$\begin{array}{ccc} U_{aa,ij}\left(x,y\right) & = & 4{\left(eg_{aT}\right)}^{2}\left(|\bar{\psi }{\left(x\right)|}^{2}+\Delta^{11}\left(x,x\right)\right)P_{T,ik}d_{aa,kl}\left(x,y\right)\left(|\bar{\psi }{\left(y\right)|}^{2}+\Delta^{11}\left(y,y\right)\right)P_{T,lj}, \end{array}$$

(A89) $$\begin{array}{ccc} U_{oo,ij}\left(x,y\right) & = & 4{\left(eg_{oT}\right)}^{2}\left(|\bar{\psi }{\left(x\right)|}^{2}+\Delta^{11}\left(x,x\right)\right)P_{T,ik}d_{oo,kl}\left(x,y\right)\left(|\bar{\psi }{\left(y\right)|}^{2}+\Delta^{11}\left(y,y\right)\right)P_{T,lj}, \end{array}$$

(A90) $$\begin{array}{ccc} V_{aa,ij}\left(x,y\right) & = & 4{\left(eg_{aT}\right)}^{2}\left(|\bar{\psi }{\left(x\right)|}^{2}+\Delta^{11}\left(x,x\right)\right)P_{T,ik}D_{kl}\left(x,y\right)\left(|\bar{\psi }{\left(y\right)|}^{2}+\Delta^{11}\left(y,y\right)\right)P_{T,lj}, \end{array}$$

(A91) $$\begin{array}{ccc} V_{oo,ij}\left(x,y\right) & = & 4{\left(eg_{oT}\right)}^{2}\left(|\bar{\psi }{\left(x\right)|}^{2}+\Delta^{11}\left(x,x\right)\right)P_{T,ik}D_{kl}\left(x,y\right)\left(|\bar{\psi }{\left(y\right)|}^{2}+\Delta^{11}\left(y,y\right)\right)P_{T,lj}. \end{array}$$

We expand the Fourier transformation $D_{ij}\left(X,k\right)=\int d^{4}\left(x-y\right)e^{ik\cdot (x-y)}D_{ij}\left(x,y\right)$ by relative coordinate x−y with $X=\frac{x+y}{2}$ by,

(A92) $$\begin{array}{c} D_{ij}\left(X,k\right)=\left(\delta_{ij}-\frac{k_{i}k_{j}}{k^{2}}\right)D_{T}\left(X,k\right)+\frac{k_{i}k_{j}}{k^{2}}D_{L}\left(X,k\right), \end{array}$$

$\Pi_{\mathrm{nonl},ij}\left(X,k\right)$ by $\Pi_{T}\left(X,k\right)$ and $\Pi_{L}\left(X,k\right)$, $D_{aa,ij}\left(X,k\right)$ by $D_{aa,T}\left(X,k\right)$ and $D_{aa,L}\left(X,k\right)$, $D_{oo,ij}\left(X,k\right)$ by $D_{oo,T}\left(X,k\right)$ and $D_{oo,L}\left(X,k\right)$, $\Pi_{aa,ij}\left(X,k\right)$ by $\Pi_{aa,T}\left(X,k\right)$ and $\Pi_{aa,L}\left(X,k\right)$ and $\Pi_{oo,ij}\left(X,k\right)$ by $\Pi_{oo,T}\left(X,k\right)$ and $\Pi_{oo,L}\left(X,k\right)$.

We take up to 1st order in the gradient expansion of convolution in Fourier transformation [108,109,110,111,112] as,

(A93) $$\begin{array}{ccc} \int d\left(x-y\right)e^{ik\cdot (x-y)}\int dzM\left(x,z\right)N\left(z,y\right) & = & M\left(X,k\right)\circ_{P}N\left(X,k\right)\\ & = & M\left(X,k\right)N\left(X,k\right)+\frac{i}{2}{\left\{M,N\right\}}_{P}+O\left({\left(\frac{\partial }{\partial X}\right)}^{2}\right), \end{array}$$

with the Poisson bracket,

(A94) $$\begin{array}{c} {\left\{M,N\right\}}_{P}=\frac{\partial M}{\partial p^{\mu }}\frac{\partial N}{\partial X_{\mu }}-\frac{\partial M}{\partial X^{\mu }}\frac{\partial N}{\partial p_{\mu }}. \end{array}$$

Using the gradient expansion or derivative expansion for the center-of-mass coordinate $X=\frac{x+y}{2}$, we expand time-evolution equations by derivatives and can neglect higher-order derivatives. Taking the matrix notation in the closed-time path, taking the Fourier transformation in Equations (A70) and (A77), we can derive,

(A95) $$\begin{array}{ccc} {\left(k^{2}-\frac{e^{2}\left(|\bar{\psi }{\left(X\right)|}^{2}+\Delta \left(X,X\right)\right)}{m}-i\Pi_{L}\sigma_{z}\right)}^{ab}\circ_{P}D_{L}^{bc} & = & i\sigma_{z}^{ac}, \end{array}$$

(A96) $$\begin{array}{ccc} D_{L}^{ab}\circ_{P}{\left(k^{2}-\frac{e^{2}\left(|\bar{\psi }{\left(X\right)|}^{2}+\Delta \left(X,X\right)\right)}{m}-i\sigma_{z}\Pi_{L}\right)}^{bc} & = & i\sigma_{z}^{ac}, \end{array}$$

with the Pauli matrix $\sigma_{z}=\mathrm{diag}\left(1,-1\right)$ and a,b,c=1,2 in closed-time path. Taking the difference of above two equations, we can derive,

(A97) $$\begin{array}{c} i{\left\{k^{2}-\frac{e^{2}\left(|\bar{\psi }{\left(X\right)|}^{2}+\Delta \left(X,X\right)\right)}{m},D_{L}^{ac}\right\}}_{P}=i{\left[\Pi_{L}\sigma_{z}\circ_{P}D_{L}-D_{L}\circ_{P}\sigma_{z}\Pi_{L}\right]}^{ac}. \end{array}$$

Similarly, using the Fourier transformation $U_{aa,T}\left(X,k\right)$ and $U_{oo,T}\left(X,k\right)$ given by,

(A98) $$\begin{array}{ccc} U_{aa,T}\left(X,k\right) & = & 4{\left(eg_{aT}\right)}^{2}{\left(|\bar{\psi }{\left(X\right)|}^{2}+\Delta^{11}\left(X,X\right)\right)}^{2}d_{aa,T}\left(X,k\right)+O\left({\left(\frac{\partial }{\partial X}\right)}^{2}\right), \end{array}$$

(A99) $$\begin{array}{ccc} U_{oo,T}\left(X,k\right) & = & 4{\left(eg_{aT}\right)}^{2}{\left(|\bar{\psi }{\left(X\right)|}^{2}+\Delta^{11}\left(X,X\right)\right)}^{2}d_{oo,T}\left(X,k\right)+O\left({\left(\frac{\partial }{\partial X}\right)}^{2}\right), \end{array}$$

we can rewrite Equations (A71) and (A78) by,

(A100) $$\begin{array}{ccc} {\left(k^{2}-\frac{e^{2}\left(|\bar{\psi }{|}^{2}+\Delta \right)}{m}-i\Pi_{T}\sigma_{z}+iU_{aa,T}\sigma_{z}+iU_{oo,T}\sigma_{z}\right)}^{ab}\circ_{P}D_{T}^{bc} & = & i\sigma_{z}^{ac}, \end{array}$$

(A101) $$\begin{array}{ccc} D_{T}^{ab}\circ_{P}{\left(k^{2}-\frac{e^{2}\left(|\bar{\psi }{|}^{2}+\Delta \right)}{m}-i\sigma_{z}\Pi_{T}+i\sigma_{z}U_{aa,T}+i\sigma_{z}U_{oo,T}\right)}^{bc} & = & i\sigma_{z}^{ac}, \end{array}$$

Taking the difference of the above two equations, we find,

(A102) $$\begin{array}{cc} & i{\left\{k^{2}-\frac{e^{2}\left(|\bar{\psi }{\left(X\right)|}^{2}+\Delta \left(X,X\right)\right)}{m},D_{T}^{ac}\right\}}_{P}\\ = & i{\left[\left(\Pi_{T}-U_{aa,T}-U_{oo,T}\right)\sigma_{z}\circ_{P}D_{T}-D_{T}\circ_{P}\sigma_{z}\left(\Pi_{T}-U_{aa,T}-U_{oo,T}\right)\right]}^{ac}. \end{array}$$

In Equations (A81) and (A85), we can derive,

(A103) $$\begin{array}{ccc} \left[{\left(k^{0}\right)}^{2}-v_{L}^{2}k^{2}-i\Pi_{aa,L}\sigma_{z}\right]\circ_{P}D_{aa,L} & = & i\sigma_{z}, \end{array}$$

(A104) $$\begin{array}{ccc} D_{aa,L}\circ_{P}\left[{\left(k^{0}\right)}^{2}-v_{L}^{2}k^{2}-i\sigma_{z}\Pi_{aa,L}\right] & = & i\sigma_{z}, \end{array}$$

Taking the difference of the above two equations, we find,

(A105) $$\begin{array}{c} i{\left\{{\left(k^{0}\right)}^{2}-v_{L}^{2}k^{2},D_{aa,L}^{ac}\right\}}_{P}=i{\left[\Pi_{aa,L}\sigma_{z}\circ_{P}D_{aa,L}-D_{aa,L}\circ_{P}\sigma_{z}\Pi_{aa,L}\right]}^{ac}. \end{array}$$

Using the Fourier transformation $V_{aa,T}\left(X,k\right)$ given by,

(A106) $$\begin{array}{c} V_{aa,T}\left(X,k\right)=4{\left(eg_{aT}\right)}^{2}{\left(|\bar{\psi }{\left(X\right)|}^{2}+\Delta^{11}\left(X,X\right)\right)}^{2}D_{T}\left(X,k\right)+O\left({\left(\frac{\partial }{\partial X}\right)}^{2}\right), \end{array}$$

Equations (A80) and (A84) are rewritten by,

(A107) $$\begin{array}{ccc} \left({\left(k^{0}\right)}^{2}-v_{T}^{2}k^{2}-i\Pi_{aa,T}\sigma_{z}\right)\circ_{P}D_{aa,T}+iV_{aa,T}\sigma_{z}\circ_{P}d_{aa,T} & = & i\sigma_{z}, \end{array}$$

(A108) $$\begin{array}{ccc} D_{aa,T}\circ_{P}\left({\left(k^{0}\right)}^{2}-v_{T}^{2}k^{2}-i\sigma_{z}\Pi_{aa,T}\right)+id_{aa,T}\circ_{P}\sigma_{z}V_{aa,T} & = & i\sigma_{z}. \end{array}$$

Taking the difference of the above two equations, we derive,

(A109) $$\begin{array}{ccc} i{\left\{{\left(k^{0}\right)}^{2}-v_{T}^{2}k^{2},D_{aa,T}^{ac}\right\}}_{P} & = & i{\left[\Pi_{aa,T}\sigma_{z}\circ_{P}D_{aa,T}-D_{aa,T}\circ_{P}\sigma_{z}\Pi_{aa,T}\right]}^{ac}\\ & & -i{\left[V_{aa,T}\sigma_{z}\circ_{P}d_{aa,T}-d_{aa,T}\circ_{P}\sigma_{z}V_{aa,T}\right]}^{ac}. \end{array}$$

The oo components in Equations (A83) and (A87) are rewritten by,

(A110) $$\begin{array}{ccc} \left[{\left(k^{0}\right)}^{2}-\Omega_{L}^{2}-i\Pi_{oo,L}\sigma_{z}\right]\circ_{P}D_{oo,L} & = & i\sigma_{z}, \end{array}$$

(A111) $$\begin{array}{ccc} D_{oo,L}\circ_{P}\left[{\left(k^{0}\right)}^{2}-\Omega_{L}^{2}-i\sigma_{z}\Pi_{oo,L}\right] & = & i\sigma_{z}. \end{array}$$

Taking the difference of the above equations, we find,

(A112) $$\begin{array}{c} i{\left\{{\left(k^{0}\right)}^{2}-\Omega_{L}^{2},D_{oo,L}^{ac}\right\}}_{P}=i{\left[\Pi_{oo,L}\sigma_{z}\circ_{P}D_{oo,L}-D_{oo,L}\circ_{P}\sigma_{z}\Pi_{oo,L}\right]}^{ac}. \end{array}$$

Using the Fourier transformation $V_{oo,T}\left(X,k\right)$ given by,

(A113) $$\begin{array}{c} V_{oo,T}\left(X,k\right)=4{\left(eg_{oT}\right)}^{2}{\left(|\bar{\psi }{\left(X\right)|}^{2}+\Delta^{11}\left(X,X\right)\right)}^{2}D_{T}\left(X,k\right)+O\left({\left(\frac{\partial }{\partial X}\right)}^{2}\right), \end{array}$$

the Fourier transformation of Equations (A82) and (A86) are written by,

(A114) $$\begin{array}{ccc} \left({\left(k^{0}\right)}^{2}-\Omega_{T}^{2}-i\Pi_{oo,T}\sigma_{z}\right)\circ_{P}D_{oo,T}+iV_{oo,T}\sigma_{z}\circ_{P}d_{oo,T} & = & i\sigma_{z}, \end{array}$$

(A115) $$\begin{array}{ccc} D_{oo,T}\circ_{P}\left({\left(k^{0}\right)}^{2}-\Omega_{T}^{2}-i\sigma_{z}\Pi_{oo,T}\right)+id_{oo,T}\circ_{P}\sigma_{z}V_{oo,T} & = & i\sigma_{z}. \end{array}$$

Taking the difference of the above two equations, we derive,

(A116) $$\begin{array}{ccc} i{\left\{{\left(k^{0}\right)}^{2}-\Omega_{T}^{2},D_{oo,T}^{ac}\right\}}_{P} & = & i{\left[\Pi_{oo,T}\sigma_{z}\circ_{P}D_{oo,T}-D_{oo,T}\circ_{P}\sigma_{z}\Pi_{oo,T}\right]}^{ac}\\ & & -i{\left[V_{oo,T}\sigma_{z}\circ_{P}d_{oo,T}-d_{oo,T}\circ_{P}\sigma_{z}V_{oo,T}\right]}^{ac}. \end{array}$$

Finally we investigate the Kadanoff–Baym equation for charged bosons in Equation (A9) or,

(A117) $$\begin{array}{ccc} i\left(\Delta_{0}^{-1}-\tilde{\Sigma }\right)\Delta & = & i\delta_{C}, \end{array}$$

(A118) $$\begin{array}{ccc} \Delta \left(i\Delta_{0}^{-1}-i\tilde{\Sigma }\right) & = & i\delta_{C}. \end{array}$$

We define the gauge-invariant Green’s function G(x,y) [113,114] as,

(A119) $$\begin{array}{ccc} G(x,y) & \equiv & e^{-ie\int_{y}^{x}dz_{\mu }A^{\mu }\left(z\right)}\Delta \left(x,y\right), \end{array}$$

and self-energy,

(A120) $$\begin{array}{ccc} \Sigma (x,y) & \equiv & e^{-ie\int_{y}^{x}dz_{\mu }A^{\mu }\left(z\right)}\tilde{\Sigma }\left(x,y\right)\\ & = & -i\delta_{C}\left(x-y\right)\Sigma_{\mathrm{loc}}+\Sigma_{\mathrm{nonl}}\left(x,y\right). \end{array}$$

Taking the matrix notation in closed-time path, multiplying $e^{-ie\int_{y}^{x}dz_{\mu }A^{\mu }\left(z\right)}$ in Equation (A117) with Equation (8), and taking the Fourier transformation $\int d\left(x-y\right)e^{ip\cdot (x-y)}$ of relative coordinate x−y, we arrive at,

(A121) $$\begin{array}{ccc} i\left(G_{0}^{-1}-\Sigma \sigma_{z}\right)\circ G & = & i\sigma_{z}, \end{array}$$

where,

(A122) $$\begin{array}{c} iG_{0}^{-1}\left(p\right)=p^{0}-\frac{p^{2}}{2m}-g_{aL}\left(\nabla_{X,i}{\bar{Q}}_{aL}^{i}\right)-g_{oL}\left(\nabla_{X,i}{\bar{Q}}_{oL}^{i}\right)+2g_{aT}\left(p^{i}{\bar{Q}}_{aT,i}\right)+2g_{oT}\left(p^{i}{\bar{Q}}_{oT,i}\right), \end{array}$$

and the generalized Moyal product ∘ in [112] represents,

(A123) $$\begin{array}{c} M\circ N=MN+\frac{i}{2}\left\{M,N\right\}, \end{array}$$

with,

(A124) $$\begin{array}{c} \left\{M,N\right\}={\left\{M,N\right\}}_{P}+eE\cdot \left(\frac{\partial M}{\partial p}\frac{\partial N}{\partial p^{0}}-\frac{\partial M}{\partial p^{0}}\frac{\partial N}{\partial p}\right)-eB\cdot \left(\frac{\partial M}{\partial p}\times \frac{\partial N}{\partial p}\right)+O\left({\left(\frac{\partial }{\partial X}\right)}^{2}\right), \end{array}$$

with $\frac{\partial }{\partial p}=\frac{\partial }{\partial p^{i}}$, electric field E and magnetic field B. Similarly Equation (A118) is written by,

(A125) $$\begin{array}{ccc} G\circ i\left(G_{0}^{-1}-\sigma_{z}\Sigma \right) & = & i\sigma_{z}, \end{array}$$

Taking the difference between Equations (A121) and (A125), we arrive at,

(A126) $$\begin{array}{c} i\left\{p^{0}-\frac{p^{2}}{2m}-g_{aL}\left(\nabla_{X,i}{\bar{Q}}_{aL}^{i}\right)-g_{oL}\left(\nabla_{X,i}{\bar{Q}}_{oL}^{i}\right)+2g_{aT}\left(p^{i}{\bar{Q}}_{aT,i}\right)+2g_{oT}\left(p^{i}{\bar{Q}}_{oT,i}\right)-\Sigma_{\mathrm{loc}}\left(X\right),G^{ab}\right\}\\ =i{\left[\Sigma_{\mathrm{nonl}}\sigma_{z}\circ G-G\circ \sigma_{z}\Sigma_{\mathrm{nonl}}\right]}^{ab}, \end{array}$$

with,

(A127) $$\begin{array}{c} \Sigma_{\mathrm{loc}}\left(X\right)=\frac{e^{2}}{2m}D_{ii}\left(X,X\right)+eg_{aT}\left(D_{a\gamma ,T,ii}\left(X,X\right)+D_{\gamma a,T,ii}\left(X,X\right)\right)+eg_{oT}\left(D_{o\gamma ,T,ii}\left(X,X\right)+D_{\gamma o,T,ii}\left(X,X\right)\right), \end{array}$$

derived from Equations (A51) and (A52). In Equations (A121) and (A125), we can derive,

(A128) $$\begin{array}{ccc} \left[iG_{0}^{-1}\left(p\right)-\Sigma_{\mathrm{loc}}\left(X\right)-\Sigma_{R}\left(X,p\right)\right]G_{R}\left(X,p\right) & = & -1, \end{array}$$

(A129) $$\begin{array}{ccc} \left\{iG_{0}^{-1}-\Sigma_{\mathrm{loc}}-\Sigma_{R},G_{R}\right\} & = & 0, \end{array}$$

with retarded Green’s functions $G_{R}=i\left(G^{11}-G^{12}\right)$ and retarded self-energy $\Sigma_{R}=i\left(\Sigma_{\mathrm{nonl}}^{11}-\Sigma_{\mathrm{nonl}}^{12}\right)$. The solution of the above equations is given by,

(A130) $$\begin{array}{c} G_{R}\left(X,p\right)=\frac{-1}{iG_{0}^{-1}\left(p\right)-\Sigma_{\mathrm{loc}}\left(X\right)-\Sigma_{R}\left(X,p\right)}. \end{array}$$

Since we can write GR(X,p)=i(G11−G12)=ReGR+12ρ with the pure imaginary spectral function $\rho \equiv i(G^{21}-G^{12})$ and real $G^{21}$ and $G^{12}$ and ΣR=ReΣR+12Σρ with the pure imaginary spectral part in self-energy $\Sigma_{\rho }$, we can derive the solution of spectral function ρ by taking the imaginary part of the above equation.

## Appendix D. Proof of the H-Theorem

We shall introduce a kinetic entropy current in 1st order approximation in the gradient expansion in our model, and show the H-theorem for 1-loop non-local self-energy in Hartree–Fock approximation. We adopt the method given in [95,96,97,98,99,100,101].

We begin with the kinetic entropy current for charged bosons. Taking the 1st order in the gradient expansion, we can write time-evolution equations for (a,b)=(1,2) and (2,1) in Equation (A126) as,

(A131) p0−p22m−gaL∇X,iQ¯aLi−goL∇X,iQ¯oLi+2gaTpiQ¯aT,i+2goTpiQ¯oT,i−Σloc(X),Gab−ReΣR,Gab+ReGR,Σab=Σ21G12−G21Σ12,

where we have used ReΣR=i2(Σnonl11−Σnonl22) and ReGR=i2(G11−G22) with retarded self-energy $\Sigma_{R}=i\left(\Sigma_{\mathrm{nonl}}^{11}-\Sigma_{\mathrm{nonl}}^{12}\right)$ and Green’s function $G_{R}=i\left(G^{11}-G^{12}\right)$. The above equation has a similar form as [98,101]. Using the Kadanoff–Baym Ansatz $G^{12}=\frac{\rho }{i}f$, $G^{21}=\frac{\rho }{i}\left(1+f\right)$ involving the spectral function ρ and the distribution function *f*, $\Sigma^{12}=\Sigma_{\rho }\gamma$, $\Sigma^{21}=\Sigma_{\rho }\left(1+\gamma \right)$ involving the spectral part of self-energy $\Sigma_{\rho }$ and the distribution function γ for self-energy, and neglecting higher order terms in the gradient expansion, we can use,

(A132) $$\begin{array}{c} f∼\gamma +O\left(\frac{\partial }{\partial X}\right), \end{array}$$

which is due to $\Sigma^{21}G^{12}-G^{21}\Sigma^{12}\propto f-\gamma =O\left(\frac{\partial }{\partial X}\right)$ in Equation (A131). Multiplying $\ln\frac{iG^{12}}{\rho }$ for (a,b)=(1,2) component in Equation (A131), multiplying and $\ln\frac{iG^{21}}{\rho }$ for (a,b)=(2,1) component in Equation (A131), taking the difference of the two, and integrate with $\int_{p}=\int \frac{d^{4}p}{{\left(2\pi \right)}^{4}}$, we arrive at,

(A133) $$\begin{array}{c} \partial_{\mu }s_{\mathrm{cb}}^{\mu }=\int_{p}\left(\Sigma^{12}\left(X,p\right)G^{21}\left(X,p\right)-\Sigma^{21}\left(X,p\right)G^{12}\left(X,p\right)\right)\ln\frac{G^{12}\left(X,p\right)}{G^{21}\left(X,p\right)}, \end{array}$$

with the definition of kinetic entropy current for charged bosons given by,

(A134) scbμ(X)=∫pδ0μ+pimδiμ+∂∂pμ(2gaTQ¯aTj+2goTQ¯oTj)pj−ReΣR)ρi+∂ReGR∂pμΣρiσ[f],

with,

(A135) $$\begin{array}{c} \sigma [f]=(1+f)\ln(1+f)-f\ln f. \end{array}$$

Next we investigate the kinetic entropy current for photons. Using the relations (A97) and (A102), and the Kadanoff–Baym Ansatz $D_{T}^{12}=-i\rho_{T}f_{T}$, $D_{T}^{21}=-i\rho_{T}\left(1+f_{T}\right)$, $D_{L}^{12}=-i\rho_{L}f_{L}$, $D_{L}^{21}=-i\rho_{L}\left(1+f_{L}\right)$, $\Pi_{T}^{12}=-i\Pi_{\rho ,T}\gamma_{T}$, $\Pi_{T}^{21}=-i\Pi_{\rho ,T}\left(1+\gamma_{T}\right)$, $\Pi_{L}^{12}=-i\Pi_{\rho ,L}f_{L}$, $\Pi_{L}^{21}=-i\Pi_{\rho ,L}\left(1+\gamma_{L}\right)$, $U_{aa,T}^{12}=-iU_{\rho ,aa,T}\gamma_{aa,U}$, $U_{aa,T}^{21}=-iU_{\rho ,aa,T}\left(1+\gamma_{aa,U}\right)$, $U_{oo,T}^{12}=-iU_{\rho ,oo,T}\gamma_{oo,U}$, and $U_{oo,T}^{21}=-iU_{\rho ,oo,T}\left(1+\gamma_{oo,U}\right)$, with $\rho_{T}=i\left(D_{T}^{21}-D_{T}^{12}\right)$, $\rho_{L}=i\left(D_{L}^{21}-D_{L}^{12}\right)$, $\Pi_{\rho ,T}=i\left(\Pi_{T}^{21}-\Pi_{T}^{12}\right)$, $\Pi_{\rho ,L}=i\left(\Pi_{L}^{21}-\Pi_{L}^{12}\right)$, $U_{\rho ,aa,T}=i\left(U_{aa,T}^{21}-U_{aa,T}^{12}\right)$, and $U_{\rho ,oo,T}=i\left(U_{oo,T}^{21}-U_{oo,T}^{12}\right)$ where ρ or subscript ρ represents spectral parts of Green’s functions and self-energy and *f* and γ represent distribution functions, we find,

(A136) $$\begin{array}{c} f_{T}∼\gamma_{T}∼\gamma_{aa,U}∼\gamma_{oo,U}, \end{array}$$

and,

(A137) $$\begin{array}{c} f_{L}∼\gamma_{L}. \end{array}$$

Using a similar method to the above procedure of charged bosons, we can derive,

(A138) $$\begin{array}{c} \partial_{\mu }s_{\mathrm{photon},L}^{\mu }=\frac{1}{2}\int_{k}\left(\Pi_{L}^{12}\left(X,k\right)D_{L}^{21}\left(X,k\right)-\Pi_{L}^{21}\left(X,k\right)D_{L}^{12}\left(X,k\right)\right)\ln\frac{D_{L}^{12}\left(X,k\right)}{D_{L}^{21}\left(X,k\right)}, \end{array}$$

and,

(A139) $$\begin{array}{ccc} \partial_{\mu }s_{\mathrm{photon},T}^{\mu } & = & \int_{k}\left(\Pi_{T}^{12}\left(X,k\right)D_{T}^{21}\left(X,k\right)-\Pi_{T}^{21}\left(X,k\right)D_{T}^{12}\left(X,k\right)\right)\ln\frac{D_{T}^{12}\left(X,k\right)}{D_{T}^{21}\left(X,k\right)}\\ & & -\int_{k}\left(U_{aa,T}^{12}\left(X,k\right)D_{T}^{21}\left(X,k\right)-U_{aa,T}^{21}\left(X,k\right)D_{T}^{12}\left(X,k\right)\right)\ln\frac{D_{T}^{12}\left(X,k\right)}{D_{T}^{21}\left(X,k\right)}\\ & & -\int_{k}\left(U_{oo,T}^{12}\left(X,k\right)D_{T}^{21}\left(X,k\right)-U_{oo,T}^{21}\left(X,k\right)D_{T}^{12}\left(X,k\right)\right)\ln\frac{D_{T}^{12}\left(X,k\right)}{D_{T}^{21}\left(X,k\right)}, \end{array}$$

with the entropy current for longitudinal and transverse photons defined by,

(A140) sphoton,Lμ=∫kkμ−12∂ReΠR,L∂kμρLi+12∂ReDR,L∂kμΠρ,Liσ[fL],

(A141) sphoton,Tμ=2∫k[kμ−12∂ReΠR,T−Uaa,R,T−Uoo,R,T∂kμρTi+12∂ReDR,T∂kμΠρ,T−Uaa,ρ,T−Uoo,ρ,Ti]σ[fT],

with $\Pi_{R,L}=i\left(\Pi_{L}^{11}-\Pi_{L}^{12}\right)$, $D_{R,L}=i\left(D_{L}^{11}-D_{L}^{12}\right)$, $\Pi_{R,T}=i\left(\Pi_{T}^{11}-\Pi_{T}^{12}\right)$, $D_{R,T}=i\left(D_{T}^{11}-D_{T}^{12}\right)$, $U_{aa,R,T}=i\left(U_{aa,T}^{11}-U_{aa,T}^{12}\right)$, and $U_{oo,R,T}=i\left(U_{oo,T}^{11}-U_{oo,T}^{12}\right)$.

Next we investigate a kinetic entropy current for acoustic phonons. Using Equations (A105) and (A109) and the Kadanoff–Baym Ansatz $D_{aa,T}^{12}=-i\rho_{aa,T}f_{aa,T}$, $D_{aa,T}^{21}=-i\rho_{aa,T}\left(1+f_{aa,T}\right)$, $D_{aa,L}^{12}=-i\rho_{aa,L}f_{aa,L}$, $D_{aa,L}^{21}=-i\rho_{aa,L}\left(1+f_{aa,L}\right)$, $d_{aa,T}^{12}=-id_{aa,\rho ,T}\gamma_{d,aa,T}$, $d_{aa,T}^{21}=-id_{aa,\rho ,T}\left(1+\gamma_{d,aa,T}\right)$, $\Pi_{aa,T}^{12}=-i\Pi_{aa,\rho ,T}\gamma_{aa,T}$, $\Pi_{aa,T}^{21}=-i\Pi_{aa,\rho ,T}\left(1+\gamma_{aa,T}\right)$, $\Pi_{aa,L}^{12}=-i\Pi_{aa,\rho ,L}\gamma_{aa,L}$, $\Pi_{aa,L}^{21}=-i\Pi_{aa,\rho ,L}\left(1+\gamma_{aa,L}\right)$, $V_{aa,T}^{12}=-iV_{aa,\rho ,T}\gamma_{aa,V}$, and $V_{aa,T}^{21}=-iV_{aa,\rho ,T}\left(1+\gamma_{aa,V}\right)$ with $\rho_{aa,T}=i\left(D_{aa,T}^{21}-D_{aa,T}^{12}\right)$, $\rho_{aa,L}=i\left(D_{aa,L}^{21}-D_{aa,L}^{12}\right)$, $d_{aa,\rho ,T}=i\left(d_{aa,T}^{21}-d_{aa,T}^{12}\right)$, $\Pi_{aa,\rho ,T}=i\left(\Pi_{aa,T}^{21}-\Pi_{aa,T}^{12}\right)$, $\Pi_{aa,\rho ,L}=i\left(\Pi_{aa,L}^{21}-\Pi_{aa,L}^{12}\right)$, and $V_{aa,\rho ,T}=i\left(V_{aa,T}^{21}-V_{aa,T}^{12}\right)$ where ρ or subscript ρ represents spectral parts of Green’s functions and self-energy and *f* and γ represent distribution functions, we find,

(A142) $$\begin{array}{c} f_{aa,L}∼\gamma_{aa,L}, \end{array}$$

and,

(A143) $$\begin{array}{c} f_{aa,T}∼\gamma_{aa,T},\;\;\;\gamma_{aa,V}∼\gamma_{d,aa,T}, \end{array}$$

and we also find,

(A144) $$\begin{array}{c} \gamma_{aa,T}∼\gamma_{d,aa,T}, \end{array}$$

by Equation (A65). Using the relations (A142)–(A144), and adopting a similar procedure in Equations (A105) and (A109) to the procedure we adopted for charged bosons, we can derive,

(A145) $$\begin{array}{c} \partial_{\mu }s_{a,L}^{\mu }=\frac{1}{2}\int_{k}\left(\Pi_{aa,L}^{12}\left(X,k\right)D_{aa,L}^{21}\left(X,k\right)-\Pi_{aa,L}^{21}\left(X,k\right)D_{aa,L}^{12}\left(X,k\right)\right)\ln\frac{D_{aa,L}^{12}\left(X,k\right)}{D_{aa,L}^{21}\left(X,k\right)}, \end{array}$$

and,

(A146) $$\begin{array}{ccc} \partial_{\mu }s_{a,T}^{\mu } & = & \int_{k}\left(\Pi_{aa,T}^{12}\left(X,k\right)D_{aa,T}^{21}\left(X,k\right)-\Pi_{aa,T}^{21}\left(X,k\right)D_{aa,T}^{12}\left(X,k\right)\right)\ln\frac{D_{aa,T}^{12}\left(X,k\right)}{D_{aa,T}^{21}\left(X,k\right)}\\ & & -\int_{k}\left(V_{aa,T}^{12}\left(X,k\right)d_{aa,T}^{21}\left(X,k\right)-V_{aa,T}^{21}\left(X,k\right)d_{aa,T}^{12}\left(X,k\right)\right)\ln\frac{D_{aa,T}^{12}\left(X,k\right)}{D_{aa,T}^{21}\left(X,k\right)}, \end{array}$$

where the entropy current for longitudinal and transverse acoustic phonons is defined by,

(A147) sa,Lμ(X)=∫kk0δ0μ+vL2kiδiμ−12∂ReΠaa,R,L∂kμρaa,Li+12∂ReDaa,R,L∂kμΠaa,ρ,Liσ[faa,L],

(A148) sa,Tμ(X)=2∫k[k0δ0μ+vT2kiδiμ−12∂ReΠaa,R,T∂kμρaa,Ti+12∂ReDaa,R,T∂kμΠaa,ρ,Ti+12ReVaa,R,T∂kμdaa,ρ,Ti−∂Redaa,R,T∂kμVaa,ρ,Ti]σ[faa,T].

Next we investigate a kinetic entropy current for optical phonons. Using Equations (A112) and (A116) and the Kadanoff–Baym Ansatz $D_{oo,T}^{12}=-i\rho_{oo,T}f_{oo,T}$, $D_{oo,T}^{21}=-i\rho_{oo,T}\left(1+f_{oo,T}\right)$, $D_{oo,L}^{12}=-i\rho_{oo,L}f_{oo,L}$, $D_{oo,L}^{21}=-i\rho_{oo,L}\left(1+f_{oo,L}\right)$, $d_{oo,T}^{12}=-id_{oo,\rho ,T}\gamma_{d,oo,T}$, $d_{oo,T}^{21}=-id_{oo,\rho ,T}\left(1+\gamma_{d,oo,T}\right)$, $\Pi_{oo,T}^{12}=-i\Pi_{oo,\rho ,T}\gamma_{oo,T}$, $\Pi_{oo,T}^{21}=-i\Pi_{oo,\rho ,T}\left(1+\gamma_{oo,T}\right)$, $\Pi_{oo,L}^{12}=-i\Pi_{oo,\rho ,L}\gamma_{oo,L}$, $\Pi_{oo,L}^{21}=-i\Pi_{oo,\rho ,L}\left(1+\gamma_{oo,L}\right)$, $V_{oo,T}^{12}=-iV_{oo,\rho ,T}\gamma_{oo,V}$, and $V_{oo,T}^{21}=-iV_{oo,\rho ,T}\left(1+\gamma_{oo,V}\right)$ with $\rho_{oo,T}=i\left(D_{oo,T}^{21}-D_{oo,T}^{12}\right)$, $\rho_{oo,L}=i\left(D_{oo,L}^{21}-D_{oo,L}^{12}\right)$, $d_{oo,\rho ,T}=i\left(d_{oo,T}^{21}-d_{oo,T}^{12}\right)$, $\Pi_{oo,\rho ,T}=i\left(\Pi_{oo,T}^{21}-\Pi_{oo,T}^{12}\right)$, $\Pi_{oo,\rho ,L}=i\left(\Pi_{oo,L}^{21}-\Pi_{oo,L}^{12}\right)$, and $V_{oo,\rho ,T}=i\left(V_{oo,T}^{21}-V_{oo,T}^{12}\right)$ where ρ or subscript ρ represents spectral parts of Green’s functions and self-energy and *f* and γ represent distribution functions, we find,

(A149) $$\begin{array}{c} f_{oo,L}∼\gamma_{oo,L}, \end{array}$$

and,

(A150) $$\begin{array}{c} f_{oo,T}∼\gamma_{oo,T},\;\;\;\gamma_{oo,V}∼\gamma_{d,oo,T}, \end{array}$$

and we also find,

(A151) $$\begin{array}{c} \gamma_{oo,T}∼\gamma_{d,oo,T}, \end{array}$$

by Equation (A68). Using Equations (A149)–(A151), and adopting the procedure in Equations (A112) and (A116) to derive the divergence of the entropy current, we arrive at,

(A152) $$\begin{array}{c} \partial_{\mu }s_{o,L}^{\mu }=\frac{1}{2}\int_{k}\left(\Pi_{oo,L}^{12}\left(X,k\right)D_{oo,L}^{21}\left(X,k\right)-\Pi_{oo,L}^{21}\left(X,k\right)D_{oo,L}^{12}\left(X,k\right)\right)\ln\frac{D_{oo,L}^{12}\left(X,k\right)}{D_{oo,L}^{21}\left(X,k\right)}, \end{array}$$

and,

(A153) $$\begin{array}{ccc} \partial_{\mu }s_{o,T}^{\mu } & = & \int_{k}\left(\Pi_{oo,T}^{12}\left(X,k\right)D_{oo,T}^{21}\left(X,k\right)-\Pi_{oo,T}^{21}\left(X,k\right)D_{oo,T}^{12}\left(X,k\right)\right)\ln\frac{D_{oo,T}^{12}\left(X,k\right)}{D_{oo,T}^{21}\left(X,k\right)}\\ & & -\int_{k}\left(V_{oo,T}^{12}\left(X,k\right)d_{oo,T}^{21}\left(X,k\right)-V_{oo,T}^{21}\left(X,k\right)d_{oo,T}^{12}\left(X,k\right)\right)\ln\frac{D_{oo,T}^{12}\left(X,k\right)}{D_{oo,T}^{21}\left(X,k\right)}, \end{array}$$

where the entropy current for longitudinal and transverse optical phonons is defined by,

(A154)   so,Lμ(X)=∫kk0δ0μ−12∂ReΠoo,R,L∂kμρoo,Li+12∂ReDoo,R,L∂kμΠoo,ρ,Liσ[foo,L],

(A155) so,Tμ(X)=2∫k[k0δ0μ−12∂ReΠoo,R,T∂kμρoo,Ti+12∂ReDoo,R,T∂kμΠoo,ρ,Ti+12ReVoo,R,T∂kμdoo,ρ,Ti−∂Redoo,R,T∂kμVoo,ρ,Ti]σ[foo,T].

Next we shall write self-energy derived by differentiating Equations (A53)–(A57), and $U_{aa,T}\left(X,k\right)$, $U_{oo,T}\left(X,k\right)$, $V_{aa,T}\left(X,k\right)$ and $V_{oo,T}\left(X,k\right)$. Using Equations (A98), (A99), (A106) and (A113), we find,

(A156) $$\begin{array}{ccc} U_{aa,T}^{12}\left(X,k\right) & = & 4{\left(eg_{aT}\right)}^{2}{\left(|\bar{\psi }{\left(X\right)|}^{2}+\int_{p}F\left(X,p\right)\right)}^{2}d_{aa,T}^{12}\left(X,k\right), \end{array}$$

(A157) $$\begin{array}{ccc} U_{aa,T}^{21}\left(X,k\right) & = & 4{\left(eg_{aT}\right)}^{2}{\left(|\bar{\psi }{\left(X\right)|}^{2}+\int_{p}F\left(X,p\right)\right)}^{2}d_{aa,T}^{21}\left(X,k\right), \end{array}$$

(A158) $$\begin{array}{ccc} U_{oo,T}^{12}\left(X,k\right) & = & 4{\left(eg_{oT}\right)}^{2}{\left(|\bar{\psi }{\left(X\right)|}^{2}+\int_{p}F\left(X,p\right)\right)}^{2}d_{oo,T}^{12}\left(X,k\right), \end{array}$$

(A159) $$\begin{array}{ccc} U_{oo,T}^{21}\left(X,k\right) & = & 4{\left(eg_{oT}\right)}^{2}{\left(|\bar{\psi }{\left(X\right)|}^{2}+\int_{p}F\left(X,p\right)\right)}^{2}d_{oo,T}^{21}\left(X,k\right), \end{array}$$

with the statistical function $F\left(x,y\right)=\frac{G^{12}\left(x,y\right)+G^{21}\left(x,y\right)}{2}$ and its Fourier transformation $F\left(X,p\right)=\int_{p}e^{ip\cdot (x-y)}F\left(x,y\right)$, and,

(A160) $$\begin{array}{ccc} V_{aa,T}^{12}\left(X,k\right) & = & 4{\left(eg_{aT}\right)}^{2}{\left(|\bar{\psi }{\left(X\right)|}^{2}+\int_{p}F\left(X,p\right)\right)}^{2}D_{T}^{12}\left(X,k\right), \end{array}$$

(A161) $$\begin{array}{ccc} V_{aa,T}^{21}\left(X,k\right) & = & 4{\left(eg_{aT}\right)}^{2}{\left(|\bar{\psi }{\left(X\right)|}^{2}+\int_{p}F\left(X,p\right)\right)}^{2}D_{T}^{21}\left(X,k\right), \end{array}$$

(A162) $$\begin{array}{ccc} V_{oo,T}^{12}\left(X,k\right) & = & 4{\left(eg_{oT}\right)}^{2}{\left(|\bar{\psi }{\left(X\right)|}^{2}+\int_{p}F\left(X,p\right)\right)}^{2}D_{T}^{12}\left(X,k\right), \end{array}$$

(A163) $$\begin{array}{ccc} V_{oo,T}^{21}\left(X,k\right) & = & 4{\left(eg_{oT}\right)}^{2}{\left(|\bar{\psi }{\left(X\right)|}^{2}+\int_{p}F\left(X,p\right)\right)}^{2}D_{T}^{21}\left(X,k\right). \end{array}$$

The $U_{aa,T}\left(X,k\right)$, $U_{oo,T}\left(X,k\right)$, $V_{aa,T}\left(X,k\right)$ and $V_{oo,T}\left(X,k\right)$ terms represent phonon–photon exchange via charged bosons. Using the Fourier transformation of self-energy, we arrive at the self-energy for charged bosons,

(A164) $$\begin{array}{ccc} \Sigma^{12}\left(X,p\right) & = & \Sigma^{\left(c\right),12}\left(X,p\right)+\Sigma^{\left(d\right),12}\left(X,p\right)+\Sigma^{\left(e\right),12}\left(X,p\right)\\ & & +\Sigma^{\left(f\right),12}\left(X,p\right)+\Sigma^{\left(g\right),12}\left(X,p\right)+\Sigma^{\left(h\right),12}\left(X,p\right), \end{array}$$

(A165) $$\begin{array}{ccc} \Sigma^{12}\left(X,p\right) & = & \Sigma^{\left(c\right),21}\left(X,p\right)+\Sigma^{\left(d\right),21}\left(X,p\right)+\Sigma^{\left(e\right),21}\left(X,p\right)\\ & & +\Sigma^{\left(f\right),21}\left(X,p\right)+\Sigma^{\left(g\right),21}\left(X,p\right)+\Sigma^{\left(h\right),21}\left(X,p\right), \end{array}$$

the self-energy for transverse photons,

(A166) $$\begin{array}{ccc} \Pi_{T}^{12}\left(X,k\right) & = & \Pi_{T}^{\left(c\right),12}\left(X,k\right)+\Pi_{T}^{\left(d\right),12}\left(X,k\right)+\Pi_{T}^{\left(g\right),12}\left(X,k\right)+\Pi_{T}^{\left(h\right),12}\left(X,k\right), \end{array}$$

(A167) $$\begin{array}{ccc} \Pi_{T}^{21}\left(X,k\right) & = & \Pi_{T}^{\left(c\right),21}\left(X,k\right)+\Pi_{T}^{\left(d\right),21}\left(X,k\right)+\Pi_{T}^{\left(g\right),21}\left(X,k\right)+\Pi_{T}^{\left(h\right),21}\left(X,k\right), \end{array}$$

the self-energy for longitudinal photons,

(A168) $$\begin{array}{ccc} \Pi_{L}^{12}\left(X,k\right) & = & \Pi_{L}^{\left(c\right),12}\left(X,k\right)+\Pi_{L}^{\left(g\right),12}\left(X,k\right), \end{array}$$

(A169) $$\begin{array}{ccc} \Pi_{L}^{21}\left(X,k\right) & = & \Pi_{L}^{\left(c\right),21}\left(X,k\right)+\Pi_{L}^{\left(g\right),21}\left(X,k\right), \end{array}$$

the self-energy for transverse acoustic and optical phonons,

(A170) $$\begin{array}{ccc} \Pi_{aa,T}^{12}\left(X,k\right) & = & \Pi_{aa,T}^{\left(e\right),12}\left(X,k\right)+\Pi_{aa,T}^{\left(h\right),12}\left(X,k\right), \end{array}$$

(A171) $$\begin{array}{ccc} \Pi_{aa,T}^{21}\left(X,k\right) & = & \Pi_{aa,T}^{\left(e\right),21}\left(X,k\right)+\Pi_{aa,T}^{\left(h\right),21}\left(X,k\right), \end{array}$$

(A172) $$\begin{array}{ccc} \Pi_{oo,T}^{12}\left(X,k\right) & = & \Pi_{oo,T}^{\left(e\right),12}\left(X,k\right)+\Pi_{oo,T}^{\left(h\right),12}\left(X,k\right), \end{array}$$

(A173) $$\begin{array}{ccc} \Pi_{oo,T}^{21}\left(X,k\right) & = & \Pi_{oo,T}^{\left(e\right),21}\left(X,k\right)+\Pi_{oo,T}^{\left(h\right),21}\left(X,k\right), \end{array}$$

the self-energy for longitudinal acoustic and optical phonons,

(A174) $$\begin{array}{ccc} \Pi_{aa,L}^{12}\left(X,k\right) & = & \Pi_{aa,L}^{\left(f\right),12}\left(X,k\right), \end{array}$$

(A175) $$\begin{array}{ccc} \Pi_{aa,L}^{21}\left(X,k\right) & = & \Pi_{aa,L}^{\left(f\right),21}\left(X,k\right), \end{array}$$

(A176) $$\begin{array}{ccc} \Pi_{oo,L}^{12}\left(X,k\right) & = & \Pi_{oo,L}^{\left(f\right),12}\left(X,k\right), \end{array}$$

(A177) $$\begin{array}{ccc} \Pi_{oo,L}^{21}\left(X,k\right) & = & \Pi_{oo,L}^{\left(f\right),21}\left(X,k\right). \end{array}$$

Differentiating Equation (A53) by Green’s functions as $\tilde{\Sigma }\left(x,y\right)=\frac{1}{2}\frac{\delta i\Gamma_{2}}{\delta \Delta (y,x)}$ and $\Pi \left(x,y\right)=\frac{\delta i\Gamma_{2}}{\delta D(y,x)}$ with $\Sigma \left(x,y\right)=e^{-ie\int_{y}^{x}dz_{\mu }A^{\mu }}\tilde{\Sigma }\left(x,y\right)$, and adopting the Fourier transformation of the relative coordinate x−y with $\int d\left(x-y\right)e^{ip\cdot (x-y)}\times$, we can derive the self-energy for charged bosons,

(A178) $$\begin{array}{ccc} \Sigma^{\left(c\right),12}\left(p\right) & = & -\frac{e^{2}}{4m^{2}}\int_{k,l}\delta_{k+l-p}G^{12}\left(l\right)\left[4\left(p^{2}-\frac{{\left(p\cdot k\right)}^{2}}{k^{2}}\right)D_{T}^{12}\left(k\right)+\frac{{\left(k^{2}-2p\cdot k\right)}^{2}}{k^{2}}D_{L}^{12}\left(k\right)\right], \end{array}$$

(A179) $$\begin{array}{ccc} \Sigma^{\left(c\right),21}\left(p\right) & = & -\frac{e^{2}}{4m^{2}}\int_{k,l}\delta_{k+l-p}G^{21}\left(l\right)\left[4\left(p^{2}-\frac{{\left(p\cdot k\right)}^{2}}{k^{2}}\right)D_{T}^{21}\left(k\right)+\frac{{\left(k^{2}-2p\cdot k\right)}^{2}}{k^{2}}D_{L}^{21}\left(k\right)\right], \end{array}$$

where $\delta_{k+l-p}={\left(2\pi \right)}^{4}\delta^{4}\left(k+l-p\right)$ and we have omitted the variable *X*, and we can derive the self-energy for photons,

(A180) $$\begin{array}{ccc} \Pi_{T}^{\left(c\right),12}\left(k\right) & = & -\frac{e^{2}}{8m^{2}}\int_{p,l}\delta_{k+l-p}4\left(p^{2}-\frac{{\left(p\cdot k\right)}^{2}}{k^{2}}\right)G^{12}\left(p\right)G^{21}\left(l\right), \end{array}$$

(A181) $$\begin{array}{ccc} \Pi_{T}^{\left(c\right),21}\left(k\right) & = & -\frac{e^{2}}{8m^{2}}\int_{p,l}\delta_{k+l-p}4\left(p^{2}-\frac{{\left(p\cdot k\right)}^{2}}{k^{2}}\right)G^{21}\left(p\right)G^{12}\left(l\right), \end{array}$$

(A182) $$\begin{array}{ccc} \Pi_{L}^{\left(c\right),12}\left(k\right) & = & -\frac{e^{2}}{4m^{2}}\int_{p,l}\delta_{k+l-p}\frac{{\left(k^{2}-2p\cdot k\right)}^{2}}{k^{2}}G^{12}\left(p\right)G^{21}\left(l\right), \end{array}$$

(A183) $$\begin{array}{ccc} \Pi_{L}^{\left(c\right),21}\left(k\right) & = & -\frac{e^{2}}{4m^{2}}\int_{p,l}\delta_{k+l-p}\frac{{\left(k^{2}-2p\cdot k\right)}^{2}}{k^{2}}G^{21}\left(p\right)G^{12}\left(l\right). \end{array}$$

The label (c) corresponds to diagram (c) in Figure 1. We find the convolution between Green’s functions for charged bosons $G^{12}\left(l\right)$ and photons $D_{T}^{12}\left(k\right)$ (and $D_{L}^{12}\left(k\right)$) in Equation (A178). The self-energy in Equations (A178) and (A179) represents the interaction between charged bosons and photons.

Differentiating Equation (A54) by Green’s functions and using the Fourier transformation, we can derive the self-energy for charged bosons,

(A184) $$\begin{array}{cc} \Sigma^{\left(d\right),12}\left(p\right)= & -4e^{2}\int_{k,l}\delta_{k+l-p}\left({\left(g_{aT}Q_{aT}+g_{oT}Q_{oT}\right)}^{2}-\frac{{\left(k\cdot (g_{aT}Q_{aT}+g_{oT}Q_{oT})\right)}^{2}}{k^{2}}\right)G^{12}\left(l\right)D_{T}^{12}\left(k\right), \end{array}$$

(A185) $$\begin{array}{cc} \Sigma^{\left(d\right),21}\left(p\right)= & -4e^{2}\int_{k,l}\delta_{k+l-p}\left({\left(g_{aT}Q_{aT}+g_{oT}Q_{oT}\right)}^{2}-\frac{{\left(k\cdot (g_{aT}Q_{aT}+g_{oT}Q_{oT})\right)}^{2}}{k^{2}}\right)G^{21}\left(l\right)D_{T}^{21}\left(k\right), \end{array}$$

the self-energy for transverse photons,

(A186) $$\begin{array}{cc} \Pi_{T}^{\left(d\right),12}\left(k\right) & =-2e^{2}\int_{p,l}\delta_{k+l-p}\left({\left(g_{aT}Q_{aT}+g_{oT}Q_{oT}\right)}^{2}-\frac{{\left(k\cdot (g_{aT}Q_{aT}+g_{oT}Q_{oT})\right)}^{2}}{k^{2}}\right)G^{12}\left(p\right)G^{21}\left(l\right), \end{array}$$

(A187) $$\begin{array}{cc} \Pi_{T}^{\left(d\right),21}\left(k\right) & =-2e^{2}\int_{p,l}\delta_{k+l-p}\left({\left(g_{aT}Q_{aT}+g_{oT}Q_{oT}\right)}^{2}-\frac{{\left(k\cdot (g_{aT}Q_{aT}+g_{oT}Q_{oT})\right)}^{2}}{k^{2}}\right)G^{21}\left(p\right)G^{12}\left(l\right). \end{array}$$

The label (d) corresponds to diagram (d) in Figure 1. We find the coupling between charged bosons and photons with coherent transverse acoustic and optical phonon fields $Q_{aT}=Q_{aT,j}$ and $Q_{oT}=Q_{oT,j}$ with j=1,2,3.

Differentiating Equation (A55) by Green’s functions as $\tilde{\Sigma }\left(x,y\right)=\frac{1}{2}\frac{\delta i\Gamma_{2}}{\delta \Delta (y,x)}$, and multiplying $e^{-ie\int_{y}^{x}dz_{\mu }A^{\mu }}$, we find,

(A188) $$\begin{array}{ccc} \Sigma^{\left(e\right)}\left(x,y\right) & = & -\frac{g_{aT}^{2}}{2}[\partial_{y,i}\left(\left(\partial_{x,j}G\left(x,y\right)\right)D_{aa,T}^{ij}\left(y,x\right)\right)+\partial_{y,j}\left(\left(\partial_{x,i}G\left(x,y\right)\right)D_{aa,T}^{ij}\left(x,y\right)\right)\\ & & \partial_{y,i}\partial_{x,j}\left(G\left(x,y\right)D_{aa,T}^{ij}\left(y,x\right)\right)+\left(\partial_{x,i}\partial_{y,j}G\left(x,y\right)\right)D_{aa,T}^{ij}\left(x,y\right)\\ & & \left(\partial_{y,i}\partial_{x,j}G\left(x,y\right)\right)D_{aa,T}^{ij}\left(y,x\right)+\partial_{x,i}\partial_{y,j}\left(G\left(x,y\right)D_{aa,T}^{ij}\left(x,y\right)\right)\\ & & \partial_{x,j}\left(\left(\partial_{y,i}G\left(x,y\right)\right)D_{aa,T}^{ij}\left(y,x\right)\right)+\partial_{x,i}\left(\left(\partial_{y,j}G\left(x,y\right)\right)D_{aa,T}^{ij}\left(x,y\right)\right)]+\left(a\to o\right), \end{array}$$

Fourier transforming by the relative coordinate x−y and using $D_{aa,T}^{ij}\left(k\right)=\left(\delta^{ij}-\frac{k^{i}k^{j}}{k^{2}}\right)D_{aa,T}\left(k\right)$, we arrive at the self-energy for charged bosons,

(A189) $$\begin{array}{ccc} \Sigma^{\left(e\right),12}\left(p\right) & = & -g_{aT}^{2}\int_{k,l}\delta_{k+l-p}\left(p_{i}+l_{i}\right)\left(p_{j}+l_{j}\right)\left(\delta_{ij}-\frac{k_{i}k_{j}}{k^{2}}\right)G^{12}\left(l\right)D_{aa,T}^{12}\left(k\right)\\ & & -g_{oT}^{2}\int_{k,l}\delta_{k+l-p}\left(p_{i}+l_{i}\right)\left(p_{j}+l_{j}\right)\left(\delta_{ij}-\frac{k_{i}k_{j}}{k^{2}}\right)G^{12}\left(l\right)D_{oo,T}^{12}\left(k\right)\\ & = & -4g_{aT}^{2}\int_{k,l}\delta_{k+l-p}\left(p^{2}-\frac{{\left(k\cdot p\right)}^{2}}{k^{2}}\right)G^{12}\left(l\right)D_{aa,T}^{12}\left(k\right)\\ & & -4g_{oT}^{2}\int_{k,l}\delta_{k+l-p}\left(p^{2}-\frac{{\left(k\cdot p\right)}^{2}}{k^{2}}\right)G^{12}\left(l\right)D_{oo,T}^{12}\left(k\right) \end{array}$$

(A190) $$\begin{array}{ccc} \Sigma^{\left(e\right),21}\left(p\right) & = & -4g_{aT}^{2}\int_{k,l}\delta_{k+l-p}\left(p^{2}-\frac{{\left(k\cdot p\right)}^{2}}{k^{2}}\right)G^{21}\left(l\right)D_{aa,T}^{21}\left(k\right),\\ & = & -4g_{oT}^{2}\int_{k,l}\delta_{k+l-p}\left(p^{2}-\frac{{\left(k\cdot p\right)}^{2}}{k^{2}}\right)G^{21}\left(l\right)D_{oo,T}^{21}\left(k\right). \end{array}$$

We also find the self-energy for transverse acoustic phonons,

(A191) $$\begin{array}{ccc} \Pi_{aa,T}^{\left(e\right),12}\left(k\right) & = & -2g_{aT}^{2}\int_{l,p}\delta_{k+l-p}\left(p^{2}-\frac{{\left(k\cdot p\right)}^{2}}{k^{2}}\right)G^{21}\left(l\right)G^{12}\left(p\right), \end{array}$$

(A192) $$\begin{array}{ccc} \Pi_{aa,T}^{\left(e\right),21}\left(k\right) & = & -2g_{aT}^{2}\int_{l,p}\delta_{k+l-p}\left(p^{2}-\frac{{\left(k\cdot p\right)}^{2}}{k^{2}}\right)G^{12}\left(l\right)G^{21}\left(p\right), \end{array}$$

and the self-energy for transverse optical phonons,

(A193) $$\begin{array}{ccc} \Pi_{oo,T}^{\left(e\right),12}\left(k\right) & = & -2g_{oT}^{2}\int_{l,p}\delta_{k+l-p}\left(p^{2}-\frac{{\left(k\cdot p\right)}^{2}}{k^{2}}\right)G^{21}\left(l\right)G^{12}\left(p\right), \end{array}$$

(A194) $$\begin{array}{ccc} \Pi_{oo,T}^{\left(e\right),21}\left(k\right) & = & -2g_{oT}^{2}\int_{l,p}\delta_{k+l-p}\left(p^{2}-\frac{{\left(k\cdot p\right)}^{2}}{k^{2}}\right)G^{12}\left(l\right)G^{21}\left(p\right). \end{array}$$

Here the label (e) represents the diagram (e) in Figure 1.

Differentiating Equation (A56) by Green’s functions, we find the self-energy for charged bosons,

(A195) $$\begin{array}{ccc} \Sigma^{\left(f\right),12}\left(p\right) & = & g_{aL}^{2}\int_{k,l}\delta_{k+l-p}k^{2}G^{12}\left(l\right)D_{aa,L}^{12}\left(k\right)+g_{oL}^{2}\int_{k,l}\delta_{k+l-p}k^{2}G^{12}\left(l\right)D_{oo,L}^{12}\left(k\right), \end{array}$$

(A196) $$\begin{array}{ccc} \Sigma^{\left(f\right),21}\left(p\right) & = & g_{aL}^{2}\int_{k,l}\delta_{k+l-p}k^{2}G^{21}\left(l\right)D_{aa,L}^{21}\left(k\right)+g_{oL}^{2}\int_{k,l}\delta_{k+l-p}k^{2}G^{21}\left(l\right)D_{oo,L}^{21}\left(k\right), \end{array}$$

and the self-energy for longitudinal acoustic and optical phonons,

(A197) $$\begin{array}{ccc} \Pi_{aa,L}^{\left(f\right),12}\left(k\right) & = & -g_{aL}^{2}\int_{p,l}\delta_{k+l-p}k^{2}G^{21}\left(l\right)G^{12}\left(p\right), \end{array}$$

(A198) $$\begin{array}{ccc} \Pi_{aa,L}^{\left(f\right),21}\left(k\right) & = & -g_{aL}^{2}\int_{p,l}\delta_{k+l-p}k^{2}G^{12}\left(l\right)G^{21}\left(p\right), \end{array}$$

(A199) $$\begin{array}{ccc} \Pi_{oo,L}^{\left(f\right),12}\left(k\right) & = & -g_{oL}^{2}\int_{p,l}\delta_{k+l-p}k^{2}G^{21}\left(l\right)G^{12}\left(p\right), \end{array}$$

(A200) $$\begin{array}{ccc} \Pi_{oo,L}^{\left(f\right),21}\left(k\right) & = & -g_{oL}^{2}\int_{p,l}\delta_{k+l-p}k^{2}G^{12}\left(l\right)G^{21}\left(p\right), \end{array}$$

The label (f) corresponds to diagram (f) in Figure 1.

Differentiating Equation (A57) by Green’s functions as $\tilde{\Sigma }\left(x,y\right)=\frac{1}{2}\frac{\delta i\Gamma_{2}}{\delta \Delta (y,x)}$ with $\Sigma \left(x,y\right)=e^{-ie\int_{y}^{x}dz^{\mu }A_{\mu }}\tilde{\Sigma }\left(x,y\right)$, and Fourier transforming with relative coordinate x−y, we find the self-energy for charged bosons,

(A201) $$\begin{array}{ccc} \Sigma^{\left(g\right),12}\left(p\right) & = & -\frac{e^{4}}{2m^{2}}{\left|\bar{\psi }\left(X\right)\right|}^{2}\int_{k,q}\delta_{p-eA-k-q}[\left(1+\frac{{\left(q\cdot k\right)}^{2}}{q^{2}k^{2}}\right)D_{T}^{12}\left(q\right)D_{T}^{12}\left(k\right)\\ & & +\left(1-\frac{{\left(q\cdot k\right)}^{2}}{q^{2}k^{2}}\right)\left(D_{T}^{12}\left(q\right)D_{L}^{12}\left(k\right)+D_{L}^{12}\left(q\right)D_{T}^{12}\left(k\right)\right)\\ & & +\frac{{\left(q\cdot k\right)}^{2}}{q^{2}k^{2}}D_{L}^{12}\left(q\right)D_{L}^{12}\left(k\right)], \end{array}$$

(A202) $$\begin{array}{ccc} \Sigma^{\left(g\right),21}\left(p\right) & = & -\frac{e^{4}}{2m^{2}}{\left|\bar{\psi }\left(X\right)\right|}^{2}\int_{k,q}\delta_{p-eA-k-q}[\left(1+\frac{{\left(q\cdot k\right)}^{2}}{q^{2}k^{2}}\right)D_{T}^{21}\left(q\right)D_{T}^{21}\left(k\right)\\ & & +\left(1-\frac{{\left(q\cdot k\right)}^{2}}{q^{2}k^{2}}\right)\left(D_{T}^{21}\left(q\right)D_{L}^{21}\left(k\right)+D_{L}^{21}\left(q\right)D_{T}^{21}\left(k\right)\right)\\ & & +\frac{{\left(q\cdot k\right)}^{2}}{q^{2}k^{2}}D_{L}^{21}\left(q\right)D_{L}^{21}\left(k\right)], \end{array}$$

with eA representing $e(A^{\mu }-\partial^{\mu }\beta /e)$ appearing in self-energy as the factor,

(A203) $$\begin{array}{c} {\bar{\psi }}^{*}\left(y\right)e^{-ie\int_{y}^{x}dzA}\bar{\psi }\left(x\right)∼{\left|\bar{\psi }\left(X\right)\right|}^{2}e^{-ie\left(A^{\mu }\left(X\right)-\partial_{X}^{\mu }\beta /e\right)\left(x-y\right)}, \end{array}$$

with $\bar{\psi }\left(x\right)=e^{i\beta (x)}\left|\bar{\psi }\left(x\right)\right|$ and ${\bar{\psi }}^{*}\left(y\right)=e^{-i\beta (y)}\left|\bar{\psi }\left(y\right)\right|$ involving the phase β(x) of $\bar{\psi }\left(x\right)$, and we can derive the self-energy for transverse photons,

(A204) $$\begin{array}{ccc} \Pi_{T}^{\left(g\right),12}\left(k\right) & = & -\frac{e^{4}}{2m^{2}}{\left|\bar{\psi }\right|}^{2}\int_{p,q}\delta_{k-q-p}[\left(1+\frac{{\left(q\cdot k\right)}^{2}}{q^{2}k^{2}}\right)D_{T}^{12}\left(q\right)\left(G^{12}\left(p+eA\right)+G^{21}\left(-p+eA\right)\right)\\ & & +\left(1-\frac{{\left(q\cdot k\right)}^{2}}{q^{2}k^{2}}\right)D_{L}^{12}\left(q\right)\left(G^{12}\left(p+eA\right)+G^{21}\left(-p+eA\right)\right)], \end{array}$$

(A205) $$\begin{array}{ccc} \Pi_{T}^{\left(g\right),21}\left(k\right) & = & -\frac{e^{4}}{2m^{2}}{\left|\bar{\psi }\right|}^{2}\int_{p,q}\delta_{k-q-p}[\left(1+\frac{{\left(q\cdot k\right)}^{2}}{q^{2}k^{2}}\right)D_{T}^{21}\left(q\right)\left(G^{21}\left(p+eA\right)+G^{12}\left(-p+eA\right)\right)\\ & & +\left(1-\frac{{\left(q\cdot k\right)}^{2}}{q^{2}k^{2}}\right)D_{L}^{21}\left(q\right)\left(G^{21}\left(p+eA\right)+G^{12}\left(-p+eA\right)\right)], \end{array}$$

the self-energy for longitudinal photons,

(A206) $$\begin{array}{ccc} \Pi_{L}^{\left(g\right),12}\left(k\right) & = & -\frac{e^{4}}{m^{2}}{\left|\bar{\psi }\right|}^{2}\int_{p,q}\delta_{k-q-p}[\left(1-\frac{{\left(q\cdot k\right)}^{2}}{q^{2}k^{2}}\right)D_{T}^{12}\left(q\right)\left(G^{12}\left(p+eA\right)+G^{21}\left(-p+eA\right)\right)\\ & & +\frac{{\left(q\cdot k\right)}^{2}}{q^{2}k^{2}}D_{L}^{12}\left(q\right)\left(G^{12}\left(p+eA\right)+G^{21}\left(-p+eA\right)\right)], \end{array}$$

(A207) $$\begin{array}{ccc} \Pi_{L}^{\left(g\right),21}\left(k\right) & = & -\frac{e^{4}}{m^{2}}{\left|\bar{\psi }\right|}^{2}\int_{p,q}\delta_{k-q-p}[\left(1-\frac{{\left(q\cdot k\right)}^{2}}{q^{2}k^{2}}\right)D_{T}^{21}\left(q\right)\left(G^{21}\left(p+eA\right)+G^{12}\left(-p+eA\right)\right)\\ & & +\frac{{\left(q\cdot k\right)}^{2}}{q^{2}k^{2}}D_{L}^{21}\left(q\right)\left(G^{21}\left(p+eA\right)+G^{12}\left(-p+eA\right)\right)]. \end{array}$$

The label (g) represents the diagram (g) in Figure 1. We find the convolutional integral for Green’s functions for charged bosons with momenta *p* shifted by ±eA and Green’s functions for photons. The self-energy labeled by (g) represents field-particle conversion processes where coherent charged boson fields coupled with incoherent photons decay into incoherent charged bosons.

Similarly, differentiating Equation (A58) by Green’s functions, we can derive self-energy for charged bosons,

(A208) $$\begin{array}{ccc} \Sigma^{\left(h\right),12}\left(p\right) & = & -4{\left(eg_{aT}\right)}^{2}{\left|\bar{\psi }\left(X\right)\right|}^{2}\int_{k,q}\delta_{p-eA-k-q}\left(1+\frac{{\left(q\cdot k\right)}^{2}}{q^{2}k^{2}}\right)D_{T}^{12}\left(q\right)D_{aa,T}^{12}\left(k\right)\\ & & -4{\left(eg_{oT}\right)}^{2}{\left|\bar{\psi }\left(X\right)\right|}^{2}\int_{k,q}\delta_{p-eA-k-q}\left(1+\frac{{\left(q\cdot k\right)}^{2}}{q^{2}k^{2}}\right)D_{T}^{12}\left(q\right)D_{oo,T}^{12}\left(k\right), \end{array}$$

(A209) $$\begin{array}{ccc} \Sigma^{\left(h\right),21}\left(p\right) & = & -4{\left(eg_{aT}\right)}^{2}{\left|\bar{\psi }\left(X\right)\right|}^{2}\int_{k,q}\delta_{p-eA-k-q}\left(1+\frac{{\left(q\cdot k\right)}^{2}}{q^{2}k^{2}}\right)D_{T}^{21}\left(q\right)D_{aa,T}^{21}\left(k\right)\\ & & 4{\left(eg_{oT}\right)}^{2}{\left|\bar{\psi }\left(X\right)\right|}^{2}\int_{k,q}\delta_{p-eA-k-q}\left(1+\frac{{\left(q\cdot k\right)}^{2}}{q^{2}k^{2}}\right)D_{T}^{21}\left(q\right)D_{oo,T}^{21}\left(k\right), \end{array}$$

the self-energy for transverse photons,

(A210) $$\begin{array}{ccc} \Pi_{T}^{\left(h\right),12}\left(k\right) & = & -2{\left(eg_{aT}\right)}^{2}{\left|\bar{\psi }\right|}^{2}\int_{p,q}\delta_{k-q-p}\left(1+\frac{{\left(q\cdot k\right)}^{2}}{q^{2}k^{2}}\right)D_{aa,T}^{12}\left(q\right)\left(G^{12}\left(p+eA\right)+G^{21}\left(-p+eA\right)\right)\\ & & -2{\left(eg_{oT}\right)}^{2}{\left|\bar{\psi }\right|}^{2}\int_{p,q}\delta_{k-q-p}\left(1+\frac{{\left(q\cdot k\right)}^{2}}{q^{2}k^{2}}\right)D_{oo,T}^{12}\left(q\right)\left(G^{12}\left(p+eA\right)+G^{21}\left(-p+eA\right)\right), \end{array}$$

(A211) $$\begin{array}{ccc} \Pi_{T}^{\left(h\right),21}\left(k\right) & = & -2{\left(eg_{aT}\right)}^{2}{\left|\bar{\psi }\right|}^{2}\int_{p,q}\delta_{k-q-p}\left(1+\frac{{\left(q\cdot k\right)}^{2}}{q^{2}k^{2}}\right)D_{aa,T}^{21}\left(q\right)\left(G^{21}\left(p+eA\right)+G^{12}\left(-p+eA\right)\right)\\ & & -2{\left(eg_{oT}\right)}^{2}{\left|\bar{\psi }\right|}^{2}\int_{p,q}\delta_{k-q-p}\left(1+\frac{{\left(q\cdot k\right)}^{2}}{q^{2}k^{2}}\right)D_{oo,T}^{21}\left(q\right)\left(G^{21}\left(p+eA\right)+G^{12}\left(-p+eA\right)\right), \end{array}$$

the self-energy for transverse acoustic phonons,

(A212) $$\begin{array}{ccc} \Pi_{aa,T}^{\left(h\right),12}\left(k\right) & = & -2{\left(eg_{aT}\right)}^{2}{\left|\bar{\psi }\right|}^{2}\int_{p,q}\delta_{k-q-p}\left(1+\frac{{\left(q\cdot k\right)}^{2}}{q^{2}k^{2}}\right)D_{T}^{12}\left(q\right)\left(G^{12}\left(p+eA\right)+G^{21}\left(-p+eA\right)\right), \end{array}$$

(A213) $$\begin{array}{ccc} \Pi_{aa,T}^{\left(h\right),21}\left(k\right) & = & -2{\left(eg_{aT}\right)}^{2}{\left|\bar{\psi }\right|}^{2}\int_{p,q}\delta_{k-q-p}\left(1+\frac{{\left(q\cdot k\right)}^{2}}{q^{2}k^{2}}\right)D_{T}^{21}\left(q\right)\left(G^{21}\left(p+eA\right)+G^{12}\left(-p+eA\right)\right), \end{array}$$

and the self-energy for transverse optical phonons,

(A214) $$\begin{array}{ccc} \Pi_{oo,T}^{\left(h\right),12}\left(k\right) & = & -2{\left(eg_{oT}\right)}^{2}{\left|\bar{\psi }\right|}^{2}\int_{p,q}\delta_{k-q-p}\left(1+\frac{{\left(q\cdot k\right)}^{2}}{q^{2}k^{2}}\right)D_{T}^{12}\left(q\right)\left(G^{12}\left(p+eA\right)+G^{21}\left(-p+eA\right)\right), \end{array}$$

(A215) $$\begin{array}{ccc} \Pi_{oo,T}^{\left(h\right),21}\left(k\right) & = & -2{\left(eg_{oT}\right)}^{2}{\left|\bar{\psi }\right|}^{2}\int_{p,q}\delta_{k-q-p}\left(1+\frac{{\left(q\cdot k\right)}^{2}}{q^{2}k^{2}}\right)D_{T}^{21}\left(q\right)\left(G^{21}\left(p+eA\right)+G^{12}\left(-p+eA\right)\right). \end{array}$$

The label (h) corresponds to diagram (h) in Figure 1. The self-energy labeled by (h) represents field-particle conversion processes where coherent charged boson field coupled with photons and phonons decay into incoherent charged bosons.

Finally taking the sum of Equations (A133), (A138), (A139), (A145), (A146), (A152) and (A153), defining $s^{\mu }\left(X\right)$ as,

(A216) $$\begin{array}{c} s^{\mu }=s_{cb}^{\mu }+s_{\mathrm{photon},L}^{\mu }+s_{\mathrm{photon},T}^{\mu }+s_{a,L}^{\mu }+s_{a,T}^{\mu }+s_{o,L}^{\mu }+s_{o,T}^{\mu }, \end{array}$$

and using self-energy we arrive at,

(A217) $$\begin{array}{c} \partial_{\mu }s^{\mu }=\left(\mathrm{PP}\right)+\left(\mathrm{Dia}(c)\right)+\left(\mathrm{Dia}(d)\right)+\left(\mathrm{Dia}(e)\right)+\left(\mathrm{Dia}(f)\right)+\left(\mathrm{Dia}(g)\right)+\left(\mathrm{Dia}(h)\right)\geq 0, \end{array}$$

We can express,

(A218) $$\begin{array}{ccc} \left(\mathrm{PP}\right) & = & 4{\left(eg_{aT}\right)}^{2}{\left(|\bar{\psi }{\left(X\right)|}^{2}+\int_{p}F\right)}^{2}\int_{k}\left(D_{T}^{12}d_{aa,T}^{21}-D_{T}^{21}d_{aa,T}^{12}\right)\ln\frac{D_{T}^{12}d_{aa,T}^{21}}{D_{T}^{21}d_{aa,T}^{12}}\\ & & +4{\left(eg_{oT}\right)}^{2}{\left(|\bar{\psi }{\left(X\right)|}^{2}+\int_{p}F\right)}^{2}\int_{k}\left(D_{T}^{12}d_{oo,T}^{21}-D_{T}^{21}d_{oo,T}^{12}\right)\ln\frac{D_{T}^{12}d_{oo,T}^{21}}{D_{T}^{21}d_{oo,T}^{12}}\geq 0, \end{array}$$

since $\left(x-y\right)\ln\frac{x}{y}\geq 0$ in x,y>0. Here we have used $\ln\frac{D_{aa,T}^{21}}{D_{aa,T}^{12}}=\ln\frac{\left(1+f_{aa,T}\right)}{f_{aa,T}}∼\ln\frac{1+\gamma_{d,aa,T}}{\gamma_{d,aa,T}}=\ln\frac{d_{aa,T}^{21}}{d_{aa,T}^{12}}$ with Equations (A143) and (A144) in 1st order approximation in the gradient expansion. The remaining terms in Equation (A217) are,

(A219) $$\begin{array}{ccc} \;\;\;\;\;\;(\mathrm{Dia}(c)) & = & \frac{e^{2}}{8m^{2}}\int_{l,k,p}\delta_{l+k-p}\frac{{\left(k^{2}-2k\cdot p\right)}^{2}}{k^{2}}\left(G^{12}\left(p\right)G^{21}\left(l\right)D_{L}^{21}\left(k\right)-G^{21}\left(p\right)G^{12}\left(l\right)D_{L}^{12}\left(k\right)\right)\\ & & \times \ln\frac{G^{12}\left(p\right)G^{21}\left(l\right)D_{L}^{21}\left(k\right)}{G^{21}\left(p\right)G^{12}\left(l\right)D_{L}^{12}\left(k\right)}\\ & & +\frac{e^{2}}{8m^{2}}\int_{k,l,p}\delta_{k+l-p}4\left(p^{2}-\frac{{\left(p\cdot k\right)}^{2}}{k^{2}}\right)\left(G^{12}\left(p\right)G^{21}\left(l\right)D_{T}^{21}\left(k\right)-G^{21}\left(p\right)G^{12}\left(l\right)D_{T}^{12}\left(k\right)\right)\\ & & \times \ln\frac{G^{12}\left(p\right)G^{21}\left(l\right)D_{T}^{21}\left(k\right)}{G^{21}\left(p\right)G^{12}\left(l\right)D_{T}^{12}\left(k\right)}\\ & \geq & 0, \end{array}$$

(A220) $$\begin{array}{ccc} \;\;\;(\mathrm{Dia}(d)) & = & 2e^{2}\int_{k,l,p}\delta_{k+l-p}\left({\left(g_{aT}Q_{aT}+g_{oT}Q_{oT}\right)}^{2}-\frac{{\left(k\cdot (g_{aT}Q_{aT}+g_{oT}Q_{oT})\right)}^{2}}{k^{2}}\right)\\ & & \times \left(G^{12}\left(p\right)G^{21}\left(l\right)D_{T}^{21}\left(k\right)-G^{21}\left(p\right)G^{12}\left(l\right)D_{T}^{12}\left(k\right)\right)\ln\frac{G^{12}\left(p\right)G^{21}\left(l\right)D_{T}^{21}\left(k\right)}{G^{21}\left(p\right)G^{12}\left(l\right)D_{T}^{12}\left(k\right)}\\ & \geq & 0, \end{array}$$

(A221) $$\begin{array}{ccc} \;\;\;\;\;\;\;\;(\mathrm{Dia}(e)) & = & 2g_{aT}^{2}\int_{k,l,p}\delta_{k+l-p}\left(p^{2}-\frac{{\left(k\cdot p\right)}^{2}}{k^{2}}\right)\\ & & \times \left(G^{12}\left(p\right)G^{21}\left(l\right)D_{aa,T}^{21}\left(k\right)-G^{21}\left(p\right)G^{12}\left(l\right)D_{aa,T}^{12}\left(k\right)\right)\ln\frac{G^{12}\left(p\right)G^{21}\left(l\right)D_{aa,T}^{21}\left(k\right)}{G^{21}\left(p\right)G^{12}\left(l\right)D_{aa,T}^{12}\left(k\right)}+\left(a\to o\right)\\ & \geq & 0, \end{array}$$

(A222) $$\begin{array}{ccc} \left(\mathrm{Dia}(f)\right) & = & \frac{g_{aL}^{2}}{2}\int_{k,l,p}\delta_{k+l-p}k^{2}\left(G^{12}\left(p\right)G^{21}\left(l\right)D_{aa,L}^{21}\left(k\right)-G^{21}\left(p\right)G^{12}\left(l\right)D_{aa,L}^{12}\left(k\right)\right)\\ & & \times \ln\frac{G^{12}\left(p\right)G^{21}\left(l\right)D_{aa,L}^{21}\left(k\right)}{G^{21}\left(p\right)G^{12}\left(l\right)D_{aa,L}^{12}\left(k\right)}+\left(a\to o\right)\\ & \geq & 0, \end{array}$$

(A223) $$\begin{array}{ccc} \;\;\;\;(\mathrm{Dia}(g)) & = & \frac{e^{4}}{2m}{\left|\bar{\psi }\right|}^{2}\int_{p,k,q}\delta_{p-eA-k-q}\left(1+\frac{{\left(q\cdot k\right)}^{2}}{q^{2}k^{2}}\right)\\ & & \times \left(D_{T}^{12}\left(k\right)D_{T}^{12}\left(q\right)G^{21}\left(p\right)-D_{T}^{21}\left(k\right)D_{T}^{21}\left(q\right)G^{12}\left(p\right)\right)\ln\frac{D_{T}^{12}\left(k\right)D_{T}^{12}\left(q\right)G^{21}\left(p\right)}{D_{T}^{21}\left(k\right)D_{T}^{21}\left(q\right)G^{12}\left(p\right)}\\ & & +\frac{e^{4}}{m}{\left|\bar{\psi }\right|}^{2}\int_{p,k,q}\delta_{p-eA-k-q}\left(1-\frac{{\left(q\cdot k\right)}^{2}}{q^{2}k^{2}}\right)\\ & & \times \left(D_{L}^{12}\left(k\right)D_{T}^{12}\left(q\right)G^{21}\left(p\right)-D_{L}^{21}\left(k\right)D_{T}^{21}\left(q\right)G^{12}\left(p\right)\right)\ln\frac{D_{L}^{12}\left(k\right)D_{T}^{12}\left(q\right)G^{21}\left(p\right)}{D_{L}^{21}\left(k\right)D_{T}^{21}\left(q\right)G^{12}\left(p\right)}\\ & & +\frac{e^{4}}{2m}{\left|\bar{\psi }\right|}^{2}\int_{p,k,q}\delta_{p-eA-k-q}\frac{{\left(q\cdot k\right)}^{2}}{q^{2}k^{2}}\\ & & \times \left(D_{L}^{12}\left(k\right)D_{L}^{12}\left(q\right)G^{21}\left(p\right)-D_{L}^{21}\left(k\right)D_{L}^{21}\left(q\right)G^{12}\left(p\right)\right)\ln\frac{D_{L}^{12}\left(k\right)D_{L}^{12}\left(q\right)G^{21}\left(p\right)}{D_{L}^{21}\left(k\right)D_{L}^{21}\left(q\right)G^{12}\left(p\right)}\\ & \geq & 0, \end{array}$$

(A224) $$\begin{array}{ccc} \;\;\;\;\;(\mathrm{Dia}(h)) & = & 4{\left(eg_{aT}\right)}^{2}{\left|\bar{\psi }\right|}^{2}\int_{p,k,q}\delta_{p-eA-k-q}\left(1+\frac{{\left(q\cdot k\right)}^{2}}{q^{2}k^{2}}\right)\\ & & \times \left(D_{T}^{12}\left(k\right)D_{aa,T}^{12}\left(q\right)G^{21}\left(p\right)-D_{T}^{21}\left(k\right)D_{aa,T}^{21}\left(q\right)G^{12}\left(p\right)\right)\ln\frac{D_{T}^{12}\left(k\right)D_{aa,T}^{12}\left(q\right)G^{21}\left(p\right)}{D_{T}^{21}\left(k\right)D_{aa,T}^{21}\left(q\right)G^{12}\left(p\right)}\\ & & +(a\to o)\\ & \geq & 0, \end{array}$$

where we have used $D_{T}^{12}\left(k\right)=D_{T}^{21}\left(-k\right)$, $D_{L}^{12}\left(k\right)=D_{L}^{21}\left(-k\right)$, $D_{aa,T}^{12}\left(k\right)=D_{aa,T}^{21}\left(-k\right)$, $D_{aa,L}^{12}\left(k\right)=D_{aa,L}^{21}\left(-k\right)$, $D_{oo,T}^{12}\left(k\right)=D_{oo,T}^{21}\left(-k\right)$, and $D_{oo,L}^{12}\left(k\right)=D_{oo,L}^{21}\left(-k\right)$, and (a→o) represents changing subscript ‘*a*’ (acoustic) to ‘*o*’ (optical) in the previous term. We have proved the H-theorem in Hartree–Fock approximation for Kadanoff–Baym equations in 1st order in the gradient expansions. Entropy production in Equation (A217) stops when,

(A225) $$\begin{array}{ccc} f(p) & = & \frac{1}{e^{\frac{p^{0}-\mu_{c}}{T}}-1}, \end{array}$$

with temperature *T* and chemical potential $\mu_{c}$, and,

(A226) $$\begin{array}{c} f_{T}\left(k\right)=f_{L}\left(k\right)=f_{aa,T}\left(k\right)=f_{aa,L}\left(k\right)=f_{oo,T}\left(k\right)=f_{oo,L}\left(k\right)=\frac{1}{e^{\frac{k^{0}}{T}}-1}, \end{array}$$

are satisfied. In addition, due to Equations (A223) and (A224), the chemical potential $\mu_{c}$ is,

(A227) $$\begin{array}{c} \mu_{c}=e\left(A^{0}-\frac{\partial^{0}\beta }{e}\right). \end{array}$$

## Appendix E. Self-Energy in Kadanoff–Baym Equations

We write local self-energy and statistical and spectral parts in nonlocal self-energy for Kadanoff–Baym equations.

The local self-energy for charged bosons is given by,

(A228) Σloc(X)=e22m∫k2FT(X,k)+FL(X,k)−8(egaT)2∫kRedaa,R,T(X,k)FT(X,k)+daa,F,T(X,k)ReDR,T(X,k)−8(egoT)2∫kRedoo,R,T(X,k)FT(X,k)+doo,F,T(X,k)ReDR,T(X,k),

for diagram (a) and (b) in Figure 1 where we have used,

(A229) egaT(Dγa,F,T,ii+Daγ,F,T,ii)=−8(egaT)2∫kRedaa,R,TFT+daa,F,TReDR,T,

(A230) egoT(Dγo,F,T,ii+Doγ,F,T,ii)=−8(egoT)2∫kRedoo,R,TFT+doo,F,TReDR,T.

Statistical part and spectral part in nonlocal self-energy for charged bosons are,

(A231) $$\begin{array}{ccc} \Sigma_{F}\left(p\right) & = & \Sigma_{F}^{\left(c\right)}\left(p\right)+\Sigma_{F}^{\left(d\right)}\left(p\right)+\Sigma_{F}^{\left(e\right)}\left(p\right)+\Sigma_{F}^{\left(f\right)}\left(p\right)+\Sigma_{F}^{\left(g\right)}\left(p\right)+\Sigma_{F}^{\left(h\right)}\left(p\right), \end{array}$$

(A232) $$\begin{array}{ccc} \Sigma_{\rho }\left(p\right) & = & \Sigma_{\rho }^{\left(c\right)}\left(p\right)+\Sigma_{\rho }^{\left(d\right)}\left(p\right)+\Sigma_{\rho }^{\left(e\right)}\left(p\right)+\Sigma_{\rho }^{\left(f\right)}\left(p\right)+\Sigma_{\rho }^{\left(g\right)}\left(p\right)+\Sigma_{\rho }^{\left(h\right)}\left(p\right), \end{array}$$

with the self-energy for diagram (c) in Figure 1,

(A233) $$\begin{array}{ccc} \;\;\Sigma_{F}^{\left(c\right)}\left(p\right) & = & -\frac{e^{2}}{4m^{2}}\int_{k}[4\left(p^{2}-\frac{{\left(k\cdot p\right)}^{2}}{k^{2}}\right)\left(F\left(p-k\right)F_{T}\left(k\right)+\frac{1}{4}\frac{\rho (p-k)}{i}\frac{\rho_{T}\left(k\right)}{i}\right)\\ & & +\frac{{\left(k^{2}-2p\cdot k\right)}^{2}}{k^{2}}\left(F\left(p-k\right)F_{L}\left(k\right)+\frac{1}{4}\frac{\rho (p-k)}{i}\frac{\rho_{L}\left(k\right)}{i}\right)], \end{array}$$

(A234) $$\begin{array}{ccc} \Sigma_{\rho }^{\left(c\right)}\left(p\right) & = & -\frac{e^{2}}{4m^{2}}\int_{k}[4\left(p^{2}-\frac{{\left(k\cdot p\right)}^{2}}{k^{2}}\right)\left(\rho \left(p-k\right)F_{T}\left(k\right)+F\left(p-k\right)\rho_{T}\left(k\right)\right)\\ & & +\frac{{\left(k^{2}-2p\cdot k\right)}^{2}}{k^{2}}\left(\rho \left(p-k\right)F_{L}\left(k\right)+F\left(p-k\right)\rho_{L}\left(k\right)\right)], \end{array}$$

the self-energy for diagram (d) in Figure 1,

(A235) $$\begin{array}{ccc} \Sigma_{F}^{\left(d\right)}\left(p\right) & = & -\frac{4e^{2}}{m^{2}}\int_{k}\left({\tilde{Q}}^{2}-\frac{{\left(\tilde{Q}\cdot k\right)}^{2}}{k^{2}}\right)\left(F\left(p-k\right)F_{T}\left(k\right)+\frac{1}{4}\frac{\rho (p-k)}{i}\frac{\rho_{T}\left(k\right)}{i}\right), \end{array}$$

(A236) $$\begin{array}{ccc} \Sigma_{\rho }^{\left(d\right)}\left(p\right) & = & -\frac{4e^{2}}{m^{2}}\int_{k}\left({\tilde{Q}}^{2}-\frac{{\left(\tilde{Q}\cdot k\right)}^{2}}{k^{2}}\right)\left(\rho \left(p-k\right)F_{T}\left(k\right)+F\left(p-k\right)\rho_{T}\left(k\right)\right), \end{array}$$

with definition ${\tilde{Q}}_{i}=m\left(g_{aT}{\bar{Q}}_{aT,i}+g_{oT}{\bar{Q}}_{oT,i}\right)$, the self-energy for diagram (e) in Figure 1,

(A237) $$\begin{array}{ccc} \;\;\Sigma_{F}^{\left(e\right)}\left(p\right) & = & -4g_{aT}^{2}\int_{k}\left(p^{2}-\frac{{\left(p\cdot k\right)}^{2}}{k^{2}}\right)\left(F\left(p-k\right)F_{aa,T}\left(k\right)+\frac{1}{4}\frac{\rho (p-k)}{i}\frac{\rho_{aa,T}\left(k\right)}{i}\right)\\ & & -4g_{oT}^{2}\int_{k}\left(p^{2}-\frac{{\left(p\cdot k\right)}^{2}}{k^{2}}\right)\left(F\left(p-k\right)F_{oo,T}\left(k\right)+\frac{1}{4}\frac{\rho (p-k)}{i}\frac{\rho_{oo,T}\left(k\right)}{i}\right), \end{array}$$

(A238) $$\begin{array}{ccc} \Sigma_{\rho }^{\left(e\right)}\left(p\right) & = & -4g_{aT}^{2}\int_{k}\left(p^{2}-\frac{{\left(p\cdot k\right)}^{2}}{k^{2}}\right)\left(\rho \left(p-k\right)F_{aa,T}\left(k\right)+F\left(p-k\right)\rho_{aa,T}\left(k\right)\right)\\ & & -4g_{oT}^{2}\int_{k}\left(p^{2}-\frac{{\left(p\cdot k\right)}^{2}}{k^{2}}\right)\left(\rho \left(p-k\right)F_{oo,T}\left(k\right)+F\left(p-k\right)\rho_{oo,T}\left(k\right)\right), \end{array}$$

the self-energy for diagram (f) in Figure 1,

(A239) $$\begin{array}{ccc} \;\;\Sigma_{F}^{\left(f\right)}\left(p\right) & = & -g_{aL}^{2}\int_{k}\;k^{2}\left(F\left(p-k\right)F_{aa,L}\left(k\right)+\frac{1}{4}\frac{\rho (p-k)}{i}\frac{\rho_{aa,L}\left(k\right)}{i}\right)\\ & & -g_{oL}^{2}\int_{k}\;k^{2}\left(F\left(p-k\right)F_{oo,L}\left(k\right)+\frac{1}{4}\frac{\rho (p-k)}{i}\frac{\rho_{oo,L}\left(k\right)}{i}\right), \end{array}$$

(A240) $$\begin{array}{ccc} \Sigma_{\rho }^{\left(e\right)}\left(p\right) & = & g_{aL}^{2}\int_{k}\;k^{2}\left(\rho \left(p-k\right)F_{aa,L}\left(k\right)+F\left(p-k\right)\rho_{aa,L}\left(k\right)\right)\\ & & -g_{oL}^{2}\int_{k}\;k^{2}\left(\rho \left(p-k\right)F_{oo,L}\left(k\right)+F\left(p-k\right)\rho_{oo,L}\left(k\right)\right), \end{array}$$

the self-energy for diagram (g) in Figure 1,

(A241) $$\begin{array}{ccc} \;\;\Sigma_{F}^{\left(g\right)}\left(p+eA\right) & = & -\frac{e^{4}}{2m^{2}}{\left|\bar{\psi }\right|}^{2}\int_{k}[\left(1+\frac{{\left((p-k)\cdot k\right)}^{2}}{{\left(p-k\right)}^{2}k^{2}}\right)\left(F_{T}\left(p-k\right)F_{T}\left(k\right)+\frac{1}{4}\frac{\rho_{T}\left(p-k\right)}{i}\frac{\rho_{T}\left(k\right)}{i}\right)\\ & & +\left(1-\frac{{\left((p-k)\cdot k\right)}^{2}}{{\left(p-k\right)}^{2}k^{2}}\right)\left(F_{T}\left(p-k\right)F_{L}\left(k\right)+\frac{1}{4}\frac{\rho_{T}\left(p-k\right)}{i}\frac{\rho_{L}\left(k\right)}{i}\right)\\ & & +\left(1-\frac{{\left((p-k)\cdot k\right)}^{2}}{{\left(p-k\right)}^{2}k^{2}}\right)\left(F_{L}\left(p-k\right)F_{T}\left(k\right)+\frac{1}{4}\frac{\rho_{L}\left(p-k\right)}{i}\frac{\rho_{T}\left(k\right)}{i}\right)\\ & & +\frac{{\left((p-k)\cdot k\right)}^{2}}{{\left(p-k\right)}^{2}k^{2}}\left(F_{L}\left(p-k\right)F_{L}\left(k\right)+\frac{1}{4}\frac{\rho_{L}\left(p-k\right)}{i}\frac{\rho_{L}\left(k\right)}{i}\right)], \end{array}$$

(A242) $$\begin{array}{ccc} \Sigma_{\rho }^{\left(g\right)}\left(p+eA\right) & = & -\frac{e^{4}}{2m^{2}}{\left|\bar{\psi }\right|}^{2}\int_{k}[\left(1+\frac{{\left((p-k)\cdot k\right)}^{2}}{{\left(p-k\right)}^{2}k^{2}}\right)\left(\rho_{T}\left(p-k\right)F_{T}\left(k\right)+F_{T}\left(p-k\right)\rho_{T}\left(k\right)\right)\\ & & +\left(1-\frac{{\left((p-k)\cdot k\right)}^{2}}{{\left(p-k\right)}^{2}k^{2}}\right)\left(\rho_{T}\left(p-k\right)F_{L}\left(k\right)+F_{T}\left(p-k\right)\rho_{L}\left(k\right)\right)\\ & & +\left(1-\frac{{\left((p-k)\cdot k\right)}^{2}}{{\left(p-k\right)}^{2}k^{2}}\right)\left(\rho_{L}\left(p-k\right)F_{T}\left(k\right)+F_{L}\left(p-k\right)\rho_{T}\left(k\right)\right)\\ & & +\frac{{\left((p-k)\cdot k\right)}^{2}}{{\left(p-k\right)}^{2}k^{2}}\left(\rho_{L}\left(p-k\right)F_{L}\left(k\right)+F_{L}\left(p-k\right)\rho_{L}\left(k\right)\right)], \end{array}$$

the self-energy for diagram (h) in Figure 1,

(A243) $$\begin{array}{cc} \;\;\Sigma_{F}^{\left(h\right)}\left(p+eA\right)= & -4{\left(eg_{aT}\right)}^{2}{\left|\bar{\psi }\right|}^{2}\int_{k}\left[\left(1+\frac{{\left((p-k)\cdot k\right)}^{2}}{{\left(p-k\right)}^{2}k^{2}}\right)\left(F_{T}\left(p-k\right)F_{aa,T}\left(k\right)+\frac{1}{4}\frac{\rho_{T}\left(p-k\right)}{i}\frac{\rho_{aa,T}\left(k\right)}{i}\right)\right]\\ & -4{\left(eg_{oT}\right)}^{2}{\left|\bar{\psi }\right|}^{2}\int_{k}\left[\left(1+\frac{{\left((p-k)\cdot k\right)}^{2}}{{\left(p-k\right)}^{2}k^{2}}\right)\left(F_{T}\left(p-k\right)F_{oo,T}\left(k\right)+\frac{1}{4}\frac{\rho_{T}\left(p-k\right)}{i}\frac{\rho_{oo,T}\left(k\right)}{i}\right)\right], \end{array}$$

(A244) $$\begin{array}{cc} \Sigma_{\rho }^{\left(h\right)}\left(p+eA\right)= & -4{\left(eg_{aT}\right)}^{2}{\left|\bar{\psi }\right|}^{2}\int_{k}\left[\left(1+\frac{{\left((p-k)\cdot k\right)}^{2}}{{\left(p-k\right)}^{2}k^{2}}\right)\left(\rho_{T}\left(p-k\right)F_{aa,T}\left(k\right)+F_{T}\left(p-k\right)\rho_{aa,T}\left(k\right)\right)\right]\\ & -4{\left(eg_{oT}\right)}^{2}{\left|\bar{\psi }\right|}^{2}\int_{k}\left[\left(1+\frac{{\left((p-k)\cdot k\right)}^{2}}{{\left(p-k\right)}^{2}k^{2}}\right)\left(\rho_{T}\left(p-k\right)F_{oo,T}\left(k\right)+F_{T}\left(p-k\right)\rho_{oo,T}\left(k\right)\right)\right]. \end{array}$$

Next we write local self-energy for photons. It is written by,

(A245) $$\begin{array}{ccc} \Pi_{\mathrm{loc}}\left(X\right) & = & \frac{e^{2}}{m}\int_{p}F\left(X,p\right). \end{array}$$

In Kadanoff–Baym equations for photons and phonons (21), (22), (25) and (26), we use,

(A246) $$\begin{array}{ccc} U_{aa,F,T}\left(X,k\right) & = & 4{\left(eg_{aT}\right)}^{2}{\left(|\bar{\psi }{\left(X\right)|}^{2}+\int_{p}F\left(X,p\right)\right)}^{2}d_{aa,F,T}\left(X,k\right), \end{array}$$

(A247) $$\begin{array}{ccc} U_{aa,\rho ,T}\left(X,k\right) & = & 4{\left(eg_{aT}\right)}^{2}{\left(|\bar{\psi }{\left(X\right)|}^{2}+\int_{p}F\left(X,p\right)\right)}^{2}d_{aa,\rho ,T}\left(X,k\right), \end{array}$$

(A248) $$\begin{array}{ccc} U_{oo,F,T}\left(X,k\right) & = & 4{\left(eg_{oT}\right)}^{2}{\left(|\bar{\psi }{\left(X\right)|}^{2}+\int_{p}F\left(X,p\right)\right)}^{2}d_{oo,F,T}\left(X,k\right), \end{array}$$

(A249) $$\begin{array}{ccc} U_{oo,\rho ,T}\left(X,k\right) & = & 4{\left(eg_{oT}\right)}^{2}{\left(|\bar{\psi }{\left(X\right)|}^{2}+\int_{p}F\left(X,p\right)\right)}^{2}d_{oo,\rho ,T}\left(X,k\right), \end{array}$$

(A250) $$\begin{array}{ccc} V_{aa,F,T}\left(X,k\right) & = & 4{\left(eg_{aT}\right)}^{2}{\left(|\bar{\psi }{\left(X\right)|}^{2}+\int_{p}F\left(X,p\right)\right)}^{2}F_{T}\left(X,k\right), \end{array}$$

(A251) $$\begin{array}{ccc} V_{aa,\rho ,T}\left(X,k\right) & = & 4{\left(eg_{aT}\right)}^{2}{\left(|\bar{\psi }{\left(X\right)|}^{2}+\int_{p}F\left(X,p\right)\right)}^{2}\rho_{T}\left(X,k\right), \end{array}$$

(A252) $$\begin{array}{ccc} V_{oo,F,T}\left(X,k\right) & = & 4{\left(eg_{oT}\right)}^{2}{\left(|\bar{\psi }{\left(X\right)|}^{2}+\int_{p}F\left(X,p\right)\right)}^{2}F_{T}\left(X,k\right), \end{array}$$

(A253) $$\begin{array}{ccc} V_{oo,\rho ,T}\left(X,k\right) & = & 4{\left(eg_{oT}\right)}^{2}{\left(|\bar{\psi }{\left(X\right)|}^{2}+\int_{p}F\left(X,p\right)\right)}^{2}\rho_{T}\left(X,k\right). \end{array}$$

We also use statistical and spectral parts in the self-energy for photons given by,

(A254) $$\begin{array}{ccc} \;\;\;\;\;\;\;\;\Pi_{F,T}\left(k\right) & = & \Pi_{F,T}^{\left(c\right)}\left(k\right)+\Pi_{F,T}^{\left(d\right)}\left(k\right)+\Pi_{F,T}^{\left(g\right)}\left(k\right)+\Pi_{F,T}^{\left(h\right)}\left(k\right), \end{array}$$

(A255) $$\begin{array}{ccc} \;\;\;\;\;\;\;\;\Pi_{\rho ,T}\left(k\right) & = & \Pi_{\rho ,T}^{\left(c\right)}\left(k\right)+\Pi_{\rho ,T}^{\left(d\right)}\left(k\right)+\Pi_{\rho ,T}^{\left(g\right)}\left(k\right)+\Pi_{\rho ,T}^{\left(h\right)}\left(k\right), \end{array}$$

(A256) $$\begin{array}{ccc} \Pi_{F,L}\left(k\right) & = & \Pi_{F,L}^{\left(c\right)}\left(k\right)+\Pi_{F,L}^{\left(g\right)}\left(k\right), \end{array}$$

(A257) $$\begin{array}{ccc} \Pi_{\rho ,L}\left(k\right) & = & \Pi_{\rho ,L}^{\left(c\right)}\left(k\right)+\Pi_{\rho ,L}^{\left(g\right)}\left(k\right), \end{array}$$

the self-energy for phonons given by,

(A258) $$\begin{array}{ccc} \;\;\;\;\;\;\;\;\Pi_{aa,F,T}\left(k\right) & = & \Pi_{aa,F,T}^{\left(e\right)}\left(k\right)+\Pi_{aa,F,T}^{\left(h\right)}\left(k\right), \end{array}$$

(A259) $$\begin{array}{ccc} \;\;\;\;\;\;\;\;\Pi_{aa,\rho ,T}\left(k\right) & = & \Pi_{aa,\rho ,T}^{\left(e\right)}\left(k\right)+\Pi_{aa,\rho ,T}^{\left(h\right)}\left(k\right), \end{array}$$

(A260) $$\begin{array}{ccc} \;\;\;\;\;\;\;\;\Pi_{oo,F,T}\left(k\right) & = & \Pi_{oo,F,T}^{\left(e\right)}\left(k\right)+\Pi_{oo,F,T}^{\left(h\right)}\left(k\right), \end{array}$$

(A261) $$\begin{array}{ccc} \;\;\;\;\;\;\;\;\Pi_{oo,\rho ,T}\left(k\right) & = & \Pi_{oo,\rho ,T}^{\left(e\right)}\left(k\right)+\Pi_{oo,\rho ,T}^{\left(h\right)}\left(k\right), \end{array}$$

(A262) $$\begin{array}{ccc} \Pi_{aa,F,L}\left(k\right) & = & \Pi_{aa,F,L}^{\left(f\right)}\left(k\right), \end{array}$$

(A263) $$\begin{array}{ccc} \Pi_{aa,\rho ,L}\left(k\right) & = & \Pi_{aa,\rho ,L}^{\left(f\right)}\left(k\right), \end{array}$$

(A264) $$\begin{array}{ccc} \Pi_{oo,F,L}\left(k\right) & = & \Pi_{oo,F,L}^{\left(f\right)}\left(k\right), \end{array}$$

(A265) $$\begin{array}{ccc} \Pi_{oo,\rho ,L}\left(k\right) & = & \Pi_{oo,\rho ,L}^{\left(f\right)}\left(k\right). \end{array}$$

Statistical and spectral parts of self-energy for photons for each diagram are,

(A266) $$\begin{array}{ccc} \;\;\;\Pi_{F,T}^{\left(c\right)}\left(k\right) & = & -\frac{e^{2}}{2m^{2}}\int_{p}\left(p^{2}-\frac{{\left(p\cdot k\right)}^{2}}{k^{2}}\right)\left(F\left(p+k\right)F\left(p\right)-\frac{1}{4}\frac{\rho (p+k)}{i}\frac{\rho (p)}{i}\right), \end{array}$$

(A267) $$\begin{array}{ccc} \Pi_{\rho ,T}^{\left(c\right)}\left(k\right) & = & -\frac{e^{2}}{2m^{2}}\int_{p}\left(p^{2}-\frac{{\left(p\cdot k\right)}^{2}}{k^{2}}\right)\left(\rho (p+k)F(p)-F(p+k)\rho (p)\right), \end{array}$$

for diagram (c) in Figure 1,

(A268) $$\begin{array}{ccc} \;\;\;\Pi_{F,T}^{\left(d\right)}\left(k\right) & = & -\frac{2e^{2}}{m^{2}}\int_{p}\left({\tilde{Q}}^{2}-\frac{{\left(\tilde{Q}\cdot k\right)}^{2}}{k^{2}}\right)\left(F\left(p+k\right)F\left(p\right)-\frac{1}{4}\frac{\rho (p+k)}{i}\frac{\rho (p)}{i}\right), \end{array}$$

(A269) $$\begin{array}{ccc} \Pi_{\rho ,T}^{\left(d\right)}\left(k\right) & = & -\frac{2e^{2}}{m^{2}}\int_{p}\left({\tilde{Q}}^{2}-\frac{{\left(\tilde{Q}\cdot k\right)}^{2}}{k^{2}}\right)\left(\rho (p+k)F(p)-F(p+k)\rho (p)\right), \end{array}$$

for diagram (d) in Figure 1,

(A270) $$\begin{array}{ccc} \Pi_{F,T}^{\left(g\right)}\left(k\right) & = & -\frac{e^{4}}{2m^{2}}{\left|\bar{\psi }\left(X\right)\right|}^{2}\int_{p}[\left(1+\frac{{\left(k\cdot (k-p)\right)}^{2}}{k^{2}{\left(k-p\right)}^{2}}\right)(F_{T}\left(k-p\right)\left(F(p+eA)+F(-p+eA)\right)\\ & & +\frac{1}{4}\frac{\rho_{T}\left(k-p\right)}{i}\left(\frac{\rho (p+eA)}{i}-\frac{\rho (-p+eA)}{i}\right))\\ & & +\left(1-\frac{{\left(k\cdot (k-p)\right)}^{2}}{k^{2}{\left(k-p\right)}^{2}}\right)(F_{L}\left(k-p\right)\left(F(p+eA)+F(-p+eA)\right)\\ & & +\frac{1}{4}\frac{\rho_{L}\left(k-p\right)}{i}\left(\frac{\rho (p+eA)}{i}-\frac{\rho (-p+eA)}{i}\right))], \end{array}$$

(A271) $$\begin{array}{ccc} \Pi_{\rho ,T}^{\left(g\right)}\left(k\right) & = & -\frac{e^{4}}{2m^{2}}{\left|\bar{\psi }\left(X\right)\right|}^{2}\int_{p}[\left(1+\frac{{\left(k\cdot (k-p)\right)}^{2}}{k^{2}{\left(k-p\right)}^{2}}\right)(\rho_{T}\left(k-p\right)\left(F(p+eA)+F(-p+eA)\right)\\ & & +F_{T}\left(k-p\right)\left(\rho (p+eA)-\rho (-p+eA)\right))\\ & & +\left(1-\frac{{\left(k\cdot (k-p)\right)}^{2}}{k^{2}{\left(k-p\right)}^{2}}\right)(\rho_{L}\left(k-p\right)\left(F(p+eA)+F(-p+eA)\right)\\ & & +F_{L}\left(k-p\right)\left(\rho (p+eA)-\rho (-p+eA)\right))], \end{array}$$

for diagram (g) in Figure 1,

(A272) $$\begin{array}{ccc} \Pi_{F,T}^{\left(h\right)}\left(k\right) & = & -2{\left(eg_{aT}\right)}^{2}{\left|\bar{\psi }\left(X\right)\right|}^{2}\int_{p}\left(1+\frac{{\left(k\cdot (k-p)\right)}^{2}}{k^{2}{\left(k-p\right)}^{2}}\right)(F_{aa,T}\left(k-p\right)\left(F(p+eA)+F(-p+eA)\right)\\ & & +\frac{1}{4}\frac{\rho_{aa,T}\left(k-p\right)}{i}\left(\frac{\rho (p+eA)}{i}-\frac{\rho (-p+eA)}{i}\right)\\ & & -2{\left(eg_{oT}\right)}^{2}{\left|\bar{\psi }\left(X\right)\right|}^{2}\int_{p}\left(1+\frac{{\left(k\cdot (k-p)\right)}^{2}}{k^{2}{\left(k-p\right)}^{2}}\right)(F_{oo,T}\left(k-p\right)\left(F(p+eA)+F(-p+eA)\right)\\ & & +\frac{1}{4}\frac{\rho_{oo,T}\left(k-p\right)}{i}\left(\frac{\rho (p+eA)}{i}-\frac{\rho (-p+eA)}{i}\right), \end{array}$$

(A273) $$\begin{array}{ccc} \Pi_{\rho ,T}^{\left(h\right)}\left(k\right) & = & -2{\left(eg_{aT}\right)}^{2}{\left|\bar{\psi }\left(X\right)\right|}^{2}\int_{p}\left(1+\frac{{\left(k\cdot (k-p)\right)}^{2}}{k^{2}{\left(k-p\right)}^{2}}\right)(\rho_{aa,T}\left(k-p\right)\left(F(p+eA)+F(-p+eA)\right)\\ & & +F_{aa,T}\left(k-p\right)\left(\rho (p+eA)-\rho (-p+eA)\right))\\ & & -2{\left(eg_{oT}\right)}^{2}{\left|\bar{\psi }\left(X\right)\right|}^{2}\int_{p}\left(1+\frac{{\left(k\cdot (k-p)\right)}^{2}}{k^{2}{\left(k-p\right)}^{2}}\right)(\rho_{oo,T}\left(k-p\right)\left(F(p+eA)+F(-p+eA)\right)\\ & & +F_{oo,T}\left(k-p\right)\left(\rho (p+eA)-\rho (-p+eA)\right)), \end{array}$$

for diagram (h) in Figure 1,

$$\begin{array}{ccc} \;\;\;\Pi_{F,L}^{\left(c\right)}\left(k\right) & = & -\frac{e^{2}}{4m^{2}}\int_{p}\frac{{\left(k^{2}+2p\cdot k\right)}^{2}}{k^{2}}\left(F\left(p+k\right)F\left(p\right)-\frac{1}{4}\frac{\rho (p+k)}{i}\frac{\rho (p)}{i}\right), \end{array}$$

(A274) $$\begin{array}{ccc} \Pi_{\rho ,L}^{\left(c\right)}\left(k\right) & = & -\frac{e^{2}}{4m^{2}}\int_{p}\frac{{\left(k^{2}+2p\cdot k\right)}^{2}}{k^{2}}\left(\rho (p+k)F(p)-F(p+k)\rho (p)\right), \end{array}$$

$$\begin{array}{ccc} \Pi_{F,L}^{\left(g\right)}\left(k\right) & = & -\frac{e^{4}}{m^{2}}{\left|\bar{\psi }\right|}^{2}\int_{p}[\left(1-\frac{{\left(k\cdot (k-p)\right)}^{2}}{k^{2}{\left(k-p\right)}^{2}}\right)(F_{T}\left(k-p\right)\left(F(p+eA)+F(-p+eA)\right)\\ & & +\frac{1}{4}\frac{\rho_{T}\left(k-p\right)}{i}\left(\frac{\rho (p+eA)}{i}-\frac{\rho (-p+eA)}{i}\right))\\ & & +\frac{{\left(k\cdot (k-p)\right)}^{2}}{k^{2}{\left(k-p\right)}^{2}}(F_{L}\left(k-p\right)\left(F(p+eA)+F(-p+eA)\right)\\ & & +\frac{1}{4}\frac{\rho_{L}\left(k-p\right)}{i}\left(\frac{\rho (p+eA)}{i}-\frac{\rho (-p+eA)}{i}\right))], \end{array}$$

(A275) $$\begin{array}{ccc} \Pi_{F,L}^{\left(g\right)}\left(k\right) & = & -\frac{e^{4}}{m^{2}}{\left|\bar{\psi }\right|}^{2}\int_{p}[\left(1-\frac{{\left(k\cdot (k-p)\right)}^{2}}{k^{2}{\left(k-p\right)}^{2}}\right)(F_{T}\left(k-p\right)\left(F(p+eA)+F(-p+eA)\right)\\ & & +\frac{1}{4}\frac{\rho_{T}\left(k-p\right)}{i}\left(\frac{\rho (p+eA)}{i}-\frac{\rho (-p+eA)}{i}\right))\\ & & +\frac{{\left(k\cdot (k-p)\right)}^{2}}{k^{2}{\left(k-p\right)}^{2}}(F_{L}\left(k-p\right)\left(F(p+eA)+F(-p+eA)\right)\\ & & +\frac{1}{4}\frac{\rho_{L}\left(k-p\right)}{i}\left(\frac{\rho (p+eA)}{i}-\frac{\rho (-p+eA)}{i}\right))],\\ \Pi_{\rho ,L}^{\left(g\right)}\left(k\right) & = & -\frac{e^{4}}{m^{2}}{\left|\bar{\psi }\right|}^{2}\int_{p}[\left(1-\frac{{\left(k\cdot (k-p)\right)}^{2}}{k^{2}{\left(k-p\right)}^{2}}\right)(\rho_{T}\left(k-p\right)\left(F(p+eA)+F(-p+eA)\right)\\ & & +F_{T}\left(k-p\right)\left(\rho (p+eA)-\rho (-p+eA)\right))\\ & & +\frac{{\left(k\cdot (k-p)\right)}^{2}}{k^{2}{\left(k-p\right)}^{2}}(\rho_{L}\left(k-p\right)\left(F(p+eA)+F(-p+eA)\right)\\ & & +F_{L}\left(k-p\right)\left(\rho (p+eA)-\rho (-p+eA)\right))]. \end{array}$$

Here we find that statistical parts of self-energy of photons are symmetric for k→−k, while spectral parts of self-energy of photons are anti-symmetric for k→−k.

Statistical and spectral parts of self-energy for phonons are,

(A276) $$\begin{array}{ccc} \;\;\;\;\Pi_{aa,F,T}^{\left(e\right)}\left(k\right) & = & -2g_{aT}^{2}\int_{p}\left(p^{2}-\frac{{\left(p\cdot k\right)}^{2}}{k^{2}}\right)\left(F\left(p+k\right)F\left(p\right)-\frac{1}{4}\frac{\rho (p+k)}{i}\frac{\rho (p)}{i}\right), \end{array}$$

(A277) $$\begin{array}{ccc} \;\Pi_{aa,\rho ,T}^{\left(e\right)}\left(k\right) & = & -2g_{aT}^{2}\int_{p}\left(p^{2}-\frac{{\left(p\cdot k\right)}^{2}}{k^{2}}\right)\left(\rho (p+k)F(p)-F(p+k)\rho (p)\right), \end{array}$$

(A278) $$\begin{array}{ccc} \;\;\;\;\;\;\Pi_{aa,F,T}^{\left(h\right)}\left(k\right) & = & -2{\left(eg_{aT}\right)}^{2}{\left|\bar{\psi }\right|}^{2}\int_{p}\left(1+\frac{{\left(k-p\cdot k\right)}^{2}}{{\left(k-p\right)}^{2}k^{2}}\right)[F_{T}\left(k-p\right)\left(F(p+eA)+F(-p+eA)\right)\\ & & +\frac{1}{4}\frac{\rho_{T}\left(k-p\right)}{i}\left(\frac{\rho (p+eA)}{i}-\frac{\rho (-p+eA)}{i}\right)], \end{array}$$

(A279) $$\begin{array}{ccc} \;\;\;\;\;\;\Pi_{aa,\rho ,T}^{\left(h\right)}\left(k\right) & = & -2{\left(eg_{aT}\right)}^{2}{\left|\bar{\psi }\right|}^{2}\int_{p}\left(1+\frac{{\left(k-p\cdot k\right)}^{2}}{{\left(k-p\right)}^{2}k^{2}}\right)[\rho_{T}\left(k-p\right)\left(F(p+eA)+F(-p+eA)\right)\\ & & +F_{T}\left(k-p\right)\left(\rho (p+eA)-\rho (-p+eA)\right)], \end{array}$$

for diagrams (e) and (h) in Figure 1,

(A280) $$\begin{array}{ccc} \;\;\Pi_{oo,F,T}^{\left(e\right)}\left(k\right) & = & -2g_{oT}^{2}\int_{p}\left(p^{2}-\frac{{\left(p\cdot k\right)}^{2}}{k^{2}}\right)\left(F\left(p+k\right)F\left(p\right)-\frac{1}{4}\frac{\rho (p+k)}{i}\frac{\rho (p)}{i}\right), \end{array}$$

(A281) $$\begin{array}{ccc} \Pi_{oo,\rho ,T}^{\left(e\right)}\left(k\right) & = & -2g_{oT}^{2}\int_{p}\left(p^{2}-\frac{{\left(p\cdot k\right)}^{2}}{k^{2}}\right)\left(\rho (p+k)F(p)-F(p+k)\rho (p)\right), \end{array}$$

(A282) $$\begin{array}{ccc} \;\;\;\;\;\Pi_{oo,F,T}^{\left(h\right)}\left(k\right) & = & -2{\left(eg_{oT}\right)}^{2}{\left|\bar{\psi }\right|}^{2}\int_{p}\left(1+\frac{{\left(k-p\cdot k\right)}^{2}}{{\left(k-p\right)}^{2}k^{2}}\right)[F_{T}\left(k-p\right)\left(F(p+eA)+F(-p+eA)\right)\\ & & +\frac{1}{4}\frac{\rho_{T}\left(k-p\right)}{i}\left(\frac{\rho (p+eA)}{i}-\frac{\rho (-p+eA)}{i}\right)], \end{array}$$

(A283) $$\begin{array}{ccc} \;\;\;\;\;\Pi_{oo,\rho ,T}^{\left(h\right)}\left(k\right) & = & -2{\left(eg_{oT}\right)}^{2}{\left|\bar{\psi }\right|}^{2}\int_{p}\left(1+\frac{{\left(k-p\cdot k\right)}^{2}}{{\left(k-p\right)}^{2}k^{2}}\right)[\rho_{T}\left(k-p\right)\left(F(p+eA)+F(-p+eA)\right)\\ & & +F_{T}\left(k-p\right)\left(\rho (p+eA)-\rho (-p+eA)\right)], \end{array}$$

(A284) $$\begin{array}{ccc} \;\;\Pi_{aa,F,L}^{\left(f\right)}\left(k\right) & = & -g_{aL}^{2}\int_{p}k^{2}\left(F\left(p+k\right)F\left(p\right)-\frac{1}{4}\frac{\rho (p+k)}{i}\frac{\rho (p)}{i}\right), \end{array}$$

(A285) $$\begin{array}{ccc} \Pi_{aa,\rho ,L}^{\left(f\right)}\left(k\right) & = & -g_{aL}^{2}\int_{p}k^{2}\left(\rho (p+k)F(p)-F(p+k)\rho (p)\right), \end{array}$$

(A286) $$\begin{array}{ccc} \;\;\Pi_{oo,F,L}^{\left(f\right)}\left(k\right) & = & -g_{oL}^{2}\int_{p}k^{2}\left(F\left(p+k\right)F\left(p\right)-\frac{1}{4}\frac{\rho (p+k)}{i}\frac{\rho (p)}{i}\right), \end{array}$$

(A287) $$\begin{array}{ccc} \Pi_{oo,\rho ,L}^{\left(f\right)}\left(k\right) & = & -g_{oL}^{2}\int_{p}k^{2}\left(\rho (p+k)F(p)-F(p+k)\rho (p)\right), \end{array}$$

for diagram (f) in Figure 1.

## Appendix F. Derivation of Time-Evolution Equations for Coherent Fields

We can derive time-evolution equations for coherent fields as follows.

Using Equation (A4), time-evolution equation for $|\bar{\psi }{\left(X\right)|}^{2}$ is,

(A288) ∂0|ψ¯|2=−∂0∫p∂ReΣR(g)+(h)(p)∂p0F(p)+ReGR(p)∂ΣF(g)+(h)(p)∂p0−∫pF(p)Σρ(g)+(h)(p)i−ρ(p)iΣF(g)+(h)(p),

where we have used the relation,

(A289) Rei2ψ¯*δΓ2δψ¯*−i2ψ¯δΓ2δψ=−∂0∫p∂ReΣR(g)+(h)(p)∂p0F(p)+ReGR(p)∂ΣF(g)+(h)(p)∂p0−∫pF(p)Σρ(g)+(h)(p)i−ρ(p)iΣF(g)+(h)(p),

in Equation (A4) due to similar calculations to Appendix in [101]. Due to Equations (A2) and (A3), we derive the constraint of $A^{0}$ as,

(A290) $$\begin{array}{ccc} e\left(A_{0}-\frac{\partial_{0}\beta }{e}\right) & = & \frac{e^{2}}{2m}{\left(A_{i}-\frac{\partial_{i}\beta }{e}\right)}^{2}+\frac{e^{2}}{2m}\int_{k}\left(2F_{T}\left(k\right)+F_{L}\left(k\right)\right)\\ & & +2e\left(g_{aT}{\bar{Q}}_{aT,i}+g_{oT}{\bar{Q}}_{oT,i}\right)\left(A_{i}-\frac{\partial_{i}\beta }{e}\right)\\ & & +eg_{aT}\left(D_{\gamma a,F,T,ii}+D_{a\gamma ,F,T,ii}\right)+eg_{oT}\left(D_{\gamma o,F,T,ii}+D_{o\gamma ,F,T,ii}\right)\\ & & -\frac{1}{2}\left(\frac{1}{\bar{\psi }}\frac{\delta \Gamma_{2}}{\delta {\bar{\psi }}^{*}}+\frac{1}{{\bar{\psi }}^{*}}\frac{\delta \Gamma_{2}}{\delta \bar{\psi }}\right), \end{array}$$

where we use Equations (A229) and (A230), and the relation,

(A291) −121ψ¯δΓ2δψ¯*+1ψ¯*δΓ2δψ¯=−e4m2∫pReGR(p+eA)PF(p)+F(p+eA)RePR(p)−8(egaT)2∫pReGR(p+eA)Pγa,F(p)+F(p+eA)RePγa,R(p)−8(egoT)2∫pReGR(p+eA)Pγo,F(p)+F(p+eA)RePγo,R(p),

with definitions,

(A292) $$\begin{array}{ccc} P^{ab}\left(x,y\right) & = & {\left(D_{ij}^{ab}\left(x,y\right)\right)}^{2}, \end{array}$$

(A293) $$\begin{array}{ccc} \;\;\;P_{\gamma a}^{ab}\left(x,y\right) & = & D_{T,ij}^{ab}\left(x,y\right)D_{aa,T,ij}^{ab}\left(x,y\right), \end{array}$$

(A294) $$\begin{array}{ccc} \;\;\;P_{\gamma o}^{ab}\left(x,y\right) & = & D_{T,ij}^{ab}\left(x,y\right)D_{oo,T,ij}^{ab}\left(x,y\right). \end{array}$$

Using Equation (A1), time-evolution equations for photon fields $A_{i}-\frac{\partial_{i}\beta }{e}$ are written by,

(A295) $$\begin{array}{ccc} \frac{\partial^{2}}{\partial {\left(x^{0}\right)}^{2}}\left(A_{i}-\frac{\partial_{i}\beta }{e}\right) & = & -\frac{e^{2}}{m}{\left|\bar{\psi }\right|}^{2}\left(A_{i}-\frac{\partial_{i}\beta }{e}\right)-\frac{e}{m}\int_{p}p_{i}F\left(X,p\right)\\ & & -2e\left(g_{aT}{\bar{Q}}_{aT,i}+g_{oT}{\bar{Q}}_{oT,i}\right)\left(|\bar{\psi }{|}^{2}+\int_{p}F\right)-\frac{1}{2}\frac{\delta \Gamma_{2}}{\delta A^{i}}, \end{array}$$

with,

(A296) 12δΓ2δAi=−e∫p∂ReΣR(c)+(e)(p)∂piF(p)+∂ΣF(c)+(e)(p)∂piReGR(p).

The relations (A5)–(A8) are rewritten by,

(A297) $$\begin{array}{ccc} \;\;\;\;\;\;\;\;\;\;\;\;\;\;\;\;\;\;\;\;\frac{\partial^{2}Q_{aL,i}}{\partial {\left(x^{0}\right)}^{2}} & = & 0, \end{array}$$

(A298) $$\begin{array}{ccc} \;\;\frac{\partial^{2}Q_{aT,i}}{\partial {\left(x^{0}\right)}^{2}}+2eg_{aT}{\left|\bar{\psi }\right|}^{2}{\left(A_{i}-\frac{\partial_{i}\beta }{e}\right)}_{T}+2g_{aT}P_{T,ij}\int_{p}p_{j}F+\frac{1}{2}\frac{\delta \Gamma_{2}}{\delta {\bar{Q}}_{aT}^{i}} & = & 0, \end{array}$$

(A299) $$\begin{array}{ccc} \;\;\;\;\;\;\;\;\;\;\;\;\;\;\;\;\;\;\frac{\partial^{2}Q_{oL,i}}{\partial {\left(x^{0}\right)}^{2}}+\Omega_{L}^{2}Q_{oL,i} & = & 0, \end{array}$$

(A300) $$\begin{array}{ccc} \frac{\partial^{2}Q_{oT,i}}{\partial {\left(x^{0}\right)}^{2}}+\Omega_{T}^{2}Q_{oT,i}+2eg_{oT}{\left(A_{i}-\frac{\partial_{i}\beta }{e}\right)}_{T}+2g_{oT}P_{T,ij}\int_{p}p_{j}F+\frac{1}{2}\frac{\delta \Gamma_{2}}{\delta {\bar{Q}}_{oT}^{i}} & = & 0, \end{array}$$

where the subscript ‘*T*’ represents transverse part and we use $P_{T,ij}=\left(\delta_{ij}-\partial_{i}\partial_{j}/\partial_{k}^{2}\right)$ and relations,

(A301) 12δΓ2δQ¯aTi=−4e2gaTgaTQ¯aT,j+goTQ¯oT,j∫pReΞR,ij(p)F(p)+ΞF,ij(p)ReGR(p),

(A302) 12δΓ2δQ¯oTi=−4e2goTgaTQ¯aT,j+goTQ¯oT,j∫pReΞR,ij(p)F(p)+ΞF,ij(p)ReGR(p),

with definition,

(A303) $$\begin{array}{ccc} \Xi_{ij}\left(x,y\right) & = & G\left(x,y\right)D_{T,ij}\left(x,y\right), \end{array}$$

(A304) $$\begin{array}{ccc} \;\;\;\;\;\;\;\;\;\Xi_{F,ij}\left(p\right) & = & \int_{k}\left(F\left(p-k\right)F_{T}\left(k\right)+\frac{1}{4}\frac{\rho (p-k)}{i}\frac{\rho_{T}\left(k\right)}{i}\right)\left(\delta_{ij}-\frac{k_{i}k_{j}}{k^{2}}\right), \end{array}$$

(A305) $$\begin{array}{ccc} \;\;\;\;\;\;\;\;\;\Xi_{\rho ,ij}\left(p\right) & = & \int_{k}\left(\rho \left(p-k\right)F_{T}\left(k\right)+F\left(p-k\right)\rho_{T}\left(k\right)\right)\left(\delta_{ij}-\frac{k_{i}k_{j}}{k^{2}}\right). \end{array}$$

## Appendix G. Conserved Energy Density

The energy density in spatially homogeneous case is written by $E_{\mathrm{tot}}=E_{\mathrm{coh}}+E_{\mathrm{qf}}+E_{\mathrm{pot},\mathrm{loc}}+E_{\mathrm{pot},\mathrm{nonl}}$.

We can write each term by,

(A306) $$\begin{array}{ccc} E_{\mathrm{coh}} & = & \frac{1}{2}{\left(\partial_{0}\left(A_{i}-\frac{\partial_{i}\beta }{e}\right)\right)}^{2}+\frac{e^{2}}{2m}{\left|\bar{\psi }\right|}^{2}{\left(A_{i}-\frac{\partial_{i}\beta }{e}\right)}^{2}\\ & & +\frac{1}{2}\left({\left(\partial_{0}{\bar{Q}}_{aT,i}\right)}^{2}+{\left(\partial_{0}{\bar{Q}}_{aL,i}\right)}^{2}+{\left(\partial_{0}{\bar{Q}}_{oT,i}\right)}^{2}+{\left(\partial_{0}{\bar{Q}}_{oL,i}\right)}^{2}+\Omega_{T}^{2}{\bar{Q}}_{oT,i}^{2}+\Omega_{L}^{2}{\bar{Q}}_{oL,i}^{2}\right)\\ & & +2e\left(g_{aT}{\bar{Q}}_{aT,i}+g_{oT}{\bar{Q}}_{oT,i}\right)\left(A_{i}-\frac{\partial_{i}\beta }{e}\right){\left|\bar{\psi }\right|}^{2}, \end{array}$$

(A307) $$\begin{array}{ccc} E_{\mathrm{qf}} & = & \int_{p}p^{0}F+\int_{k}{\left(k^{0}\right)}^{2}\left(2F_{T}+F_{L}\right)+\int_{k}{\left(k^{0}\right)}^{2}\left(2F_{aa,T}+F_{aa,L}+2F_{oo,T}+F_{oo,L}\right), \end{array}$$

(A308) Epot,loc=8(egaT)2|ψ¯|2+∫pF∫p′F∫kRedaa,R,TFT+daa,F,TReDR,T+8(egoT)2|ψ¯|2+∫pF∫p′F∫kRedoo,R,TFT+doo,F,TReDR,T−e22m∫k2FT+FL∫pF,

(A309) Epot,nonl=−13∫pReΣRF+ReGRΣF−16∫k2(ReΠR,TFT+ReDR,TΠF,T)+ReΠR,LFL+ReDR,LΠF,L−16∫k(2(ReΠaa,R,TFaa,T+ReDaa,R,TΠaa,F,T)+ReΠaa,R,LFaa,L+ReDaa,R,LΠaa,F,L)−16∫k(2(ReΠoo,R,TFoo,T+ReDoo,R,TΠoo,F,T)+ReΠoo,R,LFoo,L+ReDoo,R,LΠoo,F,L).

We have used Kadanoff–Baym equations in Section 6 with self-energy in Appendix E and time-evolution equations for coherent fields in Appendix F. For diagrams (c) and (e) in Figure 1, we have used the relation (A296). For diagrams (g) and (h) in Figure 1, we have used similar calculations to Appendix in [101].
